# Sequential alternation of nal-IRI/5-FU and gemcitabine/nab-paclitaxel versus nal-IRI/5-FU versus gemcitabine/nab-paclitaxel in first-line metastatic pancreatic cancer: results of the randomized phase II PRODIGE 61—FUNGEMAX trial (France)

**DOI:** 10.1016/j.eclinm.2026.103998

**Published:** 2026-05-29

**Authors:** Julien Taieb, Simon Pernot, Frédéric Thuillier, Alexis Delattre, Erwan Vo-Quang, Caroline Petorin, Vincent Bourgeois, David Tougeron, Franck Audemar, Carole Vitellius, Laurent Mosser, Jérôme Desrame, Frédéric Di Fiore, Yves Rinaldi, Anna Pellat, Marion Bolliet, Fabienne Watelle, Hervé Perrier, Olivier Dubreuil, Come Lepage, Jean-Baptiste Bachet

**Affiliations:** a Department of Digestive Oncology, CARPEM Comprehensive Cancer Center, Georges Pompidou European Hospital, Université Paris Cité, Paris, France; b Department of Medical Oncology, Insitute Bergognié Cancer Center, Bordeaux, France; c Service d’Oncologie Médicale, CHU de Limoges, Limoges, France; d Fédération Francophone de Cancérologie Digestive, EPICAD INSERM LNC-UMR 1231, Faculté de Médecine, University of Burgundy and Franche Comté, Dijon, France; e Service d’Oncologie Médicale, CHU Clermont Ferrand, Clermont Ferrand, France; f Service d’Oncologie Digestive, CH of Boulogne Sur Mer, Boulogne sur Mer, France; g Department of Gastroenterology and Hepatology, Poitiers University Hospital, Poitiers, France; h Service Hépato Gastroentérologie et Oncologie Digestive, Centre Hospitalier de la Cote Basque, Bayonne, France; i Gastroenterology Department, Angers Teaching Hospital, Angers, France; j Centre Hospitalier Jacques Puel, Rodez, France; k Hôpital Privé Jean Mermoz, Lyon, France; l CHU de Rouen, Rouen, France; m Department of Gastroenterology, Hôpital Européen, Marseille, France; n Gastroenterology, Endoscopy and Digestive Oncology Unit, Cochin University Hospital, Université Paris Cité, AP-HP, Paris, France; o Hepato-Gastroenterology, Colmar CH, Colmar, France; p Service D'oncologie Médicale, Centre Hospitalier LENS, LENS, France; q Oncology Department, Saint Joseph Hospital, Marseille, France; r Department of Digestive Oncology, Groupe Hospitalier Diaconesses Croix Saint Simon, Paris, France; s Dijon University Hospital, INSERM U1231, Dijon, France; t Hepatogastroenterology and Digestive Oncology Department, Pitié Salpêtrière Hospital, APHP, Sorbonne University, Paris, France

**Keywords:** Sequential treatment, Pancreatic cancer, Nal-IRI, Nab-paclitaxel, Gemcitabine

## Abstract

**Background:**

New chemotherapeutic approaches are still needed to improve survival and quality of life in metastatic pancreatic ductal adenocarcinoma (mPDAC). In the absence of validated predictive biomarkers, first-line sequential treatment strategies may allow to delay chemoresistance and reduce cumulative side effects.

**Methods:**

PRODIGE 61–FUNGEMAX was an open-label, multicentre, randomised phase II trial conducted in 31 centres in France. Between November 2018 and January 2024, chemotherapy-naive patients aged 18–75 years with histologically confirmed metastatic pancreatic ductal adenocarcinoma (mPDAC) and Eastern Cooperative Oncology Group performance status (ECOG PS) 0–1 were randomly assigned (1:1:1) to receive either nal-IRI (nanoliposomal irinotecan; 70 mg/m^2^ intravenously [IV] over 90 min) plus leucovorin (400 mg/m^2^ IV over 30 min) and 5-fluorouracil (5-FU; 2400 mg/m^2^ IV over 46 h) every 2 weeks (NAPOLI regimen), gemcitabine (1000 mg/m^2^ IV over 30 min) plus nab-paclitaxel (125 mg/m^2^ IV over 30 min) on days 1, 8, and 15 of a 28-day cycle (MPACT regimen), or both regimens alternating every 2 months (sequential arm). The primary endpoint was the 6-month progression-free survival (PFS) rate, assessed in the modified intention-to-treat (mITT)/safety population (patients who received at least one dose of study treatment). Secondary efficacy analyses were performed in the intention-to-treat (ITT) population. This trial is registered with ClinicalTrials.gov (NCT03693677) and EudraCT (2017-004309-41).

**Findings:**

Between 16 November 2018 and 25 January 2024, 288 patients were enrolled in 31 French centres. Baseline characteristics were well balanced between the three arms. With a median follow-up of 39.2 months, median treatment durations were 6.3/3.3/5.3 months, for sequential, NAPOLI and MPACT arms, respectively. The 6-month PFS rates were 51.6%, 32.3% and 45.3% in the sequential, NAPOLI and MPACT arms, respectively. Neither sequential (HR = 0.76, p = 0.072) nor NAPOLI regimens (HR = 1.20, p = 0.22) lead to a statistically significant improvement of PFS over the MPACT regimen. The 12-month PFS rates were 20·3%, 12·7%, and 11·9%, and the 24-month OS rates were 23·8%, 9·5%, and 12·5% in the sequential, NAPOLI, and MPACT arms, respectively; these differences were not statistically significant. Safety profiles were consistent with previous publications for each regimen and QoL (QLQ-C30 and EQ5D) was preserved in the sequential arm.

**Interpretation:**

The sequential NAPOLI/MPACT regimen seems feasible and tolerable, with higher rates of 12-month PFS and 24-month OS compared with standard MPACT, despite no improvement in median PFS or OS. Future trials should be adequately powered, integrate predictive biomarkers, and include contemporary first-line standards as comparators.

**Funding:**

This trial was sponsored by the Fédération Francophone de Cancérologie Digestive (FFCD) and supported by a grant from 10.13039/501100011725Servier, France.

**Research in context**

**Evidence before this study**

First-line treatment of metastatic pancreatic ductal adenocarcinoma (mPDAC) includes two multidrug standards, FOLFIRINOX and gemcitabine plus nab-paclitaxel, but long-term outcomes remain poor and cumulative toxicities limit treatment duration and quality of life. We searched PubMed/MEDLINE and Embase from database inception to November 30, 2025, using combinations of terms including “pancreatic cancer,” “metastatic,” “sequential,” “alternating,” “gemcitabine,” “nab-paclitaxel,” “liposomal irinotecan,” “nal-IRI,” “NAPOLI,” “NALIRIFOX,” “FOLFIRINOX,” and “FOLFOX,” restricted to English-language publications, and identified 5 relevant studies. Sequential or alternating chemotherapy strategies have been proposed to expose tumours to multiple non–cross-resistant agents while reducing overlapping toxicities. Prior randomised phase II trials (FIRGEM, FIRGEMAX–PRODIGE 37, GABRINOX) suggested feasibility and encouraging disease control, but evidence remained limited by small sample sizes, heterogeneous schedules, and open-label designs. Recently, the SEQUENCE trial, alternating gemcitabine plus nab-paclitaxel with mFOLFOX, reported improved survival compared with continuous gemcitabine plus nab-paclitaxel, at the cost of increased haematological toxicity. Many patients remain ineligible for intensive triplet regimens, and a substantial subset relapses after adjuvant oxaliplatin-based therapy, highlighting the need for alternative sequences avoiding oxaliplatin and limiting cumulative neuropathy.

**Added value of this study**

This trial provides the first randomised phase II evaluation of a structured, time-based alternation of nal-IRI/5-FU (NAPOLI regimen) and gemcitabine/nab-paclitaxel (MPACT regimen) every two months, compared with each regimen delivered continuously until progression, in previously untreated mPDAC. The sequential strategy was feasible, preserved QoL over time, achieved the highest objective response and disease control rates, and showed numerically improved long-term outcomes (12-month PFS and 24-month OS rates) despite not improving median PFS or median OS compared with standard MPACT.

**Implications of all the available evidence**

These findings support sequential chemotherapy as a pragmatic first-line option for selected patients with mPDAC, particularly when long-term tolerability and cumulative toxicities such as peripheral neuropathy are major concerns. While continuous gemcitabine–nab-paclitaxel remains a robust standard, early exposure to sequential chemotherapy may help sustain disease control without compromising quality of life. Future trials should adopt endpoints better aligned with the biological objective of delaying resistance (e.g., 12-month PFS, time-to-treatment-failure, durable QoL), integrate contemporary standards such as NALIRIFOX as comparators, and incorporate predictive biomarkers to identify patients most likely to benefit from early multi-agent sequencing strategies.

## Introduction

Metastatic pancreatic ductal adenocarcinoma (mPDAC) is associated with a 5-year relative survival rate below 5% and limited therapeutic options. For patients with good Eastern Cooperative Oncology Group (ECOG)-performance status (PS), two combination chemotherapy regimens, FOLFIRINOX and gemcitabine plus nab-paclitaxel, have emerged as first-line standards of care during the last decade.^1^^,^^2^ The latter, established by the MPACT trial, demonstrated improved overall survival (OS) compared to gemcitabine monotherapy, at the cost of additional toxicities, mainly hematologic adverse events, alopecia and peripheral neuropathy. Despite these advances, median OS barely exceeds one year, and cumulative toxicities remain major limitations. Thus, innovative approaches that preserve or enhance efficacy while preserving tolerability are still needed.

Several phase II studies have examined a sequential chemotherapy approach over the past 20 years. The idea behind this approach, is that alternating between different chemotherapy protocols over a short period of time, would allow the use of several molecules with no known cross-resistance, thereby improving treatment efficacy, while avoiding the accumulation of toxicity that would be observed if all these drugs were given at the same time, ultimately preserving patients' quality of life (QoL). We previously published the FIRGEM^3^ and FIRGEMAX-PRODIGE 37^4^ trials that evaluated sequences of an intensified FOLFIRI regimen (FOLFIRI.3) alternating with gemcitabine or gemcitabine plus nab-paclitaxel, respectively. Both demonstrated encouraging outcomes in terms of disease control and tolerability. Other groups explored a similar strategy^5^^,^^6^ as the recently published SEQUENCE trial that further explored this concept by alternating gemcitabine plus nab-paclitaxel with mFOLFOX.^6^ In this study, the sequential arm showed improved OS and QoL scores though increasing neutropenia and thrombocytopenia.

Liposomal irinotecan comprises irinotecan sucrosofate salt encapsulated in pegylated liposomes that protect the drug from premature conversion in the liver into its 1000 times more active metabolite, SN-38.^7^ This leads to extended circulation in plasma in patients and prolonged tumour exposure in pre-clinical tumour models compared with non-liposomal irinotecan. The clinical relevance of nanoliposomal irinotecan (nal-IRI)–based combinations in mPDAC has been demonstrated by the NAPOLI-1^8^ and NAPOLI-3^9^ phase III trials, which collectively support the integration of nal-IRI into both second-line (i.e., nal-IRI+5FU) and first-line (i.e., nal-IRI+5FU + Oxaliplatine) treatment strategies. However, NALIRIFOX, as FOLFIRINOX, is not possible in all patients due to toxicities associated with these triplet regimens and a significant number of patients recurring after adjuvant FOLFIRINOX. Thus, we have designed the PRODIGE 61—FUNGEMAX trial aiming to compare the standard MPACT study regimen to the NAPOLI-1 study regimen or a sequential approach alternating both regimens every two months.

## Methods

### Trial oversight

PRODIGE 61—FUNGEMAX was a multicentre, open-label, randomised phase II trial conducted in France under the sponsorship of the Fédération-Francophone-de-Cancérologie-Digestive (FFCD). The trial was designed as an exploratory signal-seeking study to assess the efficacy and safety of a sequential chemotherapy strategy compared to each regimen given until progression in previously untreated patients with mPDAC. The study was approved by French regulatory authorities (May 4, 2018) and the French ethic committee (CPP SUD-EST II). The study was conducted in accordance with the Declaration of Helsinki, Good Clinical Practice guidelines, the International Conference on Harmonization, and relevant French and European laws and directives. All participants provided written informed consent prior to enrolment. This study is registered with ClinicalTrials.gov (NCT03693677). Reporting was done according to the CONSORT guidelines. The full study protocol is available in Supplementary File S1.

### Patients

Main inclusion criteria were: age between 18 and 75 years, histologically/cytologically proven mPDAC, at least one measurable lesion according to the Response Evaluation Criteria in Solid Tumors (RECIST-V1.1), Eastern Cooperative Oncology Group (ECOG)-PS 0 or 1, albuminemia >30 g/l, a life expectancy greater than 12 weeks, adequate hematologic, renal, and hepatic function, and controlled pain. Previous systemic therapy for metastatic disease was not permitted; however, prior adjuvant treatment was allowed if completed more than 12 months prior to study entry. Full inclusion and exclusion criteria are listed in Supplementary Table S1.

### Randomisation and masking subsection

Patients were randomly assigned in a 1:1:1 ratio to one of the three treatment arms using a centralised, computer-generated randomisation procedure managed by the FFCD data centre. Randomisation was stratified by centre, ECOG performance status (0 versus 1), and number of metastatic sites (1 versus >1). Allocation was performed through an interactive web-response system (IWRS). This was an open-label trial; neither participants nor investigators were masked to treatment assignment.

### Trial design and treatment

Patients were randomly assigned in a 1:1:1 ratio to one of the three treatment arms with stratification according to centre, ECOG-PS (0 versus 1), and number of metastatic site (1 versus >1) (see Supplementary Fig. S1). In the MPACT arm (control arm), patients received gemcitabine (1000 mg/m^2^ IV over 30 min) plus nab-paclitaxel (125 mg/m^2^ IV over 30 min) on days 1, 8, and 15 of a 28-day cycle. In the NAPOLI arm, patients received nal-IRI (70 mg/m^2^ administered intravenously over 90 min), followed by leucovorin (400 mg/m^2^ IV over 30 min or Elvorin 200 mg/m^2^), then 5-fluorouracil (5-FU; 2400 mg/m^2^ as a continuous IV infusion over 46 h) every two weeks. Treatments were continued until disease progression, limiting toxicity or patient’s refusal.

In the sequential arm, patients received the NAPOLI regimen first for 2 months followed by the MPACT regimen for 2 months. The 2-month sequencing interval was selected to correspond to the first planned radiological evaluation (every 8 weeks), ensuring adequate exposure to each regimen to induce tumour response while limiting cumulative toxicities. This time-based alternation strategy was consistent with prior PRODIGE sequential trials and was designed to expose tumours early to non–cross-resistant agents and repeated until disease progression, unacceptable toxicity or patient’s refusal. If one regimen became intolerable or ineffective, the alternative regimen could be continued alone.

### Assessments

Complete physical examination, ECOG PS, complete blood analyses and safety assessments (NCI-CTC-AE V4.0) were performed before each chemotherapy administration. At baseline and at each tumour assessment (every 8 weeks), serum carbohydrate antigen 19-9 level and assessment of the patient’s quality of life (European Organization for Research and Treatment of Cancer (EORTC) QoL core questionnaire QLQ-C30, version 3.0) were done. Tumour assessments were performed every 8 weeks using thoraco-abdominal-pelvic CT scan or MRI when appropriate, and responses were evaluated according to RECIST version 1.1 criteria. The primary PFS analysis was based on investigator assessment. A centralised radiological review was performed for secondary endpoints. Tumour response was determined according to the RECIST V1.1.

### Study end points

The primary endpoint was PFS in the modified intent to treat/safety population (mITT/safety) population (patients who received at least one dose of treatment), assessed by investigators. Progression-free survival (PFS) was defined as the time from randomisation to documented disease progression or death from any cause, whichever occurred first. Key secondary endpoints were OS, defined as the time from randomisation to death from any cause, best objective response rate (ORR), time to treatment failure, treatment compliance (defined as delivery of a complete cycle without dose modification or delay) and time to definitive deterioration (TDD) in QoL defined as a decrease of ≥5 points in the global health status score compared to baseline, without subsequent improvement or death.

### Statistical analysis

Sample size calculations assumed a 15% absolute improvement in 6-month PFS in the experimental arms compared to control (from 30% to 45%), corresponding to a hazard ratio (HR) of 0.66. With a two-sided alpha of 5% and 80% power, 182 progressions events were required per comparison. The Schoenfeld method provides an event-based requirement. Given the 36-month follow-up period (and a primary endpoint at 6 months), it will be necessary to include as many patients as expected events. Accounting for a 5% attrition rate, a total of 288 patients (96 per arm) were to be enrolled.^2^^,^^4^

The 6-, 12-month PFS rates, the 24-month OS rates and ORR were calculated in each treatment arm along with their 95% confidence intervals (CIs). Time-to-event variables were estimated using the Kaplan–Meier method, which was chosen as the standard non-parametric approach for survival estimation in randomised oncology trials. The hazard ratios were estimated with the use of a Cox proportional-hazards model, a well-validated method for time-to-event comparisons in oncology trials. In addition, treatment effects for PFS and OS between arms, were assessed using Cox proportional hazards models, with stratification according to the baseline stratification factors. The proportional hazards assumption was assessed using graphical methods and Schoenfeld residuals which are provided for primary comparison in Supplementary Fig. S4; no major departures from proportionality were identified for the primary comparisons. Pre-specified subgroup analyses for PFS and OS were performed using Cox proportional-hazards models in the following subgroups: age (<70 versus ≥70 years), ECOG PS (0 versus 1), neutrophil-to-lymphocyte ratio (NLR; <5 versus ≥5), and prior adjuvant therapy (yes versus no). No sensitivity analyses or post-hoc analyses were performed; all reported analyses were pre-specified in the Statistical Analysis Plan (SAP).

All efficacy analyses were performed in the ITT population. This phase II screening design was powered for pairwise comparisons of each experimental arm versus the control arm. Given an overall alpha of 10%, multiplicity was managed using a Bonferroni method. Safety analyses were done in the mITT/safety population as the primary endpoint (PFS) that was also analysed in the mITT/safety population as predefined in the SAP.

No imputation was performed for missing data. The extent of missingness for key baseline variables is reported in Supplementary Table S3. For covariate-adjusted models, analyses were conducted using available-case (complete-case) data for the relevant variables. All p-values are reported to two significant figures (unless p < 0.0001). Statistical analyses were performed using SAS version 9.4 (SAS Institute, Cary, NC, USA).

### Ethics

The PRODIGE 61–FUNGEMAX trial was approved by the French regulatory authority (reference: MEDMSANAT-2018-07-00017/2017-004309-41) and by the French ethics committee (reference: 2018–26). The study was conducted in accordance with the Declaration of Helsinki, Good Clinical Practice guidelines, the International Conference on Harmonisation (ICH-E6), European Directive 2001/20/EC, and applicable French regulations. All participants provided written informed consent prior to enrolment. This trial is registered with ClinicalTrials.gov (NCT03693677) and with the European Clinical Trials Register (EudraCT number: 2017-004309-41).

### Role of the funding source

This trial was sponsored by the Fédération Francophone de Cancérologie Digestive (FFCD) and supported by a grant from Servier, France. The funders had no involvement in study design, data collection, data analyses, data interpretation, or the writing of this report. The corresponding author (JT) had full access to all data in the study and had final responsibility for the decision to submit for publication.

## Results

### Patients

**Fig. 1:**
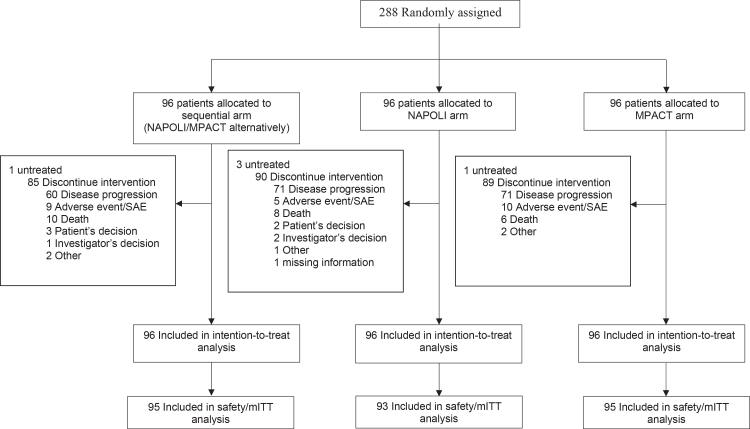
**CONSORT diagram of the FUNGEMAX-PRODIGE 61 randomised phase 2 trial**.

Between 16 November 2018 and 25 January 2024, a total of 288 patients were randomised in 31 French centres, with 96 allocated to each treatment arm. The CONSORT diagram is shown in Fig. 1. Baseline characteristics were balanced across study arms and are summarised in Table 1. Among the enrolled patients, 57.3% were male, and the median age was 65.4 years. WHO PS 1 was present in approximately two-thirds of patients across all treatment arms. Prior primary tumour resection was reported in 20 patients (7.0%). Median treatment duration was 6.3 months in the sequential arm, 3.3 months in the NAPOLI arm, and 5.3 months in the MPACT arm.

**Table 1:** Baseline characteristics of patients according to treatment arm.

	Sequential arm n = 96	NAPOLI arm n = 96	MPACT arm n = 96
Sex			
Male	51 (53.1%)	56 (58.3%)	58 (60.4%)
Female	45 (46.9%)	40 (41.7%)	38 (39.6%)
Age (years)	65.9 (59.9–70.6)	64.0 (58.0–70.2)	66.9 (59.1–72.1)
WHO PS			
0	32 (33.3%)	33 (34.4%)	36 (37.5%)
1	64 (66.7%)	63 (65.6%)	60 (62.5%)
BMI (kg/m^2^)	23.7 (21.3–26.8)	24.1 (21.4–26.7)	24.2 (21.7–27.3)
Primary tumor resection	10 (10.4%)	2 (2.1%)	8 (8.4%)
Adjuvant chemotherapy	9 (9.4%)	1 (1.0%)	8 (8.7%)
Number of metastatic sites			
1	45 (46.9%)	50 (52.1%)	50 (52.1%)
>1	51 (53.1%)	46 (47.9%)	46 (47.9%)
Metastases location			
Liver	66 (68.8%)	81 (84.4%)	86 (90.5%)
Lung	24 (25.0%)	21 (21.9%)	17 (17.9%)
Peritoneum	30 (31.3%)	21 (21.9%)	20 (21.1%)
NLR	3.57 (2.54–4.91)	3.80 (2.56–6.15)	3.39 (2.46–5.10)
<5	73 (76.0%)	60 (62.5%)	71 (74.7%)
≥5	23 (24.0%)	36 (37.5%)	24 (25.3%)
Albumin (g/L)	39.8 (37.0–43.2)	39.1 (36.0–42.9)	41.0 (37.7–43.3)
Ca 19-9 (UI/mL)	925.9 (61.7–9714.5)	2987.5 (502.1–10764)	3702.8 (351.5–19000)

Data are presented as n (%) for categorical variables and median (Q1–Q3) for continuous variables. Sequential arm: alternating nal-IRI/5-FU and gemcitabine/nab-paclitaxel every 2 months; NAPOLI arm: nal-IRI (nanoliposomal irinotecan) plus 5-fluorouracil (5-FU) and folinic acid (leucovorin); MPACT arm: gemcitabine plus nab-paclitaxel. BMI: body mass index; WHO PS: World Health Organization performance status; NLR: neutrophil-to-lymphocyte ratio; CA19-9: carbohydrate antigen 19-9.

### Efficacy

In the ITT population, the objective response rate (ORR) was higher in the sequential arm (42.7%; 95% CI: 32.7–53.2), followed by the MPACT arm (37.5%; 95% CI: 27.8–48.0) and lower in the NAPOLI arm (20.8%; 95% CI: 13.2–30.3). Disease control rates (DCR) followed the same trend (81.6% versus 77.3% versus 63.3%).

As of the data cutoff (07/01/2025), the median follow-up was 39.2 months (95% CI: 31.2- not reached (NR)).

**Fig. 2:**
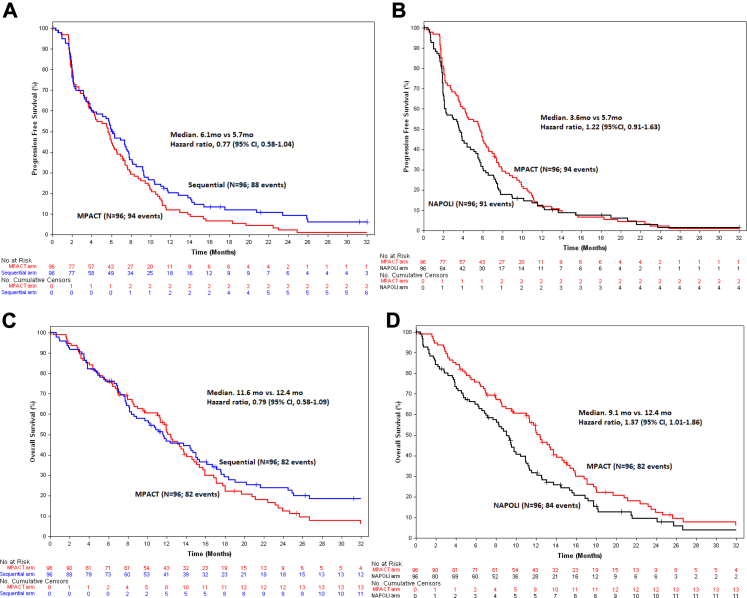
**Kaplan–Meier estimates for progression free survival and overall survival in the ITT population.** Panel A shows Kaplan–Meier estimates for progression free survival and Panel C shows Kaplan–Meier estimates for overall survival, between Sequential and MPACT arms. Panel B shows Kaplan–Meier estimates for progression free survival and Panel D shows Kaplan–Meier estimates for overall survival, between NAPOLI and MPACT arms. Abbreviations: CI: confidence interval; HR: Hazard ratios; NAPOLI: nanoliposomal irinotecan (nal-IRI) plus 5-fluorouracil (5-FU) and leucovorin; MPACT: gemcitabine plus nab-paclitaxel.

Median PFS were 6.1, 3.6 and 5.7 months in the sequential, NAPOLI and MPACT arms, respectively. HR for PFS were 0.77 [sequential versus MPACT 95% CI, 0.58–1.04; p = 0.087] and 1.22 [NAPOLI versus MPACT 95% CI, 0.91–1.63; p = 0.18] (Fig. 2 A, B). The 12-month PFS rates were 20.3% versus 12.7% and 11.9% (sequential, NAPOLI and MPACT arms).

Very similar results were seen in the mITT/safety population defined for the primary endpoint analysis in the study SAP. The primary endpoint of the study was thus not met with 6-month PFS rates of 51.6% [95% CI: 41.1–61.1], 32.3% [95% CI: 23.0–41.8] and 45.3% [95% CI: 35.1–54.9] in the sequential, NAPOLI and MPACT arms, respectively, in the mITT/safety population and median PFS of 6.2, 3.7 and 5.7 months. HR for PFS were 0.76 [sequential versus MPACT 95% CI, 0.57–1.03; p = 0.073] and 1.29 [NAPOLI versus MPACT 95% CI, 0.90–1.60; p = 0.22] (Supplementary Fig. S2).

Altogether, 248 (86.1%) deaths occurred: 82 (85.4%) in the sequential arm, 84 (87.5%) in the NAPOLI arm, and 82 (85.4%) in the MPACT arm. Median OS were 11.6 (95% CI, 8.6–15.2), 9.1 (95% CI, 7.2–10.9) and 12.4 months (95% CI, 11.3–14.6) in the sequential, NAPOLI and MPACT arms, respectively. The HR for death was 0.79 (95% CI, 0.58–1.09; p = 0.16) for the sequential versus MPACT comparison and 1.37 (95% CI, 1.01–1.86; p = 0.045) for the NAPOLI versus MPACT comparison (Fig. 2 C,D). 24-month OS rates were 23.8% (95% CI, 15.4–33.2), 9.5% (95% CI, 4.2–17.4) and 12.5% (95% CI, 6.3–20.9) in the sequential, NAPOLI and MPACT arms, respectively.

**Fig. 3:**
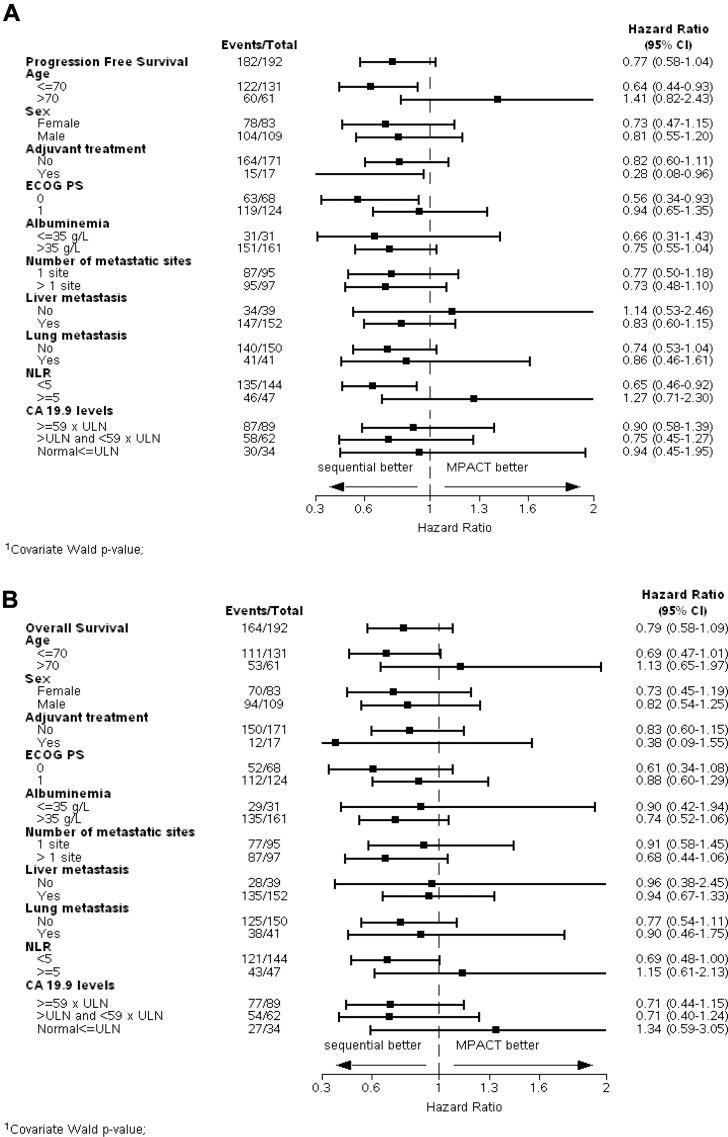
**This exploratory forest plot analysis is in key prespecified subgroups in the intention-to-treat population for progression free (A) and overall survival (B).** The dashed line indicates the point of no effect (hazard ratio = 1). A Cox proportional-hazards model was used to calculate hazard ratios and 95% confidence intervals and to assess the magnitude of the treatment difference between arms. A hazard ratio of less than one implies a lower risk of death with nal-IRI plus 5-FU followed by *nab*-paclitaxel plus gemcitabine than with *nab*-paclitaxel plus gemcitabine alone. Since there was no control for multiplicity, the confidence intervals should not be considered clinically directive. Abbreviations: ULN: Upper limit of normal; CA 19-9: carbohydrate antigen 19-9; CI: confidence interval; ECOG: Eastern Cooperative Oncology Group.

Pre-defined subgroups analyses showed better PFS in the sequential arm compared to the MPACT arm in patients aged under 70 years (HR = 0.64, 95% CI, 0.44–0.93), with an ECOG PS of 0 (HR = 0.56, 95% CI, 0.34–0.93; p = 0.03), a neutrophil-to-lymphocyte ratio (NLR) < 5 (HR = 0.64, 95% CI, 0.45–0.92; p = 0.01), and who had received prior adjuvant therapy (HR = 0.28, 95% CI, 0.08–0.96; p = 0.04), and a better OS in patients with a NLR< 5 (HR = 0.68, 95% CI, 0.47–0.99; p = 0.04) (Fig. 3). Subgroups analyses comparing the NAPOLI with the MPACT arm are shown in Supplementary Fig. S3.

Among patients who discontinued treatment, 46.3% in the sequential arm, 67.7% in the NAPOLI, and 71.6% in the MPACT arm received subsequent systemic therapy, mostly FOLFOX (Supplementary Table S2).

### Safety

A summary of safety is provided in Table 2. Grade 3–4 adverse events occurred in 85.3%, 53.8% and 69.5% of patients in the sequential, NAPOLI and MPACT arms respectively. The most frequent non-haematologic grade ≥3 toxicities were diarrhea (17.9%, 16.1%, and 5.3%, respectively) and vomiting (15.8%, 14% and 4.2%, respectively). Peripheral sensory-motor neuropathy of grade 3–4 occurred in 5.3% of patients in the sequential arm, none in the NAPOLI arm, and 8.4% in the MPACT arm. Permanent discontinuation of gemcitabine or nab-paclitaxel due to a SAE occurred in 4.5% versus 7.9% in the sequential and MPACT arms, respectively. Permanent discontinuation of nal-IRI due to a SAE was reported in 7.1% versus 2.2% in the sequential and NAPOLI arms.

**Table 2:** Treatment-emergent adverse events reported throughout the trial, by treatment arm (safety population).

Adverse event	Sequential arm n = 95		NAPOLI arm n = 93		MPACT arm n = 95	
Grade	All grade	3–4	All grade	3–4	All grade	3–4
At least 1 event	94 (98.9%)	81 (85.3%)	91 (97.8%)	50 (53.8%)	92 (96.8%)	66 (69.5%)
Hematological						
Anemia	84.2%	6.3%	69.9%	2.2%	86.3%	4.2%
Febrile Neutropenia	0%	0%	0%	0%	0%	0%
Neutropenia	61.1%	35.8%	23.7%	9.7%	51.6%	35.8%
Gastro-intestinal						
Diarrhea	80.0%	17.9%	69.9%	16.1%	55.8%	5.3%
Nausea/Vomiting	80.0%	15.8%	72.0%	14.0%	61.1%	4.2%
Skin toxicity	50.5%	1.1%	36.6%	1.1%	70.5%	5.3%
Infection	5.3%	3.2%	6.5%	1.1%	13.7%	2.1%
Venous thromboembolism	4.2%	1.1%	4.3%	1.1%	11.6%	5.3%
Peripheral neuropathy	33.5%	5.3%	10.8%	0%	47.9%	8.4%

Data are presented as percentage of patients experiencing at least one event of the specified type and grade. Adverse events were graded according to the National Cancer Institute Common Terminology Criteria for Adverse Events (NCI-CTCAE), version 4.0. Sequential arm (n = 95): alternating nal-IRI/5-FU and gemcitabine/nab-paclitaxel every 2 months; NAPOLI arm (n = 93): nal-IRI (nanoliposomal irinotecan) plus 5-fluorouracil and folinic acid; MPACT arm (n = 95): gemcitabine plus nab-paclitaxel. The safety population comprises all patients who received at least one dose of study treatment. Skin toxicity includes alopecia and all-grade cutaneous reactions.

### Quality of life

**Fig. 4:**
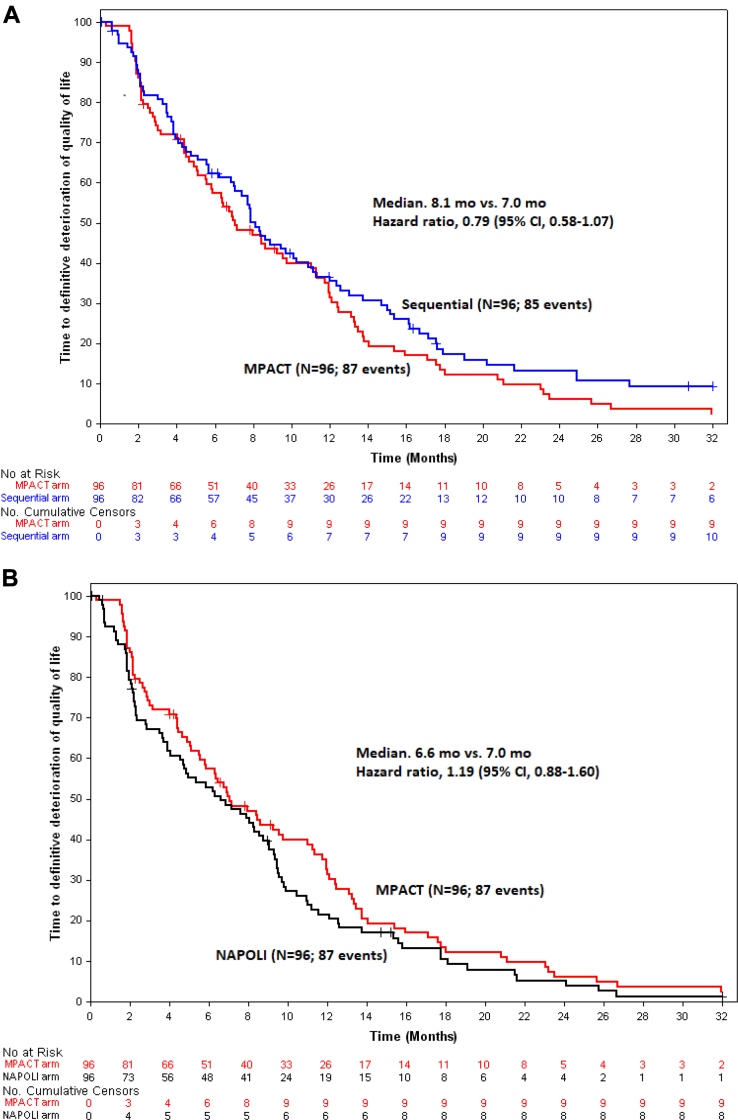
**Kaplan–Meier estimates for time to definitive deterioration of quality of life.** Panel A shows Kaplan–Meier estimates for TDD between Sequential and MPACT arms. Panel B shows Kaplan–Meier estimates for TDD between NAPOLI and MPACT arms. Abbreviations: CI: confidence interval; TDD: Time to Definitive Deterioration.

According to the EORTC QLQ-C30, a QoL deterioration ≥5 points without improvement was observed in 32.6%, 22.6%, and 31.6% of patients in the sequential, NAPOLI, and MPACT arms, respectively. Median durations of TDD were 8.1 months (95% CI, 7.0–11.1), 6.6 months (95% CI, 4.7–9.1), and 7.0 months (95% CI, 5.8–11.3), respectively. The proportion of patients without QoL deterioration at 6 months was 63.1% (95% CI, 52.3–72%) in the sequential, 54.1% (95% CI, 43.2–63.8%) in the NAPOLI, and 57.3% (95% CI, 46.6–66.8%) in the MPACT arm (Fig. 4).

## Discussion

In this randomised phase II trial, we compared three first-line treatment strategies in mPDAC: a sequential approach alternating NAPOLI and MPACT regimens, continuous NAPOLI regimen, and continuous standard MPACT regimen. The aim was to evaluate whether a sequential therapeutic strategy could provide clinical benefit by optimising efficacy while potentially reducing toxicity.

The sequential arm achieved the highest objective response rate (42.7%) and disease control rate (81.6%), with a non-significant improvement in PFS compared with the standard first-line MPACT regimen (HR 0.76, p = 0.07). Median OS was similar between the sequential and MPACT arms; however, long-term PFS (12 months) and OS (24 months) rates showed a non-significant trend favoring the sequential strategy. Pre-planned subgroup analyses suggested that the trend toward improved PFS and OS in the sequential arm was consistent across nearly all subgroups, except for patients with an NLR ≥5.

Post-study anticancer treatment was given in 40%–65% of patients, which is similar to that seen in several recent studies following MPACT regimen failure.^2^^,^^4^^,^^10^ A higher percentage of patients receiving post-study treatment could contribute to a longer survival. The sequential strategy was associated with numerically higher 12-month PFS and 24-month OS rates; however, these findings should be interpreted cautiously given the absence of statistically significant differences in median survival and the exploratory phase II design. The 24% two-year OS rate observed with the sequential regimen compares favourably with most randomised 1st line mPDAC trials.^1^^,^^2^ Nevertheless, here, about 70% of patients in the MPACT arm received second-line treatment, and a substantial proportion were exposed to platinum-based regimens (FOLFIRINOX or FOLFOX). Such intensive post-progression therapy may have contributed to prolonged overall survival without proportionally affecting early PFS landmarks.

Conversely, the NAPOLI arm was consistently numerically inferior to the MPACT arm in ORR, PFS and OS, with a statistically significant disadvantage in OS (HR 1.37, p = 0.045). These results support its biological relevance beyond second-line use, although they do not establish it as equivalent or superior to gemcitabine–nab-paclitaxel.

Other recent studies have investigated sequential strategies in mPDAC. The SEQUENCE trial reported improved 12-month survival with a MPACT/mFOLFOX sequence versus standard MPACT (55.3% versus 35.4%).^6^ The FIRGEMAX-PRODIGE 37 study, alternating MPACT with modified FOLFIRI.3, and the GABRINOX study, alternating MPACT with FOLFIRINOX, also yielded promising results.^3^^,^^5^ However, these regimens incorporate oxaliplatin or irinotecan, whose efficacy and tolerability profiles may differ in first-line therapy, as suggested by prior second-line data. Another trial alternating NAPOLI with FOLFOX showed a clinically meaningful trend favoring a sequential approach.^11^ Notably, the recent NAPOLI-3 trial demonstrated superiority of NALIRIFOX over gemcitabine/nab-paclitaxel in the first-line setting, potentially redefining the standard of care and supporting future sequential approaches integrating nal-IRI/5-FU plus oxaliplatin after gemcitabine/nab-paclitaxel in appropriate patients.^9^^,^^12^

Recent transcriptomic analyses of primary tumours have revealed heterogeneous tumour-cell sensitivity to commonly used cytotoxic agents in PDAC, with some cells resistant to all drugs, some sensitive to one agent, and others sensitive to several.^13^^,^^14^ This has led to the development of predictive transcriptomic signatures currently under evaluation,^15^ which may help guide individualised therapy. However, most data come from resected PDAC samples in the adjuvant setting, and robust evidence in metastatic disease is still lacking. Moreover, recent studies reveal substantial intra-tumoural heterogeneity, with multiple subtypes coexisting within a single tumour. This heterogeneity drives variable chemosensitivity across time and space, potentially limiting the usefulness of baseline transcriptomic signatures in advanced disease.^16^ Until predictive biomarkers are validated, sequential strategies that expose mPDAC to multiple cytotoxic agents within a shorter timeframe remain relevant.

From a tolerability standpoint, the sequential strategy appears feasible despite combining two active regimens in a structured sequence. This approach may offer advantages when reducing cumulative toxicity is a priority, as it separates drug exposures and avoids overlapping toxicities. The omission of oxaliplatin may also benefit patients previously treated with oxaliplatin-based adjuvant therapy who have residual neuropathy. Unfortunately, only 18 of 288 patients had received adjuvant FOLFIRINOX, limiting conclusions for this subgroup.

The sequential arm had a manageable safety profile despite more frequent high-grade toxicities. Using two chemotherapy regimens with distinct toxicity profiles inevitably increases the overall number of reported events. Compared to standard MPACT, the sequential strategy primarily increased gastrointestinal toxicities related to NAPOLI. However, peripheral neuropathy—an important dose-limiting toxicity of nab-paclitaxel—was less frequent and less severe in the sequential arm, supporting the rationale for time-separated regimens to reduce chronic toxicities. Alternating therapies may permit recovery periods—for example, neuropathy improvement during 5-FU-based cycles and gastro-intestinal recovery during gemcitabine-based cycles. This strategy may be particularly valuable for patients requiring prolonged treatment or those at risk of cumulative toxicities (e.g., pre-existing neuropathy). Interestingly, despite higher reported toxicity in the sequential arm, QoL TDD numerically favored the sequential approach, with a median TDD of 8.1 months versus 7.0 and 6.6 months in the MPACT and NAPOLI arms, respectively. From another perspective, emerging therapeutic strategies in pancreatic cancer, including anti-KRAS, antibody-drug conjugates, targeted therapies, and immunomodulatory combinations, may be challenging to combine with full-dose triplet chemotherapy. Sequential doublet-based approaches may therefore offer a pragmatic backbone for integration of novel agents while preserving tolerability.

Though all drugs used in this trial are currently approved for use in mPDAC and the sequence may be interesting is selected patients, this study has several limitations. First, the trial was not powered or designed to demonstrate non-inferiority or for definitive comparisons. So numerical similarities between the sequential and MPACT arm should be interpreted with caution and definitive comparisons in overall survival should be considered hypothesis-generating rather than practice-changing. Second, the absence of a centralised review of response could have introduced assessment bias, although objective endpoints were rigorously monitored according to standard international evaluation criteria. Third, the trial population included only patients aged ≤75 years with ECOG PS 0–1, potentially limiting generalizability to older or frailer individuals, in which a sequential treatment could be of particular interest. Fourth we chose a 6 months PFS rate to design the study that may not be optimal, as sequential approaches have the goal to delay resistance to chemotherapy and improve long-term disease control and OS. Fifthly, the trial was initiated before the publication of NAPOLI-3, which has since shifted the treatment paradigm and raised the bar for first-line efficacy. In addition, the trial did not specifically enrol patients deemed ineligible for triplet therapy. Therefore, these findings should not be interpreted as replacing triplet regimens in fit patients. Finally, although baseline characteristics were generally balanced across arms, the NAPOLI arm included a numerically higher proportion of patients with adverse prognostic factors (higher CA19-9 levels, higher NLR, and lower proportion with prior primary tumour resection). These imbalances may have partly contributed to the inferior PFS and OS observed in this group.

In conclusion, the PRODIGE 61–FUNGEMAX trial demonstrates the feasibility of a structured sequential chemotherapy strategy in first-line mPDAC. Although it did not improve median PFS or OS compared with standard MPACT, the favourable long-term survival rates and preserved quality of life justify further evaluation in adequately powered phase III trials. Selected patients—particularly those who are less fit or those previously treated with adjuvant FOLFIRINOX who have residual neuropathy, where cumulative toxicity and long-term tolerability are critical considerations might benefit from this sequential chemotherapy strategy. By contrast, continuous NAPOLI demonstrated insufficient efficacy, underscoring the importance of carefully selecting backbone regimens for future sequential approaches. Further research is needed to define the optimal sequencing and timing of therapies to improve long-term outcomes and tolerability in mPDAC.

## Contributors

JT conceived and designed the study. JT, SP, EVQ, and JBB drafted the manuscript. AD performed all statistical analyses. JT and AD accessed and verified the underlying data. FT, CP, VB, DT, FA, CV, LM, JD, FDF, YR, AP, MB, FW, HP, OD, and CL enrolled patients, collected clinical data, and contributed to data interpretation. SP, EVQ, and JBB critically revised the manuscript for important intellectual content. All authors read and approved the final version of the manuscript.

## Data sharing statement

Individual participant data underlying the results reported in this article will not be made publicly available, owing to legal and ethical restrictions under the FFCD sponsor agreement and applicable French data protection regulations (CNIL). De-identified aggregate data may be made available upon reasonable request to the corresponding author (JT), subject to approval by the FFCD data governance committee and applicable regulatory constraints. The statistical analysis code (SAS version 9.4) used for the primary and secondary analyses is available upon reasonable request to the corresponding author.

## Declaration of interests

JT reports honoraria for lectures and speakers' bureau activities from Amgen, Astellas, Bristol-Myers Squibb, Merck Sharp & Dohme, Novartis, Pierre Fabre, and Servier, and support for travel from Amgen and Astellas. SP reports honoraria for lectures from Astellas, Incyte, Servier, Bayer, Amgen, Merck, Pierre Fabre, AstraZeneca, MSD, BMS, and Takeda, and support for travel from MSD, Amgen, Merck, Pierre Fabre, and Takeda. DT reports honoraria for lectures from Roche, Astellas, Incyte, Servier, Bayer, Amgen, Merck, Pierre Fabre, AstraZeneca, MSD, BMS, Takeda, and Daiichi Sankyo, and support for travel from Servier, MSD, Amgen, Merck, Pierre Fabre, and Takeda. VB reports consulting fees from Pierre Fabre, Servier, and BMS, support for travel from Servier, MSD, Viatris, and Takeda, and participation on advisory boards for Servier and AstraZeneca. FA reports honoraria for lectures from Servier, Amgen, BMS, MSD, Pierre Fabre, and Merck Serono, and support for travel from Servier, Amgen, and Pierre Fabre. OD reports honoraria for lectures from AstraZeneca, BMS, MSD, Astellas, Merck Serono, and Servier, and support for travel from MSD, Daiichi Sankyo, Merck Serono, Servier, and Pierre Fabre. AP reports consulting fees from Servier, Takeda, MSD, and Merck, honoraria for lectures from Ipsen, and support for travel from Merck, Servier, and AstraZeneca. JBB reports consulting fees from AbbVie, Amgen, Bristol-Myers Squibb, Incyte, GlaxoSmithKline, Merck Serono, Merck Sharp & Dohme, Novocure, Nutricia, Servier, and Takeda, honoraria for lectures from AbbVie, Amgen, Bristol-Myers Squibb, Incyte, GlaxoSmithKline, Leo Pharma, Merck Serono, Merck Sharp & Dohme, Pierre Fabre, Servier, and Takeda, support for travel from Amgen, Merck Serono, MSD, Sanofi, and Servier, and participation on advisory boards for Servier and GSK. CL reports honoraria for lectures from Amgen, AAA, Astellas, Pierre Fabre, and Natera, support for travel from Pierre Fabre, Ipsen, and Novartis, and participation on advisory boards for AbbVie, Ipsen, and Pierre Fabre. FT, AD, EVQ, CP, CV, LM, JD, FDF, YR, MB, FW, and HP declare no competing interests.

## Appendix A Supplementary data

Supplementary Figs. S1–S4 and Tables S1–S3

Protocol
