# Supplementary material for: Sequential alternation of nal-IRI/5-FU and gemcitabine/nab-paclitaxel versus nal-IRI/5-FU versus gemcitabine/nab-paclitaxel in first-line metastatic pancreatic cancer: results of the randomized phase II PRODIGE 61—FUNGEMAX trial (France)
Source: eClinicalMedicine. 2026 May 29;96:103998. doi: 10.1016/j.eclinm.2026.103998 (PMC13240766; doi:10.1016/j.eclinm.2026.103998)
Supplement: Supplementary Figs. S1–S4 and Tables S1–S3 [file mmc1.docx]

**Supplementary materials -Appendix**

**Supplementary Table 1. Inclusion and exclusion criteria**

| **Inclusion criteria** | - Histopathologically or cytologically proven pancreatic adenocarcinoma (on primitive or metastatic lesion) - Metastatic disease at a distance - At least one measurable lesion according RECIST v1.1 criteria - 18 ≤ age ≤ 75 years - Life expectancy >12 weeks - Performance status WHO < 2 - No prior chemotherapy: adjuvant chemotherapy by gemcitabine +/- capecitabine is allowed if ended at least 12 months before the inclusion and adjuvant or neo-adjuvant FOLRIFINOX chemotherapy is allowed if ended at least 12 months prior the inclusion - Pain well controlled before the inclusion of the patient - ANC ≥ 1,500 cells/μL (without the use of hematopoietic growth factors); platelet count ≥ 100,000 cells/μL, hemoglobin ≥ 9 g/dL (blood transfusions is permitted for patients with hemoglobin levels below 9 g/dL) - Adequate hepatic function as evidenced by: Serum total bilirubin within normal range for the institution (Serum bilirubin ≤ 1,5 UNL) Biliary drainage allowed for biliary obstruction. - Albumin levels ≥ 3.0 g/dL - Aspartate aminotransferase (AST) and alanine aminotransferase (ALT) ≤ 2.5 x ULN (≤ 5 x ULN acceptable if liver metastases were present) - Normal renal function test (creatinine clearance ≥ 50 ml/min) - Normal ECG or ECG without any clinically significant findings - Patient able to understand and sign an informed consent - Females of child-bearing potential are required to test negative for pregnancy at the time of enrollment based on a urine or serum pregnancy test. - Both male and female patients of reproductive potential were required to agree to use a reliable method of birth control, during the study and for 7 months following the last dose of study drug. - Patient affiliated to social security - Regular follow-up possible |
| --- | --- |
| **Non-inclusion criteria** | - Uncontrolled brain or meningeal metastasis, or bone metastasis (no need of systematic CT scan) - Prior radiation therapy (except if there is at least one measurable target outside irradiation area) - Clinically significant gastrointestinal disorder including hepatic disorders, bleeding, inflammation, occlusion, or diarrhea > Grade 1 - History of chronic inflammatory bowel disease - Other types of pancreatic tumours, in particular endocrine or acinar cell tumours - Ampulloma - Gilbert's syndrome - Presence of neuropathy > grade 1 according to NCI-CTC - History of any second malignancy in the last 5 years; subjects with prior history of *in-situ* cancer or basal or squamous cell skin cancer are eligible. Subjects with other malignancies are eligible if they had been continuously disease free for at least 5 years. - Severe arterial thromboembolic events (myocardial infarction, unstable angina pectoris, stroke) less than 6 months before inclusion. - NYHA Class III or IV congestive heart failure, ventricular arrhythmias or uncontrolled blood pressure. - Known hypersensitivity to any of the drugs /constituents or non-lipososomal irinotecan - Any other medical or social condition deemed by the investigator to be likely to interfere with a patient’s ability to sign informed consent, cooperate and participate in the study, or interferes with the interpretation of the results. - Use of CYP3A4/UGT1A inducers/inhibitors - Use of strong CYP2C8 inhibitors or inducers, or presence of any other contraindications for nab-paclitaxel or gemcitabine - ILD presence - Partial or complete DPD deficiency (Uracilemia ≥ 16 ng/ml) - Pregnant or breast feeding |

**Supplementary Table 2. Post study treatment in each study arm**

| Subsequent line | Sequential arm  n=95 | NAPOLI arm  n=93 | MPACT arm  n=95 |
| --- | --- | --- | --- |
| At least 1 subsequent line | 44 (46.3%) | 63 (67.7%) | 68 (71.6%) |
| 2^nd^ line treatment regimens  FOLFOX  FOLFIRINOX  FOLFIRI  GEMCITABINE + (NAB)PACLITAXEL  GEMCITABINE  Other | 38.1%  19.0%  7.1%  7.1%  14.3%  12.0% | 16.1%  17.7%  1.6%  43.5%  14.5%  6.4% | 23.5%  55.9%  11.8%  4.4%  0.0%  4.5% |

|  | Sequential arm  n=95 | NAPOLI arm  n=93 | MPACT arm  n=95 |
| --- | --- | --- | --- |
|  | N = 44 | N = 63 | N = 68 |
| n | 42 | 62 | 68 |
| CAPECITABINE | 0 (0.0) | 1 (1.6%) | 0 (0.0) |
| DURVALUMAB + TREMELIMUMAB + OLAPARIB | 1 (2.4%) | 0 (0.0) | 0 (0.0) |
| FOLFIRI | 3 (7.1%) | 1 (1.6%) | 8 (11.8%) |
| FOLFIRINOX | 8 (19.0%) | 11 (17.7%) | 38 (55.9%) |
| FOLFOX | 16 (38.1%) | 10 (16.1%) | 16 (23.5%) |
| FOLFOX + OLAPARIB | 0 (0.0) | 0 (0.0) | 1 (1.5%) |
| GEMCITABINE | 6 (14.3%) | 9 (14.5%) | 0 (0.0) |
| GEMCITABINE + CAPECITABINE | 1 (2.4%) | 0 (0.0) | 0 (0.0) |
| GEMCITABINE + NABPACLITAXEL | 3 (7.1%) | 23 (37.1%) | 3 (4.4%) |
| GEMCITABINE + PLATINE | 1 (2.4%) | 0 (0.0) | 0 (0.0) |
| GEMCITABINE NABPACLITAXEL | 0 (0.0) | 2 (3.2%) | 0 (0.0) |
| GEMOX | 2 (4.8%) | 2 (3.2%) | 0 (0.0) |
| LV5FU2 | 1 (2.4%) | 0 (0.0) | 0 (0.0) |
| NAB-PACLITAXEL | 0 (0.0) | 1 (1.6%) | 0 (0.0) |
| NAL-IRI + LV5FU2 | 0 (0.0) | 0 (0.0) | 1 (1.5%) |
| PACLITAXEL + GEMCITABINE | 0 (0.0) | 2 (3.2%) | 0 (0.0) |
| XELODA + RADIOTHERAPIE 50 GY | 0 (0.0) | 0 (0.0) | 1 (1.5%) |

**Supplementary Table 3.** Missing data at baseline according to treatment arm.

| **Baseline variable** | **Sequential arm n=96** | **NAPOLI arm n=96** | **MPACT arm n=96** |
| --- | --- | --- | --- |
| Sex | No missing data | No missing data | No missing data |
| Age (years) | No missing data | No missing data | No missing data |
| WHO performance status | No missing data | No missing data | No missing data |
| BMI (kg/m²) | No missing data | No missing data | **1 (1.0%)** |
| Primary tumour localisation | No missing data | **1 (1.0%)** | **1 (1.0%)** |
| Primary tumour resection | No missing data | No missing data | **1 (1.0%)** |
| Adjuvant chemotherapy | No missing data | No missing data | **4 (4.2%)** |
| Number of metastatic sites | No missing data | No missing data | No missing data |
| Metastases location | No missing data | No missing data | **1 (1.0%)** |
| UGT1A1*28 polymorphism | No missing data | No missing data | **1 (1.0%)** |
| Dihydropyrimidine dehydrogenase deficiency (DPD) | No missing data | No missing data | **1 (1.0%)** |
| Neutrophil-to-lymphocyte ratio (NLR) | No missing data | No missing data | **1 (1.0%)** |
| Albumin (g/L) | No missing data | No missing data | No missing data |
| CA19-9 (UI/mL) | **4 (4.2%)** | **4 (4.2%)** | **3 (3.1%)** |

Data are presented as n (%) for variables with missing values. **Sequential arm:** alternating nal-IRI/5-FU and gemcitabine/nab-paclitaxel every 2 months; **NAPOLI arm:** nanoliposomal irinotecan (nal-IRI) plus 5-fluorouracil (5-FU) and leucovorin; **MPACT arm:** gemcitabine plus nab-paclitaxel. **BMI:** body mass index; **WHO PS:** World Health Organization performance status; **NLR:** neutrophil-to-lymphocyte ratio; **CA19-9:** carbohydrate antigen 19-9; **DPD:** dihydropyrimidine dehydrogenase; **UGT1A1*28:** uridine diphosphate glucuronosyltransferase 1A1 polymorphism. No imputation was performed for missing data; covariate-adjusted models used available-case (complete-case) analysis.

**Colour key:** Green = no missing data; Orange = missing values present (n [%]).

**Suppl Figure 1. study design and treatment regimens**


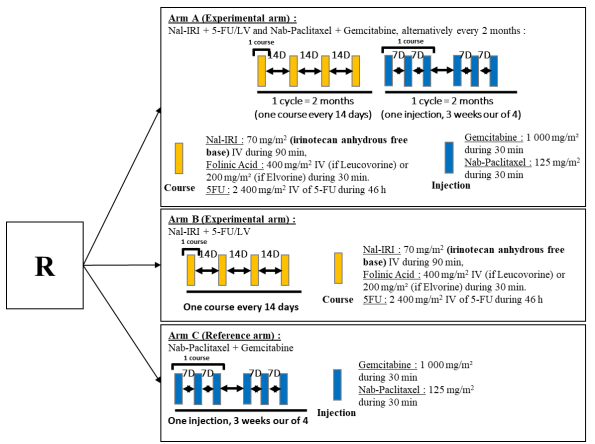


**Suppl Figure 2. Kaplan–Meier Estimates for Progression Free Survival in the mITT population (receiving at least one dose of treatment).**

Panel A shows Kaplan–Meier estimates for progression free survival between Sequential and MPACT arms.

Panel B shows Kaplan–Meier estimates for progression free survival between NAPOLI and MPACT arms.

CI denotes confidence interval.

A


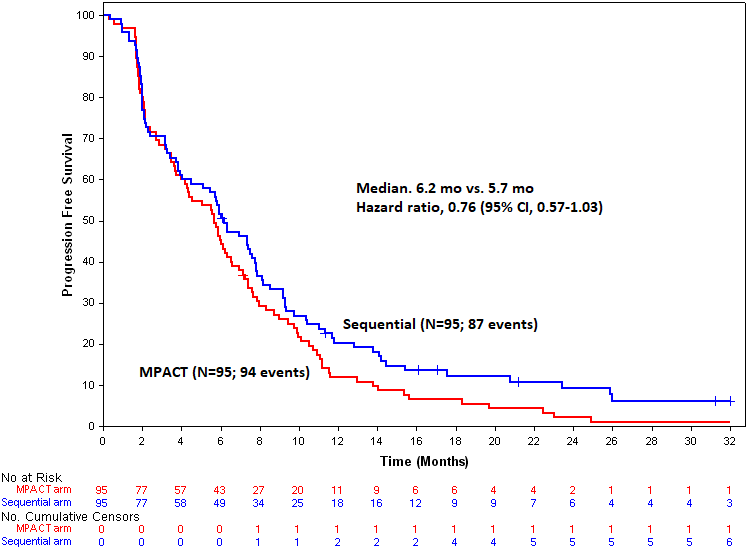


B


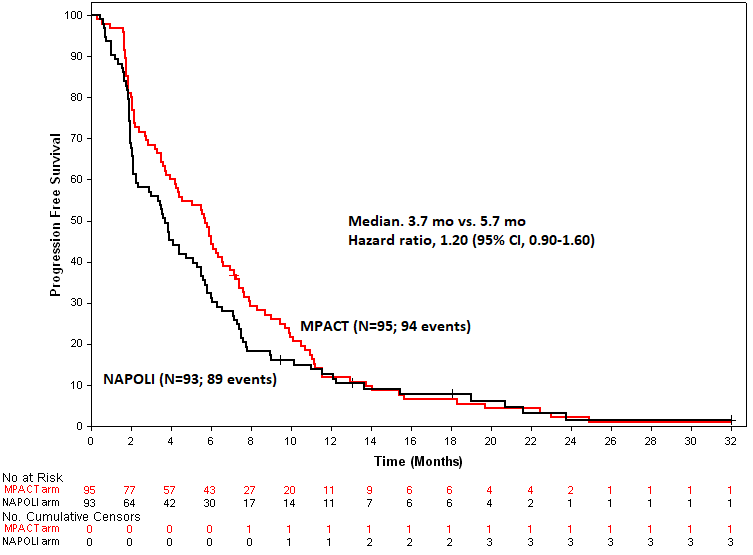


**Suppl Figure 3.** This exploratory **forest plot analysis** is in key prespecified subgroups in the intention-to-treat population for progression free (A) and overall survival (B). The dashed line indicates the point of no effect (hazard ratio=1). A Cox proportional-hazards model was used to calculate hazard ratios and 95% confidence intervals and to assess the magnitude of the treatment difference between arms. A hazard ratio of more than one implies a higher risk of death with nal-IRI plus 5FU than with *nab*-paclitaxel plus gemcitabine. Since there was no control for multiplicity, the confidence intervals should not be considered clinically directive. *Upper limit of normal. CA 19-9 denotes carbohydrate antigen 19-9; CI, confidence interval; ECOG, Eastern Cooperative Oncology Group; and ULN, upper and lower limits.

A


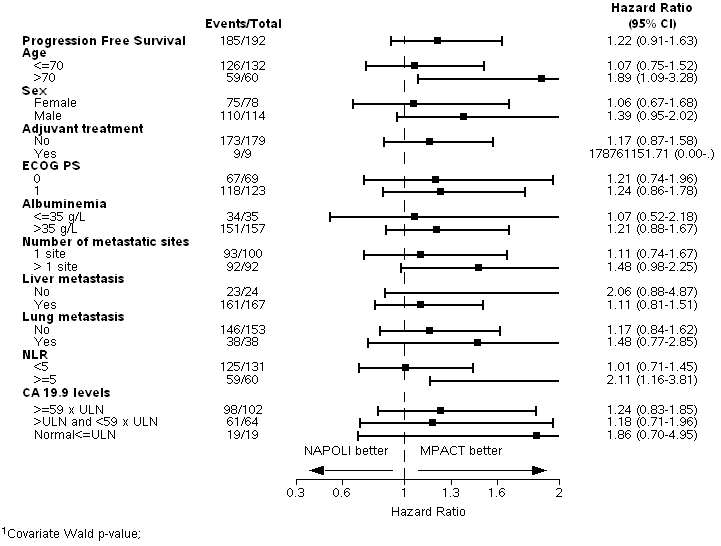


B


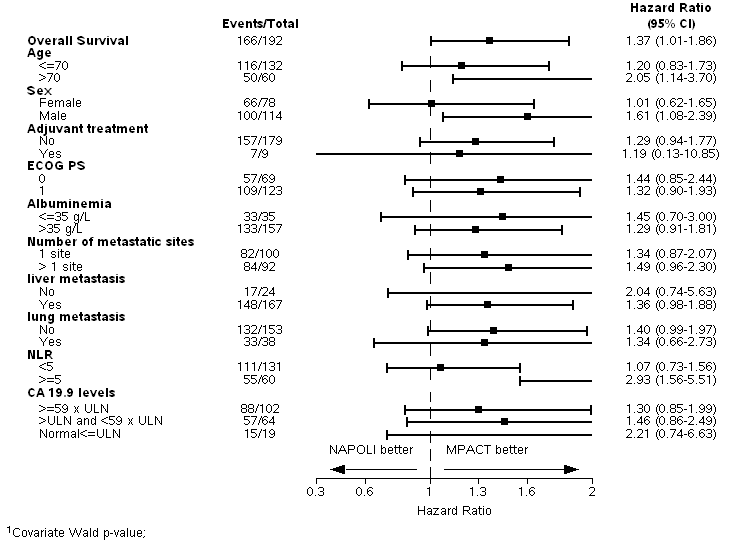


# **Suppl Figure 4.** Proportional hazards assumption

Primary endpoint was PFS in the modified intent to treat/safety population (mITT/safety)

## A. PFS - Sequential versus MPACT ; mITT population


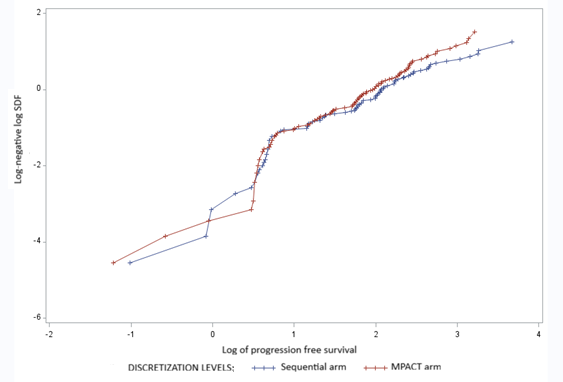


| **Supremum test for the proportional hazards assumption** | | | | |
| --- | --- | --- | --- | --- |
| **Variable** | **Maximum absolute value** | **Replications** | **Seed** | **Pr >  ValAbsMax** |
| **Arm** | 0.8665 | 5000 | 397836803 | 0.3690 |

## B. PFS - NAPOLI versus MPACT ; mITT population


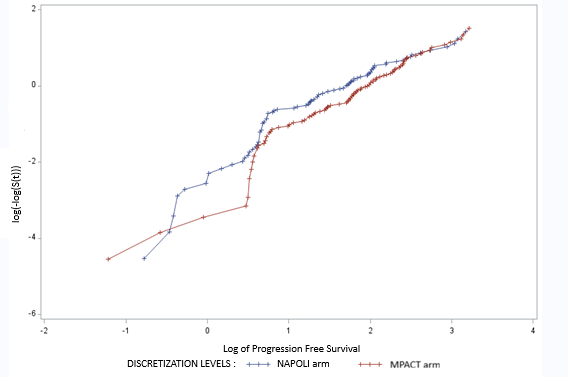


| **Supremum test for the proportional hazards assumption** | | | | |
| --- | --- | --- | --- | --- |
| **Variable** | **Maximum absolute value** | **Replications** | **Seed** | **Pr >  ValAbsMax** |
| **Arm** | 1.0420 | 5000 | 612256752 | 0.1682 |
