# Supplementary material for: Sequential alternation of nal-IRI/5-FU and gemcitabine/nab-paclitaxel versus nal-IRI/5-FU versus gemcitabine/nab-paclitaxel in first-line metastatic pancreatic cancer: results of the randomized phase II PRODIGE 61—FUNGEMAX trial (France)
Source: eClinicalMedicine. 2026 May 29;96:103998. doi: 10.1016/j.eclinm.2026.103998 (PMC13240766; doi:10.1016/j.eclinm.2026.103998)
Supplement: Protocol [file mmc2.docx]

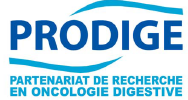

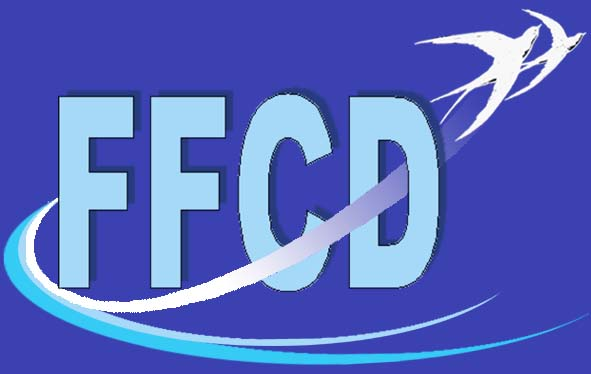
**PRODIGE 61 - (FFCD 1702) – FUNGEMAX**

**Randomized phase II study comparing 5FU/LV+Nal-IRI, gemcitabine+Nab-paclitaxel or a sequential regimen of 2 months 5FU/LV+Nal-IRI followed by two months of gemcitabine+Nab-paclitaxel, in metastatic pancreatic cancer**

**Randomized – comparative – multicenters phase II**

EudraCT no. 2017-004309-41

**An intergroup trial: FFCD – UNICANCER-GI – GERCOR**

**Principal Investigator (FFCD):**

**Prof. Julien Taieb**

HOPITAL EUROPEEN G. POMPIDOU
Service d'Hépato-gastro-entérologie

20 Rue Leblanc
75015 PARIS

Tel. : +33(0)1 56 09 50 42 - Fax : +33(0)1 56 09 35 29

email : [julien.taieb@egp.aphp.fr](mailto:julien.taieb@egp.aphp.fr)

**Co-Principal Investigator (GERCOR):**

**Pr Jean Baptiste Bachet**

Groupe Hospitalier Pitié Salpêtrière

Service d’Hépato-gastroentérologie & Oncologie Digestive

47-83 boulevard de l’Hôpital 75013 Paris ; France

Tel. : +33 (0)1 42 16 10 41– Fax : +33 (0)1 42 16 12 38

email : jean-baptiste.bachet@psl.aphp.fr

**PROTOCOL WRITING COMMITTEE**

Jean-Baptiste Bachet (Paris, La Pitié Salpêtrière), Jérémie Bez (Dijon), Christelle De La Fouchardière (Lyon, Léon Bérard), Claire Gallois (Paris HEGP), Karine Le Malicot (Dijon), Carole Montérymard (Dijon), Julien Taieb (Paris HEGP).

**BIOLOGICAL COMMITTEE**

Pierre Laurent-Puig (Paris HEGP), Julien Taieb (Paris HEGP), Claire Gallois (Paris HEGP), Jean-François Emile (Paris)

**SPONSOR AND DATA CENTER:**

Fédération Francophone de Cancérologie Digestive **(FFCD)**, Faculté de Médecine, 7, Boulevard Jeanne d’Arc, BP 87900, 21079 Dijon Cedex, France

**Executive director:** **Cécile GIRAULT**

Tel: +33 (0)3 80 66 80 13 – Fax: +33 (0)3 80 38 18 41

email: [cecile.girault@u-bourgogne.fr](mailto:cecile.girault@u-bourgogne.fr)

**Statistician:** **Carole MONTERYMARD**

Tel: +33 (0)3 80 39 34 84– Fax: +33 (0)3 80 38 18 41

email: [carole.monterymard@u-bourgogne.fr](mailto:carole.monterymard@u-bourgogne.fr)

**Project manager:** **Daniel GONZALEZ**

Tél. : +33 (0)3 80 39 34 04 – Fax: +33 (0) 3 80 38 18 41

email: [daniel.gonzalez@u-bourgogne.f](mailto:daniel.gonzalez@u-bourgogne.f)r

**Confidential**

This document is the property of the Fédération Francophone de Cancérologie Digestive and may not be transferred, reproduced, published or used, in whole or in part, without the federation's express authorization.

**Table of Contents**

[1. STUDY OBJECTIVES 12](#_Toc227340469)

[1.1. Primary objective 12](#_Toc227340470)

[1.2. Secondary objectives 12](#_Toc227340471)

[2. Patient selection on registration 12](#_Toc227340472)

[2.1. Inclusion criteria 12](#_Toc227340473)

[2.2. Non-inclusion criteria 13](#_Toc227340474)

[3. INCLUSION ASSESSMENT 13](#_Toc227340475)

[4. RANDOMIZATION 14](#_Toc227340476)

[5. STUDY DESIGN 15](#_Toc227340477)

[6. THERAPIES 15](#_Toc227340478)

[6.1. Description, packaging and labeling of Nal-IRI 15](#_Toc227340479)

[6.2. Arm A: Alternatively every 2 months Nal-IRI plus 5FU/LV and Nab-Paclitaxel plus Gemcitabine 15](#_Toc227340480)

[Nal-IRI plus 5-FU/LV 15](#_Toc227340481)

[Nab-Paclitaxel plus Gemcitabine 16](#_Toc227340482)

[6.3. Arm B: Nal-IRI plus LV/5FU 16](#_Toc227340483)

[6.4. Arm C: Nab-Paclitaxel plus Gemcitabine 16](#_Toc227340484)

[7. DOSE ADJUSTMENT BASED ON TOXICITY 16](#_Toc227340485)

[7.1. Criteria that must be met before each subsequent cycle (D1 of each cycle) 17](#_Toc227340486)

[7.2. Dose adjustment based on toxicities observed during the rest period 17](#_Toc227340487)

[7.3. Nal-IRI plus 5FU/LV dose Modification for patients not homozygous for UGT1A1*28 18](#_Toc227340488)

[7.3.1. Hematologic toxicity 18](#_Toc227340489)

[7.4. Nal-IRI plus 5FU/LV dose Modification for patients who are known to be homozygous for UGT1A1*28 19](#_Toc227340490)

[7.5. Nab-Paclitaxel plus Gemcitabine 20](#_Toc227340491)

[7.5.1. Hematologic toxicity 20](#_Toc227340492)

[7.5.2. Non-Hematological toxicity dose modification 20](#_Toc227340493)

[7.6. Dose adaptation related to the occurrence of febrile neutropenia 20](#_Toc227340494)

[7.7. Dose adaptation related to the onset of peripheral neuropathy 21](#_Toc227340495)

[7.8. Dose adjustment in case of occurrence of pulmonary embolism 21](#_Toc227340496)

[7.9. Premedication, concomitant treatments and contraindicated treatments 21](#_Toc227340497)

[7.9.1. Neutropenia 21](#_Toc227340498)

[7.9.2. Gastrointestinal trouble 21](#_Toc227340499)

[7.9.3. Contraindicated treatments (see SmPCs of the protocol's molecular entities) 21](#_Toc227340500)

[8. LOGISTICS OF THE BIOLOGICAL STUDY 22](#_Toc227340501)

[9. PATIENT MONITORING 23](#_Toc227340502)

[9.1. During treatment 23](#_Toc227340503)

[9.1.1. Before each administration of treatment: 23](#_Toc227340504)

[9.1.2. Evaluation every 8 weeks until progression 23](#_Toc227340505)

[9.2. After treatment discontinuation 23](#_Toc227340506)

[9.2.1. **Within 30 days** for evaluating the toxicity of the last treatment: 23](#_Toc227340507)

[9.2.2. After radiological and/or clinical progression, patients will be monitored every 2 to 3 months up to death: 24](#_Toc227340508)

[9.2.3. **After premature discontinuation of treatment other than for progression*** 24](#_Toc227340509)

[Patients will be monitored in the same way every 8 weeks until progression: 24](#_Toc227340510)

[10. SUBSEQUENT TREATMENTS 24](#_Toc227340511)

[11. MANAGEMENT OF SERIOUS ADVERSE EVENTS 24](#_Toc227340512)

[12. STATISTICAL ANALYSIS 26](#_Toc227340513)

[12.1. Endpoints 26](#_Toc227340514)

[12.1.1. Primary efficacy endpoint 26](#_Toc227340515)

[12.1.2. Secondary endpoints 27](#_Toc227340516)

[12.2. Sample size Calculation, statistical hypotheses 27](#_Toc227340517)

[12.3. Statistical analysis plan 27](#_Toc227340518)

[12.3.1. Populations definitions 27](#_Toc227340519)

[12.3.2. Baseline analysis 28](#_Toc227340520)

[12.3.3. Efficacy analysis 28](#_Toc227340521)

[12.3.4. Safety analysis 29](#_Toc227340522)

[13. STUDY COMMITTEES 29](#_Toc227340523)

[13.1. Independent committee 29](#_Toc227340524)

[13.2. Steering committee 29](#_Toc227340525)

[13.3. Medical review 29](#_Toc227340526)

[13.4. Biological research committee 29](#_Toc227340527)

[14. Background information and rationale for the trial 30](#_Toc227340528)

[15. Bibliographical references 32](#_Toc227340529)

[16. ADMINISTRATIVE CONSIDERATIONS 34](#_Toc227340530)

[17. Rules for publication 35](#_Toc227340531)

[18. APPENDICES 35](#_Toc227340532)

[APPENDIX 1: CLINICAL and BIOLOGICAL INFORMED CONSENT 36](#_Toc227340533)

[APPENDIX 2: BIOLOGICAL STUDIES 37](#_Toc227340534)

[APPENDIX 3: QUALITY OF LIFE – QLQ-C30 39](#_Toc227340535)

[Appendix 4: WHO performance status – CALCULATION OF CLEARANCE 40](#_Toc227340536)

[Appendix 5: RECIST criteria, version 1.1 41](#_Toc227340537)

[APPENDIX 6: ASSESSMENT OF TOXICITY (nci-ctc v4.0 ) 43](#_Toc227340538)

[APPENDIX 7: SUMMARIES OF PRODUCT CHARACTERISTICS FOR STUDY PRODUCTS 44](#_Toc227340539)

[APPENDIX 8: Serious Adverse Event Report Form 45](#_Toc227340540)

[APPENDIX 9: RULES FOR PUBLICATION FOR PRODIGE TRIALS 48](#_Toc227340541)

[APPENDIX 10: INSURANCE CERTIFICATE 50](#_Toc227340542)

[APPENDIX 11: APPROVAL OF THE IRB 51](#_Toc227340543)

[Study Protocol Supplementary File 53](#_Toc227340544)

LIST OF ABBREVIATIONS

| ADL | activities of daily living |
| --- | --- |
| AE | adverse event |
| ALP | alkaline phosphatase |
| ALT | alanine aminotransferase (or SGPT, serum glutamic pyruvic transaminase) |
| ANC | absolute neutrophil count |
| ANSM | French National Agency for Medicines and Health Products Safety |
| aPTT | activated partial thromboplastin time |
| AST | aspartate aminotransferase (or SGOT, serum glutamic-oxaloacetic transaminase) |
| AVK | anti-vitamin K |
| BMI | body mass index |
| BP | blood pressure |
| CA 19.9 | cancer antigen 19.9 |
| CBC | complete blood count |
| CEA | carcinoembryonic antigen |
| CI | contraindication |
| CR | complete response |
| CRA | clinical research associate |
| CRF | case report form |
| CT | chemotherapy |
| CT | computed tomography |
| d | day |
| DPD | dihydropyrimidine dehydrogenase |
| EMA | European Medicines Agency |
| FFCD | French Federation of Digestive Oncology |
| G-CSF | granulocyte-colony stimulating factor |
| GGT | gamma-glutamyl transpeptidase |
| Hb | hemoglobin |
| HR | hazard ratio |
| HRQOL | health-related quality of life |
| HT | hypertension |
| IADL | instrumental activities of daily living |
| INR | international normalized ratio |
| IRB | institutional review board |
| ITT | intention to treat |
| IV | intravenous |
| KM | Kaplan Meier |
| LDH | lactate dehydrogenase |
| MRI | magnetic resonance imaging |
| mTNS | modified total neuropathy score |
| N | normal |
| NCI-CTCAE | National Cancer Institute – Common Terminology Criteria for Adverse Events |
| OR | objective response |
| OS | overall survival |
| PFS | progression-free survival |
| PR | partial response |
| PT | prothrombin time |
| Q1-Q3 | quartiles |
| QoL | quality of life |
| RECIST | Response Evaluation Criteria In Solid Tumors |
| SAE | serious adverse event |
| SD | stable disease |
| TAP | thoracic-abdominal-pelvic |
| UD | urine dipstick |
| ULN | upper limit of normal |
| WHO | World Health Organization |

FFCD CONTACTS for the STUDY

| **NAME** | **FUNCTION** | **TEL (+33)** | **FAX (+33)** | **EMAIL** |
| --- | --- | --- | --- | --- |
| **FFCD DATA CENTER** | | | | |
| Cécile Girault | Executive director | (0)3 80 39 33 87 | (0)3 80 38 18 41 | [cecile.girault@u-bourgogne.fr](mailto:cecile.girault@u-bourgogne.fr) |
| Marie Moreau | Clinical Operation leader | (0)3 80 39 34 04 | (0)3 80 38 18 41 | [marie.moreau@u-bourgogne.fr](mailto:marie.moreau@u-bourgogne.fr) |
| **Operational team :** | | | | |
| Daniel Gonzalez | Project manager | (0)3 80 39 34 04 | (0)3 80 38 18 41 | daniel.gonzalez@u-bourgogne.fr |
| Caroline Choine-Pourret | CRA coordinator | (0)4 69 18 19 02 | (0)9 74 44 22 47 | [caroline.choine@u-bourgogne.fr](mailto:carolinechoine.ffcd@sfr.fr) |
| Coralie Devorsine | Assistant project manager | (0)3 80 39 34 86 | (0)3 80 38 18 41 | [coralie.devorsine@u-bourgogne.fr](mailto:coralie.devorsine@u-bourgogne.fr) |
| Mathieu Delgado | Data manager | (0)3 80 39 34 08 | (0)3 80 38 18 41 | mathieu.delgado@u-bourgogne.fr |
| Carole Monterymard | Biostatistician | (0) 3 80 39 34 84 | (0)3 80 38 18 41 | [carole.monterymard@u-bourgogne.fr](mailto:carole.monterymard@u-bourgogne.fr) |

Protocol agreement form

**PRODIGE 61- (FFCD 1702) – FUNGEMAX**

**Randomized phase II study comparing 5FU/LV+Nal-IRI, gemcitabine+Nab-paclitaxel or a sequential regimen of 2 months 5FU/LV+Nal-IRI followed by two months of gemcitabine+ Nab-paclitaxel, in metastatic pancreatic cancer**

EudraCT no. 2017-004309-41

Version 09/04/2018

This version of the protocol has been approved by:


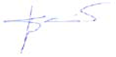
The sponsor: Cécile Girault Date: 09/04/2018 Signature:

The coordinator: Prof. Julien Taieb Date: 09/04/2018 Signature:

I the undersigned, Dr.: ……………….. having taken note of the pre-requisites of this research and of the protocol and its appendices, certify that I will undertake to conduct this trial in compliance with Good Clinical Practice and in accordance with the applicable provisions of the French Public Health Code.

I particularly undertake to:

- comply with the protocol as well as any amendments of which the sponsor notifies me
- supervise the research in the center, train my colleagues in conducting the research, and provide a list of the names of my colleagues
- obtain the status of patients from the vital records office at the time of analysis or if the sponsor so requests if patients are lost to follow-up
- have each patient sign a written consent form once I have familiarized the patient with the information sheet. This I undertake to do before any procedure is performed for the research
- report any serious adverse events or new facts within 24 hours of their being brought to my attention in accordance with the protocol's instructions
- comply with the inclusion and non-inclusion criteria as well as with the start and end dates of the trial
- participate in the biological section of the trial and dispatch the samples according to the guidelines
- fill in all items of the CRF and ensure that data collection is up to standard and that the products are properly managed
- retain the data and documents relating to the trial for 15 years after the trial has ended
- -inform the sponsor of any conflict of interest that may damage my scientific independence within the framework of the research
- immediately inform the sponsor of any legal action, whether amicable or contentious, brought by a person participating in the research or by that person's assignees in which the sponsor may be held accountable
- accept periodic visits from the sponsor's representatives and make all source documents and materials relating to the research available to them so that they may verify the quality of data recorded in the CRF. Accept audits by the sponsor or one of its representatives and/or inspections by the health authorities
- reply by telephone or email to requests for corrections or clarifications concerning the CRF
- accord the FFCD CRA the time necessary for signing forms, answering any questions and carrying out corrective actions

Date: Signature:

**CENTER'S STAMP**:

*Send the original to the FFCD Randomization, Management and Analysis Center – 7 bd Jeanne d’Arc – BP 87900 – 21079 Dijon Cedex, France*

SYNOPSIS

| **Title** | **PRODIGE 61 – FFCD 1702 – FUNGEMAX**  **Randomized phase II study comparing 5-FU/LV+Nal-IRI, gemcitabine+Nab-paclitaxel or a sequential regimen of 2 months 5-FU/LV+Nal-IRI followed by two months of gemcitabine+Nab-paclitaxel, in metastatic pancreatic cancer** |
| --- | --- |
| **Sponsor** | French Federation of Digestive Oncology (FFCD) |
| **Design** | Multicenter randomized phase II comparative open study |
| **Study objectives** | **Primary objective:**   - Compare the progression free survival at 6 months in experimental arms (arm A: Nal-Iri plus 5FU/LV and Nab-Paclitaxel plus Gemcitabine alternatively, arm B: Nal-Iri plus 5FU/LV) VS the reference arm (arm C: Nab-Paclitaxel plus Gemcitabine) according to the RECIST 1.1 criteria   **Secondary objectives:**   - Progression free survival at 6 months (according to central review) - Best objective response rate - Progression free survival (according to the investigator and central review) - Overall survival - Time to treatment failure - Safety - Quality of life (EORTC QLQ-C30) - CA 19-9 and CEA monitoring |
| **Inclusion criteria** | - Histopathologically proven pancreatic adenocarcinoma (on primitive or metastatic lesion) - 18 ≤ age ≤ 75 years - Life expectancy >12 weeks - Performance status WHO < 2 - No prior chemotherapy (adjuvant chemotherapy by gemcitabine +/- capecitabine is allowed if ended at least 12 months before the inclusion) - Pain well controlled before the inclusion of the patient - ANC ≥ 1,500 cells/μL (without the use of hematopoietic growth factors); platelet count ≥ 100,000 cells/μL, hemoglobin ≥ 9 g/dL (blood transfusions is permitted for patients with hemoglobin levels below 9 g/dL) - Adequate hepatic function as evidenced by: Serum total bilirubin within normal range for the institution (Serum bilirubin ≤ 1,5 UNL) Biliary drainage allowed for biliary obstruction. - Albumin levels ≥ 3.0 g/dL - Aspartate aminotransferase (AST) and alanine aminotransferase (ALT) ≤ 2.5 x ULN (≤ 5 x ULN acceptable if liver metastases were present) - Normal renal function test (serum creatinine concentration ≤ 120 µmol/l or creatinine clearance ≥ 50 ml/min) - Normal ECG or ECG without any clinically significant findings - Patient able to understand and sign an informed consent - Females of child-bearing potential are required to test negative for pregnancy at the time of enrollment based on a urine or serum pregnancy test. - Both male and female patients of reproductive potential were required to agree to use a reliable method of birth control, during the study and for 3 months following the last dose of study drug. - Patient affiliated to social security - Regular follow-up possible |
| **Non-inclusion criteria** | - Known brain or bone metastasis (no need of systematic CT scan) - Prior radiation therapy (except if there is at least one measurable target outside irradiation area) - Clinically significant gastrointestinal disorder including hepatic disorders, bleeding, inflammation, occlusion, or diarrhea > Grade 1 - History of any second malignancy in the last 5 years; subjects with prior history of *in-situ* cancer or basal or squamous cell skin cancer are eligible. Subjects with other malignancies are eligible if they had been continuously disease free for at least 5 years. - Severe arterial thromboembolic events (myocardial infarction, unstable angina pectoris, stroke) less than 6 months before inclusion. - NYHA Class III or IV congestive heart failure, ventricular arrhythmias or uncontrolled blood pressure. - Known hypersensitivity to any of the drugs /constituents or non-lipososomal irinotecan - Any other medical or social condition deemed by the investigator to be likely to interfere with a patient’s ability to sign informed consent, cooperate and participate in the study, or interferes with the interpretation of the results. - Use of CYP3A4/UGT1A inducers/inhibitors - Use of strong CYP2C8 inhibitors or inducers, or presence of any other contraindications for nab-paclitaxel or gemcitabine - ILD presence - Pregnant or breast feeding |
| **Study treatment** | **Arm A (experimental arm): Nal-IRI plus 5-FU/LV and Nab-Paclitaxel plus Gemcitabine alternately every two months**  - **Nal-IRI** at 80 mg/m^2^ IV over 90 minutes followed by folinic acid (leucovorin 400 mg/m^2^ IV, or Elvorin 200 mg/m^2^ IV over 30 minutes) then by 5-FU 2400 mg/m^2^ IV over 46-hours, every 2 weeks.  According the recommendations of the transparency commission, the systematic determination of UGT1A1*28 polymorphism is recommended before the first cure of Nal-IRI.  Patients known to be homozygous for UGT1A1*28 allele will receive the first cycle of therapy at a reduced Nal-IRI dose of 60 mg/m². If the patient does not experience any drug related toxicity after the first administration of Nal-IRI, the dose is permitted to be increased to 80 mg/m^2^ from cycle 2 onwards.  -**Nab-Paclitaxel + Gemcitabine** (6 injections, one injection three weeks out of four; so ≈ 2 months per cycle)  Day 1 (D1): Nab-Paclitaxel plus Gemcitabine at the dose of :  -Gemcitabine: 1000 mg/m² in 500 ml normal saline infusion at a fixed dose rate of 10 mg/m²/min (*i.e.* 100 min).  -Nab-Paclitaxel: 125 mg/m^2^  This treatment is administered at D1, D8, D15 and at D29, D36, D43.  This therapeutic sequence (5-FU/LV plus Nal-IRI followed by Nab-Paclitaxel + Gemcitabine) will be repeated until disease progression or unacceptable toxicity.  The response or the progression with each treatment will be censored. In case of progression or limiting toxicity with one of these two treatments, the other one will be continued until tumoral progression, limiting toxicity or patient’s refusal.  Clinical, biological and morphological assessments will be done every 2 months  **Arm B (experimental arm): Nal-IRI plus 5-FU/LV**  **Nal-IRI** at 80 mg/m^2^ IV over 90 minutes followed by folinic acid (leucovorin 400 mg/m^2^ IV, or Elvorin 200 mg/m^2^ IV over 30 minutes) then by 5-FU 2400 mg/m^2^ IV over 46-hours, every 2 weeks.  According the recommendations of the transparency commission, the systematic determination of UGT1A1*28 polymorphism is recommended before the first cure of Nal-IRI.  Patients known to be homozygous for UGT1A1*28 allele will receive the first cycle of therapy at a reduced Nal-IRI dose of 60 mg/m^2^. If the patient does not experience any drug related toxicity after the first administration of Nal-IRI, the dose is permitted to be increased to 80 mg/m^2^ from cycle 2 onwards.  This treatment will be continued until disease progression (clinical and/or radiological), limiting toxicity or patient’s refusal and evaluation will be performed every 2 months.  **Arm C (reference arm): Nab-Paclitaxel plus Gemcitabine**  **Nab-Paclitaxel + Gemcitabine** (6 courses, one course three weeks out of four; so ≈ 2 months per cycle)  Day 1 (D1): Nab-Paclitaxel + Gemcitabine at the dose of :  -Gemcitabine: 1000 mg/m² in 500 ml normal saline infusion at a fixed dose rate of 10 mg/m²/min (*i.e.* 100 min).  -Nab-Paclitaxel: 125 mg/m^2^  This treatment is administered at D1, D8, D15 and at D29, D36, D43.  This treatment will be continued until disease progression (clinical and/or radiological), limiting toxicity or patient’s refusal and evaluation will be performed every 2 months. |
| **Randomization** | Randomization (1:1:1) of the patient will be done according to a minimization technique and will be stratified according to the following stratification factors:   - Center - WHO/PS 0 versus 1 - 1 versus >1 metastatic sites |
| **Sample size calculation** | The hypotheses used to calculate the sample size are:   - H0: The rate of patients alive without progression at 6 months is not different between arms. - H1: The rate of patients alive without progression at 6 months in experimental arms is different from the rate in standard arm **(same hypothesis for the 2 experimental arms**).   We expected a difference of 15% at 6 months in favor of experimental arms vs standard arm (from 40% to 55%): HR=0.65  With a two-sided α risk of 5% and a power of 80%, it will be necessary to observe 170 events (progression or death), (estimated sample sizes for two-sample comparison of survivor functions Log-rank test, Schoenfeld method).  Taking into account an assumption of 2 years of recruitment, a patient's follow-up of 3 years and with a percentage of patients lost to follow-up of 10%, it will be necessary to randomize 190 patients (95 patients in each compared arm meaning **285 patients overall = 95 patients*3 arms**). |
| **Statistical analysis (generality)** | Baseline characteristics will be described using descriptive statistics as percentages (with 95% CI) for categorical and ordinal variables and mean (with standard deviation), median (with interval Inter-quartiles and Min-max) for continuous variables. The results will be presented by treatment group and on the overall population.  Comparisons by treatment arm will be performed for the quantitative variables, using a Student or Wilcoxon test (according to the distribution of the variables) and for qualitative variables, using a Chi² test or a Fisher exact test.  For the primary analysis, the statistical method to demonstrate the superiority of experimental arms against standard arm will be the step-down procedure.  Progression free survival and overall survival (OS) will be measured from the date of randomization and will be estimated using Kaplan-Meier method. The median times and rates at different temporalities shall be described as well as their confidence intervals at 95%. Treatment arms comparisons will be done using log-rank test.  Toxicities, doses received will be described by treatment arms.  A more detailed Statistical Analysis Plan (SAP) will be written before the database lock. |
| **Ancillary study** | Blood and tissue will be collected prospectively at the biological resource center of FFCD for future translational projects There will be at least analysis of circulating tumor DNA before treatment to look for predictive factors. |
| **Number of patients** | 285 patients |
| **Duration of inclusion and length of participation for each patient** | Theoretical rate of inclusion: 10 patients per month  Number of centers: 40  Theoretical start of inclusion: Q3 2018  Theoretical end of inclusion: Q4 2021  End of the trial (primary and secondary endpoint analysis): Q4 2024 |

EXAMINATION AND FOLLOW-UP SCHEDULE

|  | **BEFORE TREATMENT** | **DURING TREATMENT** | | **AFTER DISCONTINUATION OF THE TREATMENT for radiological progression (failure of strategies)** |
| --- | --- | --- | --- | --- |
|  | **During the 14 days preceding the start of treatment** | **Before each course of treatment** | **Every 8 weeks regardless of the arm** | **Every 2 to 3 months up to death** |
| **Clinical and biological informed consent** | X |  |  |  |
| **CLINICAL EXAMINATION** | | | | |
| Weight, body area | X | X | X | X |
| Size | X |  |  |  |
| General condition WHO | X | X | X | X |
| Evaluation of toxicities NCI-CTC Version 4.0 (appendix 6) |  | X | X | X (persitant tox) |
| Evaluation of peripheral neuropathies (NCI-CTC v4.0) (appendix 6) | X | X | X | X |
| Evaluation of neutotoxicites mTNS simplified (appendix 6) | X |  | X | X (2 months after the end of treatment) |
| QLQ-C30 (appendix 3) | X |  | X | X |
| Pain evaluation (EVA) | X |  | X |  |
| **Biological TESTS** | | | | |
| Biological tests | X* | X*** | X* |  |
| Pregnancy test | X |  | X and 30 days after treatment end |  |
| UGT1A1*28 polymorphism and DPD polymorphism | X |  |  |  |
| CA19 -9 et CEA markers | X |  | X |  |
| **PARACLINICAL REVIEWS** | | | | |
| Thoraco-abdominal-pelvic CT scan or MRI | X** |  | X | X |
| ECG | X** |  |  |  |
| **ANCILARY BIOLOGICAL STUDY** | | | | |
| Blood samples (2 tubes / sample) | X |  | X**** |  |
| Biopsies or tumor block, fixed in paraffin | X |  |  |  |
| **FUTURE LINES** | | | | |
| Start and end dates of treatment and the type of treatment of the subsequent lines will be completed in the CRF |  |  |  | X |

*: NFS, platelets, PT, sodium, potassium, calcium, magnesium, bilirubin (total and conjugated), GGT, ALT, AST, alkaline phosphatase, LDH, serum creatinine, creatinine clearance (MDRD - Appendix 4), protein, albumin, pre-albumin, CRP, urea, INT, aPTT

**: Within 3 weeks prior to randomization

***: NFS, platelets bilirubin (total and conjugated) serum creatinine, MDRD clearance, sodium, potassium

****: Blood sample at 4 weeks (after the first cycle of Nab paclitaxel + gemcitabine in arm C and before the third injection of Nal-IRI plus 5FU/LV in arm A and B)

Send an anonymized copy of the imaging on CD ROM to the FFCD, 7 bd Jeanne d'Arc, BP 87900, 21079 Dijon Cedex (centralised review for secondary endpoint)

# STUDY OBJECTIVES

## 1.1. Primary objective

Compare the progression free survival at 6 months in experimental arms (arm A: Nal-Iri plus 5FU/LV and Nab-Paclitaxel plus Gemcitabine alternatively, arm B: Nal-Iri plus 5FU/LV) VS the reference arm (arm C: Nab-Paclitaxel plus Gemcitabine) according to the RECIST 1.1 criteria

## 1.2. Secondary objectives

- Progression free survival at 6 months (according to central review)
- Best objective response rate
- Progression free survival (according to the investigator and central review)
- Overall survival
- Time to treatment failure
- Safety
- Quality of life (EORTC QLQ-C30)
- CA 19-9 and CEA monitoring

**Ancillary biological studies (optional)**

Biological samples, blood and tumors will be collected in order to determine future predictive/prognostic biomarkers in non-pretreated metastatic pancreatic cancer patients. It will include at least: analysis circulating tumor DNA, performed before treatment to investigate somatic (tumor-related molecular alterations) molecular factors with prognostic or predictive value.

# Patient selection on registration

## 2.1. Inclusion criteria

- Histopathologically proven pancreatic adenocarcinoma (on primitive or metastatic lesion)
- 18 ≤ age ≤ 75 years
- Life expectancy >12 weeks
- Performance status WHO < 2
- No prior chemotherapy (adjuvant chemotherapy by gemcitabine +/- capecitabine is allowed if ended at least 12 months before the inclusion)
- Pain well controlled before the inclusion of the patient
- ANC ≥ 1,500 cells/μL (without the use of hematopoietic growth factors); platelet count ≥ 100,000 cells/μL, hemoglobin ≥ 9 g/dL (blood transfusions is permitted for patients with hemoglobin levels below 9 g/dL)
- Adequate hepatic function as evidenced by: Serum total bilirubin within normal range for the institution (Serum bilirubin ≤ 1,5 UNL) Biliary drainage allowed for biliary obstruction.
- Albumin levels ≥ 3.0 g/dL
- Aspartate aminotransferase (AST) and alanine aminotransferase (ALT) ≤ 2.5 x ULN (≤ 5 x ULN acceptable if liver metastases were present)
- Normal renal function test (serum creatinine concentration ≤ 120 µmol/l or creatinine clearance ≥ 50 ml/min)
- Normal ECG or ECG without any clinically significant findings
- Patient able to understand and sign an informed consent
- Females of child-bearing potential are required to test negative for pregnancy at the time of enrollment based on a urine or serum pregnancy test.
- Both male and female patients of reproductive potential were required to agree to use a reliable method of birth control, during the study and for 3 months following the last dose of study drug.
- Patient affiliated to social security
- Regular follow-up possible

## 2.2. Non-inclusion criteria

- Known brain or bone metastasis (no need of systematic CT scan)
- Prior radiation therapy (except if there is at least one measurable target outside irradiation area)
- Clinically significant gastrointestinal disorder including hepatic disorders, bleeding, inflammation, occlusion, or diarrhea > Grade 1
- History of any second malignancy in the last 5 years; subjects with prior history of *in-situ* cancer or basal or squamous cell skin cancer are eligible. Subjects with other malignancies are eligible if they had been continuously disease free for at least 5 years.
- Severe arterial thromboembolic events (myocardial infarction, unstable angina pectoris, stroke) less than 6 months before inclusion.
- NYHA Class III or IV congestive heart failure, ventricular arrhythmias or uncontrolled blood pressure.
- Known hypersensitivity to any of the drugs /constituents or non-lipososomal irinotecan
- Any other medical or social condition deemed by the investigator to be likely to interfere with a patient’s ability to sign informed consent, cooperate and participate in the study, or interferes with the interpretation of the results.
- Use of CYP3A4/UGT1A inducers/inhibitors
- Use of strong CYP2C8 inhibitors or inducers, or presence of any other contraindications for nab-paclitaxel or gemcitabine
- ILD presence
- Pregnant or breast feeding

# INCLUSION ASSESSMENT

The inclusion assessment must be conducted during the 14 days before randomization. This does not apply to morphological examinations, which may be conducted during the 3 weeks before randomization.

Clinical examination:

- Measurement of weight (including an evaluation of weight loss %), height and body surface area
- WHO performance status (Appendix 4)
- Evaluation of peripheral neurological symptoms using mTNS simplified scale and NCI-CTC v4.0 (Appendix 6)
- Evaluation of pain (EVA): Ask to the patient to define on a scale from 0 to 10 the degree of his pain. 0 being “no pain” and 10 being “maximal imaginable pain”.

Quality-of-life questionnaire

To be completed by the patient before randomization (same day or within 14 days before randomization but in any case at least before the first course of treatment.)

Laboratory assessment at least 14 days before randomization comprising:

- FBC, platelets, PT
- Liver panel comprising ALP, AST, ALT, total and conjugated bilirubin, and LDH
- Creatinine and creatinine clearance (MDRD - Appendix 4)
- Blood protein, albumin, prealbumin and CRP (Glasgow pronostic score)
- Serum electrolytes (sodium, potassium, calcium, magnesium),
- Markers: CA 19-9, CEA
- Pregnancy test if women of childbearing age
- Recommendation of the UGT1A1*28 polymorphism and DPD polymorphism determination determination according to respectively, the transparency commission and French health authorities (ANSM).

Morphological examinations and ECG **within 3 weeks prior to randomization:**

- Thoraco-abdominal-pelvic scan (CT scan TAP or abdominal MRI + thoracic TDM scan without injection if injected scan contraindicated)
- ECG

**If a patient is participating in the ancillary biological study of circulating tumor DNA**

- Two cell- free DNA tubes of blood are taken before the first treatment cycle. The rational and logistics of this study are described in Appendix 2 and Chapter 8)

# RANDOMIZATION

After signing the consent form and validating the results of the initial baseline assessment, eligible patients will be randomized at the FFCD data center, **CRGA [Centre de Randomisation – Gestion – Analyse].**

The investigator will fax the completed and signed randomization form to the FFCD data center:

**Monday to Friday from 9 am to 6pm**

**Fax: + 33 (0)3 80 38 18 41**/**Tel: + 33 (0)3 80 66 80 13**

A randomization confirmation will be send back to the investigator and to the pharmacist with the patient registration number and the arm allocated by the randomization.

After randomization, treatment should begin as soon as possible and within a maximum period of 10 days.

**Stratification**

The randomization (1:1:1) of the patients will be done according to minimization technic according to the following stratification factors:

- Center
- WHO/PS 0 versus 1
- 1 versus >1 metastatic sites

# STUDY DESIGN


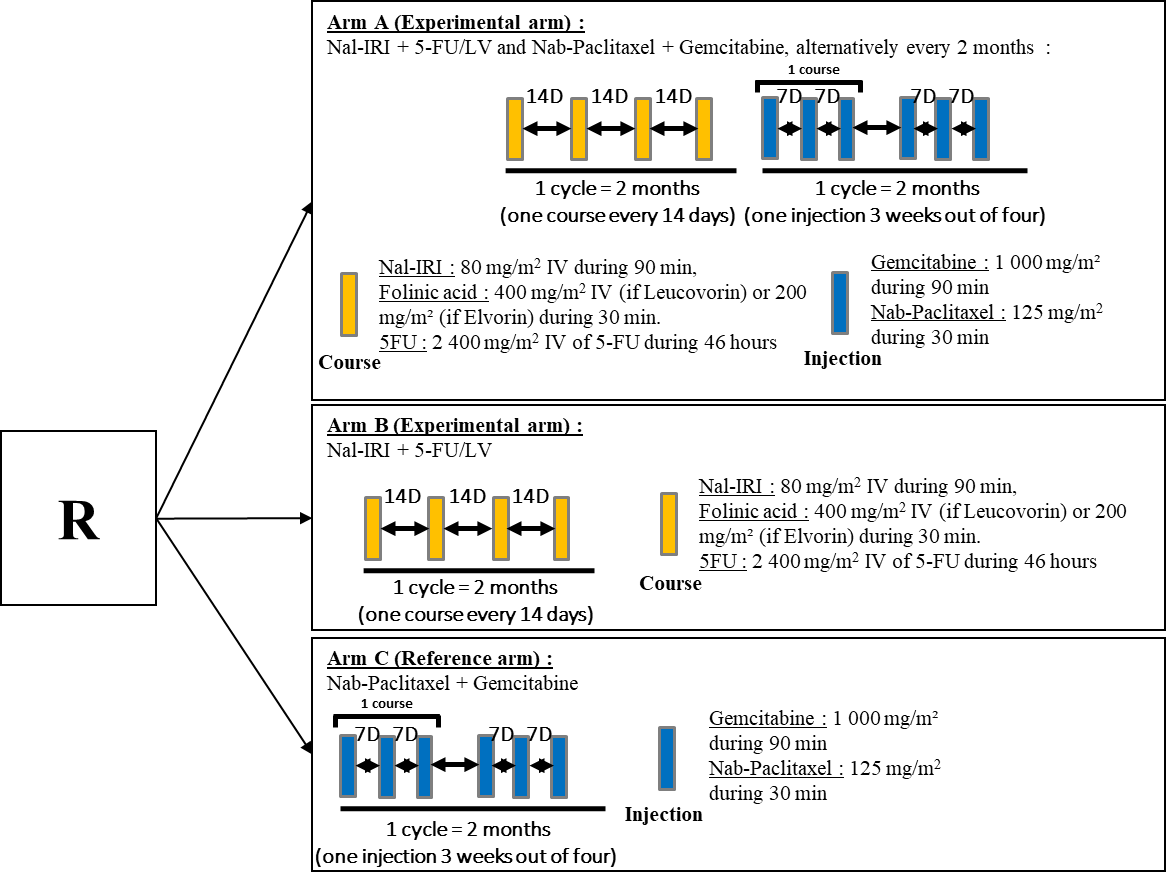


**In case of arm A:** In case of progression, limiting toxicity for one of the two treatment, it is recommended (excepted contraindication or refusal) to continue the other treatment until progression, limiting toxicity or patient refusal.

# THERAPIES

## 6.1. Description, packaging and labeling of Nal-IRI

Nal-IRI (irinotecan liposome injection) is irinotecan in the form of the sucrosofate salt, encapsulated in liposomes for intravenous infusion.

It will be supplied in sterils, single-use vials containing 10 mL or Nal-IRI at a concentration of 5 mg/mL.

Nal-IRI must be stored refrigerated at 2 to 8 °C, with protection from light.

Light protection is not required during infusion. Nal-IRI must be diluted prior to administration. The diluted solution is physically and chemically stable for 4 hours at room temperature (15-25°C). Because of possible microbial contamination during dilution, it is advisable to use the diluted solution within 24 hours if refrigerated (2-8°C), and within 4 hours if kept at room temperature (15-25°C).

Twelve vials of Nal-IRI will be packaged in a cardboard container. The individual vials, as well as the outside of the cardboard container, will be labeled in accordance with local regulatory requirements.

## 6.2. Arm A: Alternatively every 2 months Nal-IRI plus 5FU/LV and Nab-Paclitaxel plus Gemcitabine

### Nal-IRI plus 5-FU/LV

One course every 14 days (D1, D15, D29…)

All patients must be premedicated prior to Nal-IRI infusion, 5-FU/LV infusion with standard doses of standard corticïdes (methylprednisolone or dexamethasone) and a 5-HT3 antagonist (Granisetron, …), or equivalent other antiemetic according to standard institutional practices for irinotecan and 5-FU, or the Summary of Product Characteristics (SmPC) for sites.

Atropine may be prescribed prophylactically for patients who experienced acute cholinergic symptoms in the previous cycles.

Nal-IRI will be administrated first, followed by Leucovorin or Elvorin and then followed by 5-FU.

**Nal-IRI**: 80 mg/m² IV over 90 minutes

**Folinic acid:** Leucovorin**:** 400 mg/m² IV over 30 minutes or Elvorin at 200 mg/m² IV over 30 minutes

**5-FU:** 2400 mg/m² over 46-hours every 14 days

### Nab-Paclitaxel plus Gemcitabine

3 weeks out of 4, meaning one injection at D1, D8, D15 then D29, D36 and D43 (2 months of treatment)

**Nab-paclitaxel:** 125 mg/m² of Nab-paclitaxel in perfusion over 30 minutes - at D1, D8, D15 then D29, D36 and D43

**Gemcitabine:** 1000 mg/m² in perfusion over 30 minutes immediately after the end of Nab-paclitaxel perfusion – at D1, D8, D15 then D29, D36 and D43

In case of progression, limiting toxicity for one of the two treatment, it is recommended (excepted contraindication or refusal) to continue the other treatment until progression, limiting toxicity or patient refusal.

## 6.3. Arm B: Nal-IRI plus LV/5FU

One course every 14 days meaning one course at D1, D15, D29, D43, D57 for two months of treatment

All patients must be premedicated prior to Nal-IRI infusion, 5-FU/LV infusion with standard doses of standard corticïdes (methylprednisolone or dexamethasone) and a 5-HT3 antagonist (Granisetron, …), or equivalent other antiemetic according to standard institutional practices for irinotecan and 5-FU, or the Summary of Product Characteristics (SmPC) for sites.

Nal-IRI will be administrated first, followed by Leucovorin or Elvorin and then followed by 5-FU.

**Nal-IRI**: 80 mg/m² IV over 90 minutes

**Folinic acid:** Leucovorin**:** 400 mg/m² IV over 30 minutes or Elvorin at 200 mg/m² IV over 30 minutes

**5-FU:** 2400 mg/m² over 46-hours every 14 days

## 6.4. Arm C: Nab-Paclitaxel plus Gemcitabine

3 weeks out of 4, meaning one injection at D1, D8, D15 then D29, D36 and D43 (2 months of treatment)

**Nab-paclitaxel:** 125 mg/m² of nab-paclitaxel in perfusion over 30 minutes - at D1, D8, D15 then D29, D36 and D43

**Gemcitabine:** 1000 mg/m² in perfusion over 30 minutes immediately after the end of Nab-paclitaxel perfusion – at D1, D8, D15 then D29, D36 and D43

# DOSE ADJUSTMENT BASED ON TOXICITY

The toxicities requiring dose adjustments will all be evaluated according to the scale NCI-CTCAE v4.0 (Appendix 6)

## 7.1. Criteria that must be met before each subsequent cycle (D1 of each cycle)

Criteria that must to be met before each subsequent cycle of treatment:

- WHO < 2
- ANC ≥ 1500 /mm^3^
- WBC ≥ 3500/mm^3^
- Plated count ≥ 100 000/mm^3^
- Diarrhea ≤ grade 1

As long as these criteria have not been met, symptomatic treatment should be optimised and an FBC will be conducted every 7 days until obtaining the required figures. The treatment can then be carried out.

In addition to these criteria:

Gemcitabine may be administered if the transaminases are ≤ 5 times the normal.

As long as these criteria are not met, the treatment should be postponed for a week.

If over 21 days of treatment deferral after the last administration, the patient will stop the treatment under study and will continue to be monitored as part of the protocol.

In arm C, the indication of primary prophylaxis is at the discretion of the investigator (It is not mandatory but strongly recommended)

## 7.2. Dose adjustment based on toxicities observed during the rest period

Dosage adjustments are needed depending on the maximum grade of toxicity observed between courses of treatment.

The treatments will only be begun when the criteria required before implementation of any new treatment is obtained (see paragraph 7.1).

The occurrence of grade 4 toxicity (excluding hematologic toxicities or other manageable toxicity) shall require the permanent discontinuation of the study treatments unless the investigator considers that there is an interest for the patient to continue with the rest of the treatment when the alleged responsibility of the toxicity observed is not deducted. The recourse treatments will be at the discretion of the investigator. In all cases, the patient will continue to be monitored as part of the protocol according to the protocol pace.

**Arm A, B and C Allergic reactions during Nab-paclitaxel infusion and Nal-IRI plus 5-FU/LV infusion**

| Grade NCI V4.0 | Description | Action |
| --- | --- | --- |
| Grade 1 | Hot flush or transient rash; drug-induced fever < 38 °C; Intervention not indicated | Slow infusion rate by 50%.  Monitor patient every 15 minutes for worsening of condition  Future infusions may be administered at a reduced rate (e.g. over 120 minutes for Nal-IRI), at the discretion of the Investigator. |
| Grade 2 | Requires treatment or a halting of the infusion; responds quickly to a symptomatic treatment (e.g. antihistamines, non-steroidal anti-inflammatory drugs or morphine-containing drugs); prophylactic treatment required for less than 24 hours | Discontinue infusion  H1-antihistamine ± IV corticosteroids  Resume infusion at 50% of the prior rate once infusion reaction has resolved  Monitor patient every 15 minutes for worsening of condition  H1-antihistamine and IV corticosteroids  Future infusions may be administered at a reduced rate (e.g. over 120 minutes for Nal-IRI), at the discretion of the Investigator. |
| Grade 3 | Prolonged (*e.g.* does not respond quickly to symptomatic treatment and/or a brief halting of the infusion); symptoms reappear after initial improvement; requires hospitalization because of clinical repercussions (*e.g.* kidney failure or pulmonary infiltrates) | Discontinue infusion and disconnect infusion tubing from patient  H1-antihistamine and IV corticosteroids. If necessary, bronchodilators and other medically indicated treatments.  No further treatment by Nal-IRI or Nab-Paclitaxel will be permitted |
| Grade 4 | Life-threatening; requires emergency treatment | Discontinue infusion and disconnect infusion tubing from patient  Administer epinephrine, bronchodilators or oxygen as indicated for bronchospasm  H1-antihistamine and IV corticosteroids  Consider hospital admission for observation  No further treatment will be permitted |

For patients who experience a second grade 1 infusion reaction, administer H1-antihistamine and IV corticosteroids. All subsequent infusions should be premedicated with H1-antihistamine and IV corticosteroids.

## 7.3. Nal-IRI plus 5FU/LV dose Modification for patients not homozygous for UGT1A1*28

### 7.3.1. Hematologic toxicity

| **Worst toxicity by CTCAE Grade** | **Nal-IRI** | **5-FU** |
| --- | --- | --- |
| Neutropenia grade 2 (ANC < 1500 – 1000/mm^3^) | 100% of previous dose | 100% of previous dose |
| Neutropenia grade 3 or 4 (ANC ≤ 1000/mm^3^) or febrile neutropenia^*^ | 1^st^ occurrence: reduce dose to 60 mg/m²  2^nd^ occurrence: Reduce dose to 50 mg/m² | 25% of dose reduction |
| Thrombocytopenia ≥ Grade 2 ( platelets ≤ 75 000/mm^3^-50 000/mm^3^ OR grade 3-4: platelets < 50 000/mm^3^ ) | If grade 2: 100% of previous dose  If grade 3: 1^st^ occurrence: reduce dose to 60 mg/m²  2^nd^ occurrence: Reduce dose to 50 mg/m² | If grade 2: 100% of previous dose  If ≥ grade 3: 1^st^ occurrence: reduce dose by 25%  2de occurrence: Reduce dose another 25% (50% of original dose) |
| Other hematologic toxicities not specifically listed above | If grade ≤ 2: 100 % of previous dose  If grade ≥3:  at 1^st^ occurrence, reduce dose to 60 mg/m²  at 2^nd^ occurrence, reduce dose to 50 mg/m² | If grade ≤ 2: 100 % of previous dose  If grade ≥ 3:  at 1^st^ occurrence, reduce dose by 25%  at 2^nd^ occurrence, reduce dose another 25% (50% original dose) |

^*^: Consider the use of G-CSF for patients who experience ≥ grade 3 neutropenia or febrile neutropenia

**Non-Hematological toxicity dose modification other than Asthenia and Grade 3 anorexia^a^**

| **Worst toxicity by CTCAE Grade** | **Nal-IRI** | **5-FU** |
| --- | --- | --- |
| Grade 1 or 2, including diarrhea^b^ | 100 % of previous dose | 100% of previous dose, except for Grade 2 Hand foot syndrome, Grade 2 cardiac toxicity, or any grade neurocerebellar toxicity |
| Grade 3 or 4, including diarrhea^c^ (except nausea and vomiting) | 1^st^ occurrence: Reduce dose to 60 mg/m²  2^nd^ occurrence: Reduce dos to 50 ng/m² | 1^st^ occurrence: Reduce dose by 25%  2^nd^ occurrence: Reduce dose another 25% (50% of original dose)^d^  Except for grade 3 or 4 hand foot syndrome |
| Grade 3 or 4 nausea and/or vomiting despite anti-emetic therapy | Optimize anti-emetic therapy AND reduce dose to 60 mg/m² ; if the patient is already receiving 60 mg/m² reduce dose to 50 mg/m²^, e^ | Optimize anti-emetic therapy AND reduce dose by 25% ; if the patient is already receiving a reduced dose, reduce dose an additional 25%^e^ |
| Grade 2 hand foot syndrome | 100 of previous dose | 1^st^ occurrence: Reduce dose by 25%  2^nd^ occurrence: Reduce dose another 25% (50% of original dose)^d^ |
| Grade 3 or 4 hand foot syndrome | 1^st^ occurrence: Reduce dose to 60 mg/m²  2^nd^ occurrence: Reduce dose to 50mg/m² | Discontinue therapy |
| Any grade neurocerebellar or ≥ grade 2 cardiac toxicity | No dose modifications required | Discontinue therapy |

^a^: Asthenia and grade 3 Anorexia do not require dose modification

^b^: Grade 1 diarrhea: 2-3 stools/day before pretreatment, Grade 2 diarrhea: 4-6 stools/day before pretreatment

^c^: Grade 3 diarrhea: 7-9 stools/day before pretreatment; Grade 4 diarrhea:> 10 stools/day before pretreatment

^d^: Any toxicity ≥ Grade 2, except anemia and alopecia, can justify a dose reduction if medically indicated

e: Patients who require more than 2 dose reductions must be discontinue therapy

## 7.4. Nal-IRI plus 5FU/LV dose Modification for patients who are known to be homozygous for UGT1A1*28

| **Worst toxicity by CTCAE Grade** | **Nal-IRI / 5-FU adjustment (without previous increase to 80 mg/m²)** | |
| --- | --- | --- |
| Adverse reactions grade 3 or 4**^1^** | A new cycle of therapy should not begin until adverse events resolves to grade ≤ 1 | |
|  | First occurrence | Reduce Nal-IRI dose to 50 mg/m2  5-FU dose modification as in paragraph 7.3.1 |
|  | Second occurrence | Reduce Nal-IRI dose to 40 mg/m2  5-FU dose modification as in paragraph 7.3.1 |
|  | Third occurence | Discontinue treatment |

**^1^** Excludes asthenia and anorexia; asthenia and grade 3 anorexia do not require dose adjustment.

For Patients known to be homozygous for UGT1A1*28 allele and randomized in arm Nal- IRI plus 5-FU/LV, a reduced dose of Nal-IRI of 60 mg/m2 should be considered.

If the patient did not experience any drug related toxicity after the first administration of Nal-IRI, a dose increase of Nal-IRI to 80 mg/m² should be considered if tolerated in subsequent cycles.

## 7.5. Nab-Paclitaxel plus Gemcitabine

### 7.5.1. Hematologic toxicity

| **Toxicity/Grade CTCAE v4.0** | **1** | **2** | **3-4** |
| --- | --- | --- | --- |
| Anaemia | No modification | No modification, transfusion support to be discussed | No modification,  transfusion support mandatory |
| Neutropenia,  Thrombopenia | No modification | No modification if administration within a period of 14D^1^  Administration of G-CSF^4^ to be discussed | - Gemcitabine 800 mg/m²  - Nab-Paclitaxel^2^100 mg/m²  If neutropenia, administration of G-CSF^3^ required. |

^1^: In case of non-recovery of toxicities at D14: gastrointestinal (grade > 2 diarrhoea persistence) or haematological (persistence of grade > 2 for ANC or grade> 1 at D21 for platelets), we will carry out reductions in doses recommended for grade 3.

^2^: In case of persistent grade 3 neutropenia despite dosage adjustment and administration of G-CSF, Nab-Paclitaxel will be reduced to 75 mg/m².

If, despite these dose adaptations, at D14 there is a persistence of grade > 2 for ANC or grade > 1 for platelets, the patient will ended protocolar treatment and patient will be followed up according to the arrest treatment paragraph describe the protocol.

^3^: G-CSF: WBC growth factors

### 7.5.2. Non-Hematological toxicity dose modification

| **Toxicity/Grade CTCAE v4.0** | **1** | **2** | **3** |
| --- | --- | --- | --- |
| Diarrhea | No modification | No modification if administration within a period of 14 D ^1^ | - Gemcitabine 800 mg/m²  - Nab-Paclitaxel 100 mg/m² |
| Other^2^ | No modification | No modification | - Gemcitabine 800 mg/m²  - Nab-Paclitaxel 100 mg/m² |

^1^: In case of non-recovery of toxicities at D14: gastrointestinal (grade > 2 diarrhoea persistence) or haematological (persistence of grade > 2 for ANC or grade> 1 at D21 for platelets), we will carry out reductions in doses recommended for grade 3.

^2^: Except: alopecia, cholinergic syndrome and nausea/vomiting in the absence of adequate treatment

## 7.6. Dose adaptation related to the occurrence of febrile neutropenia

The treatment should be discontinued until resolution of fever and recovery of an ANC rate of > 1500/mm^3^. The treatment will be resumed according to the recommended dosage reductions for grade 3, on condition of administration of G-CSF.

If febrile neutropenia occurs during treatment with Gemcitabine + Nab-Paclitaxel, while doses have already been reduced, Nab-Paclitaxel will be reduced to 75 mg/m².

## 7.7. Dose adaptation related to the onset of peripheral neuropathy

Dose adjustment will depend on the scale of peripheral neurological toxicities CTCAE v4 (Appendix 6).

Gemcitabine will be continued with no dose modification.

In the event of grade 1 or 2 peripheral neuropathy, dose adjustment is not recommended.

In the event of ≥ grade 3 peripheral neuropathy, Nab-Paclitaxel must be suspended until recovery from ≤ grade 1 and then continued at 100 mg/m².

If grade 3 neuropathy occurs when Nab-Paclitaxel doses are already reduced, it should be suspended until recovery of grade ≤ 1 and then continued at 75 mg/m².

## 7.8. Dose adjustment in case of occurrence of pulmonary embolism

Clinically asymptomatic or mild pulmonary embolism can be treated with low molecular weight heparin without suspension of treatment.

In patients with moderate to severe pulmonary embolism (grade 3-4), treatment should be permanently discontinued and the patient should stop the protocol treatment.

## 7.9. Premedication, concomitant treatments and contraindicated treatments

### 7.9.1. Neutropenia

The treatment considered necessary for patient well-being can be administered at the discretion of the investigator.

The subcutaneous administration of growth factor is permissible.

According to EORTC 2010 recommendations, the risk of febrile neutropenia should be assessed before each chemotherapy cycle. The validated risk factors are: age > 65 years, a history of febrile neutropenia and advanced disease.

In the event of severe neutropenia, i.e. grade 3-4, patients are at high risk of febrile neutropenia and infection notably in the event of concomitant diarrhoea. If these symptoms occur, dosage adjustments are planned for the next treatment and the prescription of hematopoietic growth factors should be considered.

In arm C (Nab paclitaxel plus gemcitabine), the indication of primary prophylaxis is at the discretion of the investigator (It is not mandatory but strongly recommended).

Pain assessment (EVA) should be reported in the case report. Data should be collected from the inclusion visit and throughout the treatment. For pain assessment, the patient expresses their degree of pain on a scale of 0-10 (0 being "no pain" and 10 being "worst pain imaginable".

### 7.9.2. Gastrointestinal trouble

Utilization of atropine is recommended in primary or secondary prophylaxis for patients who suffered from acute cholinergic syndroma during the previous courses.

In case of rapid onset diarrhoea, an atropine treatment of 0.25-1 mg will be performed (if no contraindications). In case of later diarrhoea (more than 24 hours after the administration), a loperamide treatment and octreotide treatment (in case of loperamide inefficacy) will be performed.

### 7.9.3. Contraindicated treatments (see SmPCs of the protocol's molecular entities)

Nal-IRI: association with CYP3A4 inducers/inhibitors and potent UGT1A1 inhibitors (cf. Appendix 7).

Irinotecan: association with St. John's wort, yellow fever vaccine.

5-FU: yellow fever vaccine, attenuated live vaccine, prophylactic phenytoin. When combined with warfarin more frequent monitoring of INR

Gemcitabine: Hypersensitivity to the active substance or to any of the excipients.

Nab-paclitaxel:  No interaction study has been performed, caution is required when using inhibitors or enzyme inducing drugs. Hypersensitivity to the active substance or to any of the excipients for Nal-IRI-5-FU/LV and paclitaxel.

# LOGISTICS OF THE BIOLOGICAL STUDY

For patients who signed the biological informed consent, the details of the biological ancillary study (circulating DNA and tumor sample) is in Appendix 2 of this protocol.

***Samples needed***

**- Blood tubes will be sampled:**

**-** 2 tubes before the 1^st^ course of treatment

- 2 tubes at 4 weeks:

**in arm A&B** : before the 3^rd^ course of Nal-IRI plus 5-FU/LV

**OR**

**in arm C** after the 2^nd^ course of Nab paclitaxel + Gemcitabine

Blood samples will be used for extracting the DNA from the plasma (circulating tumor DNA) + buffy coat (genetic polymorphism)

***Sending tubes,*** via the box supplied at opening of the center:

Biological Resource Center EPIGENETEC

Unit UMR-S 1147

45 rue des Sts Pères, 75006 PARIS

Directed by Prof. Pierre LAURENT-PUIG

Use only the box containing the UPS form **addressed to the unit INSERM UMR-S 1147**

After sending this box, the box needed at inclusion of the next patient will be sent by the CRB EPIGENETEC.

**- Tumor block fixed in paraffin.**

Send the pre-filled letter to your anatomopathologist:

- The anatomopathologist will fax to the FFCD the sample sheet to the (0) 3 80 38 18 41
- FFCD will send a letter to send blocks

**Blocks (or slides) are sent to :**

Biological Resource Center EPIGENETEC

Unit UMR-S 1147

45 rue des Sts Pères, 75006 PARIS

Directed by Prof. Pierre LAURENT-PUIG

In case of questions or logistic problems, contact Claire MULOT at 01 42 86 38 61, [claire.mulot@parisdescartes.fr](mailto:claire.mulot@parisdescartes.fr) or contact FFCD at (0)3 80 39 34 86

# PATIENT MONITORING

## 9.1. During treatment

### 9.1.1. Before each administration of treatment:

Clinical examination

- Vital signs: BP, pulse, temperature
- Weight, body Area
- WHO
- Safety evaluation (toxicity according to NCI-CT v4.0) including systematic evaluation of the peripheral neuropathy according to the NCI-CT v4.0 scale (Appendix 6)

Laboratory assessment:

- FBC, platelets bilirubin (total and conjugated)
- Blood electrolytes, urea, creatinine and creatinine clearance (MDRD formula)
- INR, aPTT

### 9.1.2. Evaluation every 8 weeks until progression

Patients will be evaluated every 8 weeks (regardless of the number of cycles received) for:

Clinical examination

- Vital signs: BP, pulse, temperature
- Weight, body area
- WHO
- Safety evaluation (toxicity precedent cycle according to NCI-CT v4.0)
- Evaluation of the peripheral neuropathy according to the NCI-CT v4.0 scale and neurotoxicities using mTNS simplified scale (Appendix 6)
- QLQ-C30 version 3.0 every 2 months (Appendix 3)
- Pain assessment by VAS

Laboratory assessment

- FBC, platelets bilirubin (total and conjugated)
- Blood electrolytes, urea, creatinine and creatinine clearance (MDRD formula) (Appendix 4)
- INR, aPTT
- Liver panel comprising GGT, ALP, AST, ALT, total and conjugated bilirubin, and LDH
- INR, aPTT, blood protein, albumin and prealbumin
- CEA, CA 19.9

Morphological assesment:

- Thoracic-abdominal-pelvic CT (or thoracic CT and abdominal-pelvic MRI if IV contrast-enhanced CT is contraindicated) measuring tumor targets according to RECIST criteria (version 1.1, Appendix 5). (same modality as the one used for the initial assessment)

## 9.2. After treatment discontinuation

### 9.2.1. **Within 30 days** for evaluating the toxicity of the last treatment:

Biological tests:

- FBC, platelets, bilirubin (total and conjugated), PT, PAL, ASAT, ALAT, sodium, potassium, calcium, serum creatinine, MDRD creatinine clearance, albuminemia, LDH
- Evaluation of toxicities from the preceding cycle
- Pregnancy test

### 9.2.2. After radiological and/or clinical progression, patients will be monitored every 2 to 3 months up to death:

Clinical exam:

- Weight, WHO
- Evaluation of persistent toxicities
- Evaluation of the peripheral neuropathy 2 months after stopping the study treatment by mTNS simplified scale (Appendix 6)
- CAP-CT scan (or MRI)
- Quality of life questionnaire QLQ-C30 version 3.0 (Appendix 3)

### 9.2.3. **After premature discontinuation of treatment other than for progression***

### Patients will be monitored in the same way every 8 weeks until progression:

Clinical exam:

- Weight, WHO
- Evaluation of persistent toxicities
- Evaluation of peripheral neuropathy toxicities 2 months after stopping the study treatment by mTNS simplified scale (Appendix 6)
- CAP-CT scan (or MRI)
- Quality of life questionnaire QLQ-C30 version 3.0 (Appendix 3)
- Tumoral markers (CEA, CA 19-9)

* Toxicity, withdrawal of consent, lost to follow-up, patient refusal, medical decision, pregnancy or suspected pregnancy

# 10. SUBSEQUENT TREATMENTS

If chemotherapy is discontinued early, any subsequent treatment is at the investigator's discretion. However, for patients treated with Nab-placlitaxel plus Gemcitabine, Folfirinox or Folfiri shall be discussed depending on patient conditions.

Data on subsequent chemotherapy will be entered into the CRF so as to assess its impact on overall survival. The following information will be recorded for each subsequent line of treatment:

- Start date (D1) of the first cycle
- Date of D1 of the last cycle
- Drugs used
- Best response obtained

Data on other treatments, such as radiotherapy, radiofrequency and surgery, will also be entered into the CRF.

# 11. MANAGEMENT OF SERIOUS ADVERSE EVENTS

***Parameters for assessing safety***

Safety will be assessed by evaluating the clinical and biological health status of patients during visits and by recording events that occur between visits. Toxicities will be evaluated using the NCI-CTCAE toxicity scale (version 4.0) (Appendix 6) and using the simplified mTNS scale for neutotoxicities.

In case of emergency, the patient, the patient's family or the patient's physician must call the investigator to make it known that an event has occurred.

***Definitions***

Adverse event (AE)

An AE is an untoward medical occurrence in a person enrolled in a clinical trial, whether this occurrence is related or not to the trial itself or to the study product.

All AEs will be recorded in the CRF in the pages provided.

Serious adverse event (SAE)

An SAE is any event that meets at least one of the following criteria:

- results in death
- is life-threatening
- results in hospitalization or prolongs hospitalization
- causes permanent disability or serious temporary incapacity
- causes a congenital anomaly, fetal malformation or an abortion
- is medically significant

The terms disability and incapacity mean any temporary or permanent physical or mental disability that is clinically significant and that impacts the physical activity and/or quality of life of the patient.

A significant medical event is any clinical event or laboratory result considered to be serious by the investigator that does not meet the seriousness criteria defined above. It may put the patient at risk and require medical intervention to prevent an outcome such as one of the criteria for seriousness previously mentioned. Examples include overdose, second cancers, pregnancy and new facts that may be considered to be medically significant.

Pregnancy is a non-inclusion criterion in this trial and a reliable method of birth control must be used during the treatment and 3 months after. However, if a pregnancy occurs after a female patient’s enrollment, this patient must discontinue the trial. The sponsor should be noticed of this pregnancy with the SAE report form (no seriousness criteria must be filled). The patient will be followed until the end of the pregnancy and the outcome of the pregnancy must be reported to the sponsor. If a pregnancy occurs in partner of male patient enrolled on the trial, the sponsor should be noticed and will try, as possible, to follow the pregnancy.

Adverse effect

Any harmful, undesired reaction to a study drug regardless of the dose administered or to any investigational element. It is serious if it meets at least one of the seriousness criteria.

Unexpected SAE

An unexpected SAE is an event that is not mentioned in, or that differs in nature, intensity or outcome from, the product's reference document or smPC.

New fact

A new fact may be an unexpected frequency of an expected SAE or an SAE related to the trial procedure, insufficient efficacy in life-threatening diseases, or clinical data.

Severity (or intensity)

Severity must not be confused with seriousness, which serves as a guide defining reporting obligations.

The severity of an event will be assessed according to the extract of the CTCAE classification (version 4.0) (Appendix 6). The severity of adverse events not listed in this classification will be assessed using the following terms:

- Mild (grade 1): does not affect the patient's routine daily activities
- Moderate (grade 2): hinders the patient's routine daily activities
- Severe (grade 3): stops the patient's routine daily activities
- Very severe (grade 4): requires resuscitative action/endangers the patient's life
- Death (grade 5)

Causal relationship

Related: an event is said to be "related" when a causal relationship between the event and the product being studied may reasonably be suspected

Unrelated: an event is said to be "unrelated" when a causal relationship between the event and the product being studied cannot reasonably be suspected

Doubtful: the causal relationship is said to be "doubtful" if there are doubts about the causal relationship between the event and the product being studied. The relationship cannot be positively ruled out or confirmed.

Sponsor's responsibilities

As soon as the sponsor receives the SAE report made by the investigator, he has to assess the causal relationship between the SAE and the study product(s).

If the investigator and/or sponsor considered the SAE as related to one of the study products, it is therefore a serious adverse effect, and the sponsor must determine whether the effect is expected or unexpected.

If it is an unexpected serious adverse effect or a new fact, the sponsor drafts an initial report which is sent to the ANSM, IRB and EMA (via EudraVigilance) without delay (in the event of death or of a life-threatening situation) or within 15 days (in other cases).

If it is an expected serious adverse effect, it is compiled for the purpose of drafting the biannual and annual safety reports.

**Events that must not be considered serious**

Disease progression must not be considered an SAE.

Events that may be related to progression but may also have been caused by the treatment still need to be reported, for example thromboembolic events, hemorrhage, or perforation.

Because of the seriousness of the disease in this study, certain conditions defined as SAEs will be excluded from the SAE reporting procedure. These comprise:

- Hospitalization or surgery that are specifically connected with treating the disease. However, hospitalization or the prolonging of hospitalization due to a complication of such treatments must be reported as SAEs.

- Hospitalization to simplify the study treatments or procedures.

The reference documents in this trial will be:

For Nab-paclitaxel : Abraxane ® SmPC (Appendix 7)

For 5-FU : FLUOROURACILE EBEWE® SmPC (Appendix 7)

For gemcitabine : GEMZAR 1000 mg® SmPC (Appendix 7)

For leucovorin : ELVORINE® SmPC (Appendix 7)

For Nal-IRI : ONYVIDE® SmPC (Appendix 7) and section 5.5 of Investigator’s Brochure IRINOTECAN LIPOSOME INJECTION

The versions of the SmPCs that we will use to define expectedness or unexpectedness will be those in effect at the time of analysis.

***Procedure***

The investigator reports all SAEs to the sponsor, whether expected or unexpected, and whether related to the trial or not, that occur during the study or within 30 days of the last administration of treatment.

Any late SAEs (occurring after this 30-day period) that are reasonably related to the study drugs or to the trial must be reported regardless of when they occur.

The report is filed by faxing the "Serious Adverse Event Report" form (Appendix 8), dated, signed and documented as soon as possible, within 24 working hours of the event being observed to the **FFCD data center on +33 (0)3 80 38 18 41.**

The investigator must follow the patient until the event resolves or stabilizes or until the patient dies. It may sometimes require a follow-up of the patient after the trial discontinuation.

The investigator sends additional information to the sponsor using the SAE report form, ticking the "follow-up" box and increasing the number of the report to highlight that it is a follow-up report and not an initial report. These follow-up reports must be sent within 24 hours of receiving the information. The investigator also sends the last follow-up report when the SAE has resolved or stabilized.

The investigator handles requests for additional information to document the initial observation.

# 12. STATISTICAL ANALYSIS

## 12.1. Endpoints

### 12.1.1. Primary efficacy endpoint

The primary endpoint is the rate of patients alive without progression at 6 months after inclusion. The progression is defined as radiological and/or clinical progression assessed by the investigator according to RECIST v1.1 criteria. The delay will be defined from the date of randomization until progression or death (for whatever reason) or date of last news.

### 12.1.2. Secondary endpoints

The secondary endpoints are:

**Best Objective Response (BOR)**: BOR is defined as complete or partial response rate according to scans and RECIST v1.1 criteria over the entire treatment period.

**Progression free survival (PFS)**: PFS is defined as the time between the date of randomization and the date of the first radiological and/or clinical progression or the date of death (for whatever reason). Patients living without progression will be censured at date of last news. Progression is assessed by investigator and central review according to RECIST v1.1 criteria.

**Overall survival (OS)**: OS is defined as the time between the date of randomization and the date of death (whatever the cause). Alive patients will be censured at date of last news.

**Time to treatment failure** is defined as the time between the date of randomization and the date of discontinuation of all protocol treatments (regardless of cause) or date last news for patients alive under treatment.

**Safety:** Toxicities are evaluated according to NCI-CTC v4.0.

**Quality of life (EORTC QLQ-C30)**: Quality of life will be assessed according to the questionnaire of EORTC QLQ-C30. This scale comprises 30 items with 15 dimensions for calculating 15 scores (5 functional ability scores, 8 symptom scores, an overall score, and a financial problems score). These scores will be calculated and described at inclusion. Of an exploratory manner, time to deterioration of the overall health score will be calculated: it is defined as the time interval between the date of randomization and the date of reduction of over 5 points compared to the baseline (5 points being considered the minimum to define a clinically significant difference) or death.

**Evolution of tumoral markers**: The evolution of the markers will be analysed by a graphical representation at each time points of the percentage change from baseline.

## 12.2. Sample size Calculation, statistical hypotheses

The hypotheses used to calculate the sample size are:

H_0_: The rate of patients alive without progression at 6 months is not different between arms.

H_1_: The rate of patients alive without progression at 6 months in experimental arms is different from the rate in standard arm (same hypotheses for the 2 experimental arms). We expected a difference of 15% at 6 months in favour of experimental arms vs standard arm (from 40% to 55%); HR=0.65

With a two-sided α risk of 5% and a power of 80%, 170 events (progression or death) will be needed (estimated sample sizes for two-sample comparison of survivor functions Log-rank test, Schoenfeld method). Taking into account an assumption of 2 years of recruitment, a patient's follow-up of 3 years and with a percentage of patients lost to follow-up of 10%, it will be necessary to randomize 190 patients to compare an experimental arm vs. a reference arm (which means 95 patients in each arm, **285 patients overall = 95 patients*3arms**).

## 12.3. Statistical analysis plan

A more detailed Statistical Analysis Plan (SAP) will be written before the database lock.

### 12.3.1. Populations definitions

The **intention-to-treat (ITT)** population is defined as all patients randomized whatever their eligibility criteria.

The **modified intention-to-treat (mITT)** population is defined as all patients randomized whatever their eligibility criteria and who have received at least one dose of treatment in the study (analysis of the primary efficacy endpoint).

A **Per-Protocol (PP)** analysis of the primary endpoint will also be used. The population Per Protocol is defined as all randomized patients without major deviation and who received at least one dose of treatment.

The **Safety Population (SP)** is defined as all randomized patients whatever their eligibility criteria and who have received at least one dose of chemotherapy (regardless of the product).

### 12.3.2. Baseline analysis

Baseline characteristics will be described using descriptive statistics. The results will be presented by treatment arm and on the overall population.

The quantitative variables are described by the usual statistics as mean (with standard deviation), median (with interval Inter-quartiles) and min-max.

The qualitative variables will be described using patient numbers and percentages.

Comparisons by treatment arm will be performed for the quantitative variables, using a Student or Wilcoxon test (according to the distribution of the variables) and for qualitative variables, using a Chi² test or a Fisher exact test.

### 12.3.3. Efficacy analysis

**Main criterion:**

The overall effect of the treatment will be tested :

***H0 : % pts (Arm C) = % pts (Arm B) = % pts (Arm A)***

Secondary comparisons will only be made if the overall effect of treatment is significant.

Two main hypotheses related to the efficacy of different experimental arms for PFS, will be tested by demonstrating the superiority of each arms against standard arm.

***H0_2.1_: % pts (Arm C) = % pts (Arm B),***

***H0_2.2_: % pts (Arm C) = % pts (Arm A),***

where *%pts(G_i_)* corresponds to the percentage of patients alive and without progression at 6 months in the treatment group *G_i_*.

The alternative hypotheses are expressed as:

***H1_2.1_: % pts (Arm C) ≠ % pts (Arm B),***

***H1_2.2_: % pts (Arm C) ≠ % pts (Arm A),***

The statistical method proposed to demonstrate the superiority of experimental arms against standard will be the step-down procedure (22-23) and Sime’s method (24). (See SAP for more details).

The rate of patients alive without progression at 6 months will be estimated according to Kaplan-Meier estimation.

**Secondary endpoints:**

Censored data will be estimated and plotted using the Kaplan-Meier method. The median times and rates at different temporalities shall be described as well as their confidence intervals at 95%. Arms comparisons will be done using log-rank test.

The hazard ratio for the treatment effect will be calculated using a Cox model (Cox, 1984). Log-linearity assumptions and risk proportionality will be verified graphically thanks to residuals (Schoenfeld and Martingale residuals).

Median follow-up will be determined by the reverse Kaplan-Meier method.

Qualitative parameter (Best ORR) will be described by usual statistics and compared using Chi² test or Fischer exact test.

### 12.3.4. Safety analysis

The number of treatments, the dose received and the percentages of actual dose received on theoretical dose will be described, as well as the percentage of patients with at least one dose modification or at least an administrative report.

The toxicities will be described by treatment arm, the number of patients according to various grades and SOC. They will also be described by grouping grades 1-2 versus 3-4-5.

# 13. STUDY COMMITTEES

## 13.1. Independent committee

An independent committee will be established that comprises at least two gastroenterologists, a statistician or methodologist, and an expert in pharmacovigilance.

The independent committee will meet at least once a year, or more often if the sponsor deems it necessary in light of SAE analysis. It may also be convened at any time during the trial whenever the sponsor considers there to be a need to do so.

The committee will issue decisions on all safety data sent to the sponsor by the centers (AEs and/or SAEs). It will evaluate all patients included in the trial in the 2 months preceding the date of its meeting.

## 13.2. Steering committee

A steering committee will be set up. The chairperson of the steering committee will be the study coordinator. This committee will also comprise the co-coordinators, the FFCD study project manager, an FFCD statistician, and the chairperson of the biological research committee. Its functions will include, among other things, issuing decisions on the management of the research, such as amendments or, if needed, early trial closure. The steering committee will meet as often as required throughout the study. It will make the necessary decisions concerning substantial protocol amendments, trial closure or trial extension.

## 13.3. Medical review

A medical review committee will be set up to improve the quality of clinical data collected. If there is a discrepancy between the data provided by the investigator and those provided by the medical review committee, data management will seek clarification from the investigator.

## 13.4. Biological research committee

A biological research committee will be established whose role is to answer questions about sample-taking and storage as well as the organization of sample analysis. The committee will meet regularly and report its proposals to the steering committee. This committee will comprise, among others, the study coordinator and a biologist. The committee chairman will be Pr Julien Taieb.

# 14. Background information and rationale for the trial

Pancreatic cancer (PC) is the seventh cause from cancer and the fifth cause from cancer-related death in Europe(1,2). Nearly as many deaths occur from PC than the number of new cases diagnosed each year, reflecting the poor prognosis typically associated with this disease. PC is insidious in onset and is often diagnosed late at the stage of metastatic spread. In spite of advances made in the management of other more common gastrointestinal cancers, the treatment of PC did take only a small advantage of recent progresses in gastrointestinal oncology and targeted therapies did not significantly modify its prognosis to date (3).

Thus, for more than 10 years, gemcitabine has been the standard of care to treat metastatic PC (4). Recently, two positive phase III trials in metastatic PC were reported.

First, the gemcitabine with nab-paclitaxel combination therapy was compared to gemcitabine alone in 861 randomized patients with metastatic PC. Results showed a significant improvement in response rate (RR), progression free survival (PFS) and overall survival (OS) (5). Second, the results of the PRODIGE 4/ACCORD 11 trial testing the FOLFIRINOX regimen in metastatic PC patients, finally gave a hope showing a major improvement in PFS and OS (6) as compared to gemcitabine. However, though manageable, the safety profiles of FOLFIRINOX and of gemcitabine plus nab-paclitaxel were less favourable than that of gemcitabine. These regimens were associated with a higher incidence of grade 3-4 neutropenia, febrile neutropenia, thrombocytopenia, diarrhoea, and grade 2-3 sensory neuropathy.

Irinotecan has a stronger growth-inhibiting effect than cisplatin, mitomycin C and fluorouracil on cultured pancreatic adenocarcinoma cells (7). In most trials testing this molecule in PC patients, however, the response rates were low (<10%) and survivals were poor (8–11). Intensive regimens with irinotecan and 5-FU have been developed in colorectal cancer patients (12,13) to increase the anti-tumor effects of this combination therapy. From those, the FOLFIRI.3, in which the irinotecan is administered before and after a 5-FU 46h continuous infusion gave promising results (14). We have tested this regimen in a phase II trial in advanced PC patients, with an objective RR of 37.5%, a median OS time of 12 months and acceptable tolerability (15). Considering these encouraging results we have proposed a new approach to improve outcomes for patients with metastatic PC, using FOLFIRI.3 and gemcitabine alone, sequentially, to increase patient survival with a preserved quality of life (QOL). Indeed, some authors have reported that the administration of different patterns of sequential polychemotherapy was independently associated with OS in patients with PC and other gastrointestinal tumors such as metastatic colorectal cancer (16,17). Such strategies using drugs without cross-resistance sequentially may increase anti-tumor effects and limit cumulative and non-cumulative toxicities.

We thus performed a randomized multicentre phase II trial to assess this sequential treatment strategy using FOLFIRI.3 and gemcitabine alternately in one arm and gemcitabine alone in the other arm, in patients with metastatic non pre-treated pancreatic adenocarcinoma (18). In this study the FIRGEM strategy seems to be an effective first line treatment option in good condition patients with metastatic PC. The primary endpoint was reached with a rate of PFS at 6 months of 44.9% in the FIRGEM arm while gemcitabine alone failed (25.7%). This good PFS rate at 6 months was maintained at 12 and 18 months (26.2% and 18% respectively) though median PFS was 5.0 months. Moreover, an impressive objective response rate was observed in the FIRGEM arm (40%) as compared to the gemcitabine arm (11.4%). These results confirm those of the initial phase II trial evaluating the FOLFIRI.3 regimen (37.5% objective response rate)(15) in PC patients and compare favourably with the 31.6% and 23% reported in the two trials evaluating FOLFIRINOX and gemcitabine + nab-paclitaxel, respectively.

Median overall survival was 11 months with FIRGEM versus 8.2 months with gemcitabine (HR:0.710 95% CI: 0.457-1.103). Here again the experimental arm gave good results, with median OS in the range of those reported with FOLFIRINOX (11.1 months). The safety profile of the FIRGEM strategy showed that hematological and GI toxicities were more important than with gemcitabine alone. Interestingly no limiting sensory neuropathy was observed with our treatment schedule and a significant increase in the time to definitive deterioration of the QoL was observed in the FIRGEM group as compared with the gemcitabine group. This effect was observed for all domains.

Considering that nab-paclitaxel improves significantly patients outcome in metastatic PC when combined to gemcitabine, the addition of this drug to the FIRGEM strategy may be of particular interest. And the PRODIGE 37 trial tested this combination in a randomized phase II trial recently closed for inclusions. This trial allowed to fight the cancer with 4 different drugs, without any cross resistance described between them, given sequentially in the first 4 months of treatment. Moreover, the “resting period” without nab-paclitaxel may delay significantly the occurrence of the cumulative neuropathy induced by this molecule and optimised its used with such a “stop and go” like strategy. Results are awaited for early 2018.

More recently a liposomal irinotecan has been developed and tested in pancreatic cancer patients, it comprises irinotecan free base encapsulated in liposome nanoparticles, which keep irinotecan into the circulation sheltered from conversion to its active metabolite (SN-38) longer and use local macrophage-mediated activation, which would increase and prolong intratumoral levels of both irinotecan and SN-38 (19). In a phase II study of 40 patients with MPA previously treated with gemcitabine-based therapy, monotherapy with nal-IRI resulted in a median overall survival of 5.2 months, and a manageable toxicity profile (20). The NAPOLI-1 phase III trial was then conducted, comparing three arms of chemotherapy in patients previously treated with gemcitabine-based therapy: liposomal irinotecan (MM-398 or nal-IRI) alone or combined with 5FU and folinic acid, and 5FU and folinic acid alone (21). Combination of nal-IRI and 5FU/LV was more effective than 5FU alone or Nal-IRI alone (median OS of 6.1 vs. 4.2 and 4.9 months respectively (HR: 0.67; p=0.012). Increased hematologic and GI toxicities were also seen in the Nal-IRI arms but were manageable. Nal-IRI plus 5FU and folinic acid extends survival in patients with metastatic pancreatic ductal adenocarcinoma who previously received gemcitabine-based therapy. In a setting where there is a paucity of second line treatment option, this combination is an important emerging treatment option for metastatic adenocarcinoma of the pancreas.

Considering all these data, we propose to run a randomized phase II trial testing the standard continuous Gemcitabine + Nab-paclitaxel schedule vs Nal-IRI+5FU/LV vs Nal-IRI+5FU/LV for two months followed by Gemcitabine + Nab-paclitaxel for two months before starting again Nal-IRI. This will allow i) to generate efficacy and tolerability data on the Nal-IRI/5FU combination in the first line setting, ii) to test a new sequential strategy with Nal-IRI , in regards of the interesting results obtained in second and third line pancreatic cancer treatment, iii) to control our results in the experimental arms with one of the two first line worldwide standard regimen: Gemcitabine + Nab-paclitaxel.

# 15. Bibliographical references

1. Ferlay J, Parkin DM, Steliarova-Foucher E. Estimates of cancer incidence and mortality in Europe in 2008. Eur. J. Cancer. mars 2010;46(4):765‑781.

2. Jemal A, Siegel R, Xu J, Ward E. Cancer statistics, 2010. CA Cancer J Clin. oct 2010;60(5):277‑300.

3. Trouilloud I, Dubreuil O, Boussaha T, Lepère C, Landi B, Zaanan A, et al. Medical treatment of pancreatic cancer: new hopes after 10 years of gemcitabine. Clin Res Hepatol Gastroenterol. mai 2011;35(5):364‑374.

4. Burris HA, Moore MJ, Andersen J, Green MR, Rothenberg ML, Modiano MR, et al. Improvements in survival and clinical benefit with gemcitabine as first-line therapy for patients with advanced pancreas cancer: a randomized trial. J. Clin. Oncol. juin 1997;15(6):2403‑2413.

5. Von Hoff et al. Randomized phase III study of weekly nab-paclitaxel plus gemcitabine versus gemcitabine alone in patients with metastatic adenocarcinoma of the pancreas (MPACT). NEJM 2014

6. Conroy T, Desseigne F, Ychou M, Bouché O, Guimbaud R, Bécouarn Y, et al. FOLFIRINOX versus gemcitabine for metastatic pancreatic cancer. N. Engl. J. Med. 12 mai 2011;364(19):1817‑1825.

7. Matsuoka H, Yano K, Seo Y, Saito T, Tomoda H, Takiguchi S, et al. Cytotoxicity of CPT-11 for gastrointestinal cancer cells cultured on fixed-contact-sensitive plates. Anticancer Drugs. juin 1995;6(3):413‑418.

8. Wagener DJ, Verdonk HE, Dirix LY, Catimel G, Siegenthaler P, Buitenhuis M, et al. Phase II trial of CPT-11 in patients with advanced pancreatic cancer, an EORTC early clinical trials group study. Ann. Oncol. févr 1995;6(2):129‑132.

9. Klapdor R, Fenner C. Irinotecan(Campto R): efficacy as third/forth line therapy in advanced pancreatic cancer. Anticancer Res. déc 2000;20(6D):5209‑5212.

10. Ulrich-Pur H, Raderer M, Verena Kornek G, Schüll B, Schmid K, Haider K, et al. Irinotecan plus raltitrexed vs raltitrexed alone in patients with gemcitabine-pretreated advanced pancreatic adenocarcinoma. Br. J. Cancer. 22 avr 2003;88(8):1180‑1184.

11. Zaniboni A, Aitini E, Barni S, Ferrari D, Cascinu S, Catalano V, et al. FOLFIRI as second-line chemotherapy for advanced pancreatic cancer: a GISCAD multicenter phase II study. Cancer Chemother. Pharmacol. juin 2012;69(6):1641‑1645.

12. Hebbar M, Ychou M, Ducreux M. Current place of high-dose irinotecan chemotherapy in patients with metastatic colorectal cancer. J. Cancer Res. Clin. Oncol. juin 2009;135(6):749‑752.

13. Ducreux M, Raoul J-L, Marti P, Merrouche Y, Tigaud J-M, Rebischung C, et al. High-dose irinotecan plus LV5FU2 or simplified LV5FU (HD-FOLFIRI) for patients with untreated metastatic colorectal cancer: a new way to allow resection of liver metastases? Oncology. 2008;74(1-2):17‑24.

14. Mabro M, Artru P, André T, Flesch M, Maindrault-Goebel F, Landi B, et al. A phase II study of FOLFIRI-3 (double infusion of irinotecan combined with LV5FU) after FOLFOX in advanced colorectal cancer patients. Br. J. Cancer. 8 mai 2006;94(9):1287‑1292.

15. Taïeb J, Lecomte T, Aparicio T, Asnacios A, Mansourbakht T, Artru P, et al. FOLFIRI.3, a new regimen combining 5-fluorouracil, folinic acid and irinotecan, for advanced pancreatic cancer: results of an Association des Gastro-Enterologues Oncologues (Gastroenterologist Oncologist Association) multicenter phase II study. Ann. Oncol. mars 2007;18(3):498‑503.

16. Kelly H, Goldberg RM. Systemic therapy for metastatic colorectal cancer: current options, current evidence. J. Clin. Oncol. 10 juill 2005;23(20):4553‑4560.

17. Klapdor R, Bahlo M, Babinsky A. Further evidence for prolongation of survival of pancreatic cancer patients by efficacy orientated sequential polychemotherapy (EOSPC) based on serial tumor marker determinations (CA 19-9/CEA). Anticancer Res. juin 2005;25(3A):1687‑1691.

18. Trouilloud I, Dupont-Gossard et al. [Fixed-dose rate gemcitabine alone or alternating with FOLFIRI.3 (irinotecan, leucovorin and fluorouracil) in the first-line treatment of patients with metastatic pancreatic adenocarcinoma: an AGEO randomised phase II study (FIRGEM).](https://www.ncbi.nlm.nih.gov/pubmed/25454414) Eur J Cancer. 2014 Dec;50(18):3116-24

19. Kalra AV, Kim J, Klinz SG, Paz N et al. [Preclinical activity of nanoliposomal irinotecan is governed by tumor deposition and intratumor prodrug conversion.](https://www.ncbi.nlm.nih.gov/pubmed/25273092) Cancer Res. 2014 Dec 1;74(23):7003-13.

20. Ko AH, Tempero MA, Shan YS et al. A multinational phase 2 study of nanoliposomal irinotecan sucrosofate (PEP02, MM-398) for patients with gemcitabine-refractory metastatic pancreatic cancer. Br J Cancer. 2013 Aug 20;109(4):920-5.

21. [Wang-Gillam A](https://www.ncbi.nlm.nih.gov/pubmed/?term=Wang-Gillam%20A%5BAuthor%5D&cauthor=true&cauthor_uid=26615328), [Li CP](https://www.ncbi.nlm.nih.gov/pubmed/?term=Li%20CP%5BAuthor%5D&cauthor=true&cauthor_uid=26615328), [Bodoky G](https://www.ncbi.nlm.nih.gov/pubmed/?term=Bodoky%20G%5BAuthor%5D&cauthor=true&cauthor_uid=26615328) et al.. Nanoliposomal irinotecan with fluorouracil and folinic acid in metastatic pancreatic cancer after previous gemcitabine-based therapy (NAPOLI-1): a global, randomised, open-label, phase 3 trial. Lancet. 2016 Feb 6;387(10018):545-57.

22. Bauer P. (1991). Multiple testing in clinical trials. *Statistics in Medicine,* 10 : 871-890

23. Bauer P. (1993). Multiple primary treatment comparisons based on closed tests. *Drug Information Journal,* 27 : 643-649

24. Simes, R. J. (1986). An improved Bonferroni procedure for multiple tests of significance. *Biometrika*, 63, 655-660.

# 16. ADMINISTRATIVE CONSIDERATIONS

**Trial sponsor**

The trial sponsor is the FFCD. The trial is registered under the EudraCT number 2017-004309-41.

**Reminder concerning applicable regulations**

This trial will be conducted in accordance with current French law, with the ethical principles of the Helsinki Declaration of 1964 and its subsequent revisions, with Good Clinical Practice of the International Conference on Harmonization (ICH–E6, 17/07/96), with European directive 2001/20/EC on the conduct of clinical trials, with the Huriet Act as amended (20/12/88) on the protection of persons participating in biomedical research, and with the provisions laid down by the CNIL, the French data protection agency (Act no. 94-548 of 01/07/94 completing Act no. 78-17 of 06/01/78) and the European Regulation 2016/679 of 27 April 2016.

**Public liability insurance**

An insurance policy was taken out by the sponsor on 04/09/2018 under the number 137.681, in accordance with article L 1121-10 of the French Public Health Code (Appendix 10).

**Application for approval from the IRB and ANSM**

This protocol received approval from IRB SUD EST II on 27/06/2018 (Appendix 11).

This protocol received authorization from the ANSM on 04/05/2018 (Appendix 12).

**Collecting patient consent**

The investigator undertakes to provide the patient with information and to collect written clinical and biological consent from the patient (using the information sheets and consent forms in appendices 1 and 2) before registering the patient in the study. A copy of these consent forms must be retained by the investigator for 15 years so that they may be shown to the regulatory authorities in the event of inspection. The originals must be given back to the patient.

In accordance with the recommendations of the French cancer action plan (measure 5.1), this document was submitted to the Patient Advisory Board for Clinical Research of the French League Against Cancer.

**Notification of hospital senior management and clinical trial agreement**

Before the trial is launched, the sponsor will inform the senior management of the hospitals of the utility for the investigator of taking part in this trial.

A clinical trial agreement (which includes no additional costs) will be drawn up between the administrator of the investigator center and the sponsor.

**Data archiving**

Files will remain confidential and may be consulted only under the authority of the physicians treating the patients. The sponsor and health authorities will have direct access to these documents in the event of an inspection.

The investigator will retain the study documents for 15 years after the end of the trial.

**Computerized archiving**

In accordance with the provisions of French Data Protection Act no. 78-17 of January 6, 1978, as amended by the Act of August 9, 2004, and the European Regulation 2016/679 of 27 April 2016, trial data will be recorded in a computerized database of the Randomization, Management and Analysis Center of the FFCD, with the exception of items relating to patient identity.

**Data processing**

The Randomization, Management and Analysis Center of the FFCD will be responsible for managing and analyzing data. The Randomization, Management and Analysis Center of the FFCD ensures that randomization procedure and data will be managed according to the MR-001.

**Monitoring, quality assurance and inspections by the authorities**

The investigator hereby accepts that the files of patients enrolled may be consulted by any person appointed by the sponsor and/or by the health authorities to carry out an audit. Visits to inspect the files on site, which are scheduled with the investigator's agreement, may be made during or after the trial inclusion period.

This protocol will be monitored by traveling CRAs of the FFCD.

# 17. [Rules for publication](file:///C:\Users\Marie\AppData\Local\Microsoft\AppData\Local\Microsoft\Windows\AppData\Local\Microsoft\Windows\AppData\Local\Microsoft\Windows\Temporary%20Internet%20Files\Content.Outlook\AppData\Local\AppData\Local\Microsoft\AppData\Local\Microsoft\Windows\AppData\Local\Microsoft\claire.nee\AppData\Local\Microsoft\Windows\Temporary%20Internet%20Files\claire.nee\AppData\Local\Microsoft\Windows\Temporary%20Internet%20Files\Local%20Settings\AppData\Local\Microsoft\AppData\Local\Microsoft\Windows\AppData\Local\Microsoft\Windows\AppData\Local\Microsoft\Windows\Temporary%20Internet%20Files\Local%20Settings\Temp\REGLES%20PUBLICATION%20PRODIGE%20v%20finale%20.doc)

They will comply with those laid down by the PRODIGE research group (Appendix 9).

# 18. APPENDICES

APPENDIX 1: CLINICAL and BIOLOGICAL INFORMED CONSENT

APPENDIX 2: BIOLOGICAL STUDIES

**1/ RATIONNAL**

**Circulating Tumor DNA (ctDNA)**

The ability to detect and characterize circulating cell-free tumor DNA (ctDNA) provides a non-invasive method to monitor the genetic alterations present in tumor cells. This “liquid biopsy” is a major advance to characterize the genomic profile of patients with cancer, monitor treatment responses, quantify minimal residual disease, and assess the emergence of therapy resistance [Schwarzenbach et al. 2011, Misale et al. Nature 2012, Diaz LA et al. Nature 2012]. The detection of ctDNA and its validation as a biomarker of cancer requires highly sensitive methods since it is largely diluted in circulating normal DNA. ddPCR methods are well-suited to overcome the sensitivity problem: The research group of P.Laurent-Puig demonstrated a sensitivity of 1 mutated molecule among 200 000 non-mutated molecules. By applying this method, they were able to detect ctDNA in the plasma of patients with advanced colorectal cancer by characterizing the 7 most frequent KRAS mutations in 2 ddPCR assays (**Taly et al. Clin Chem 2013**). This method of ctDNA detection is based on the *a priori* knowledge of tumor DNA alterations. In order to be independent from this, we developed two other methods. One is based on NGS and enables to detect alteration up to 0.1% in a routinely based test that we developed (BPER-method -**Pecuchet et al. Clin Chem 2016**). The other one monitors epigenetic modifications. They showed for instance that the hypermethylation of the *WIF1* and *NPY* genes can be a universal marker of ctDNA in colorectal cancer (**Garrigou et al. Clin Chem 2016**). They set-up 3 prospective clinical studies to validate the use of ctDNA, and showed that the initial ctDNA concentration and its decrease during treatment is a prognostic marker in pancreatic cancer (**Pietrasz et al. Clin Cancer Res 2017),** lung cancer (**Pecuchet et al. Plos Med 2016**) and in colon cancer (Garlan et al. Clin Cancer Res in press).

In this study, we propose to evaluate the prognostic role of ctDNA before the initiation of treatment (T0). The presence and the quantification of ctDNA will be tested as a prognostic marker of pancreatic cancer overall survival. A second blood sample will be withdrawed after 4 weeks of therapy to detect a potential predictive value for treatment efficacy or inefficacy.

**2/ PRACTICAL MODALITIES**

**The ancillary study will have two components:**

**Study on tumoral blocks :** The blocks included in paraffin will be centralized to the CRB EPIGENETEC of the FFCD. After verification of histology using microscopy after staining with Hematoxylin and Eosin (H & E), EPIGENETEC will proceed to the constitution of a TMA (Tissue microarray) by sampling cores of tumor tissue. Each tumor will be represented by 2 samples with an average diameter of 600 μm. The characterization of the expression of the biomarkers by pancreatic exocrine adenocarcinomas will be carried out by an immunohistochemical technique using the following antibodies: Monoclonal Anti-Hent1 Ac of rabbit (Ventana-Roche®); Ac polyclonal anti-DCK rabbit (Abcam®); Ac polyclonal rabbit anti-CDA (Abcam®); Monoclonal anti-mouse S100A2 (R & D systems®); Rabbit anti-Smad4 monoclonal antibody (Abgent / Clinisciences®); Polyclonal anti-DPD rabbit (LSBio / Clinisciences®); Rabbit anti-TS monoclonal antibody (Abcam®). Other immunodetections may be proposed, by the end of the inclusions and the beginning of the biological study.

**For biological logistic, please refer to the chapter 8 of the protocol.**

**Bibliographical references for biological studies**

C. Serdjebi, JF. Seitz, J. Ciccolini, M. Duluc, E. Norguet, F. Fina, B. Lacarelle, L. Ouafik, L. Dahan. Rapid deaminator status is associated with poor clinical outcome in pancreatic cancer patients treated with a gemcitabine based regimen. Pharmacogenomics 2013 ; 14(9) :1047-51 (AO;3,8)

[Ciccolini J](http://www.ncbi.nlm.nih.gov/pubmed?term=Ciccolini%20J%5BAuthor%5D&cauthor=true&cauthor_uid=19933910), [Dahan L](http://www.ncbi.nlm.nih.gov/pubmed?term=Dahan%20L%5BAuthor%5D&cauthor=true&cauthor_uid=19933910), [André N](http://www.ncbi.nlm.nih.gov/pubmed?term=Andr%C3%A9%20N%5BAuthor%5D&cauthor=true&cauthor_uid=19933910), [Evrard A](http://www.ncbi.nlm.nih.gov/pubmed?term=Evrard%20A%5BAuthor%5D&cauthor=true&cauthor_uid=19933910), [Duluc M](http://www.ncbi.nlm.nih.gov/pubmed?term=Duluc%20M%5BAuthor%5D&cauthor=true&cauthor_uid=19933910), [Blesius A](http://www.ncbi.nlm.nih.gov/pubmed?term=Blesius%20A%5BAuthor%5D&cauthor=true&cauthor_uid=19933910), [Yang C](http://www.ncbi.nlm.nih.gov/pubmed?term=Yang%20C%5BAuthor%5D&cauthor=true&cauthor_uid=19933910), [Giacometti S](http://www.ncbi.nlm.nih.gov/pubmed?term=Giacometti%20S%5BAuthor%5D&cauthor=true&cauthor_uid=19933910), [Brunet C](http://www.ncbi.nlm.nih.gov/pubmed?term=Brunet%20C%5BAuthor%5D&cauthor=true&cauthor_uid=19933910), [Raynal C](http://www.ncbi.nlm.nih.gov/pubmed?term=Raynal%20C%5BAuthor%5D&cauthor=true&cauthor_uid=19933910), [Ortiz A](http://www.ncbi.nlm.nih.gov/pubmed?term=Ortiz%20A%5BAuthor%5D&cauthor=true&cauthor_uid=19933910), [Frances N](http://www.ncbi.nlm.nih.gov/pubmed?term=Frances%20N%5BAuthor%5D&cauthor=true&cauthor_uid=19933910), [Iliadis A](http://www.ncbi.nlm.nih.gov/pubmed?term=Iliadis%20A%5BAuthor%5D&cauthor=true&cauthor_uid=19933910), [Duffaud F](http://www.ncbi.nlm.nih.gov/pubmed?term=Duffaud%20F%5BAuthor%5D&cauthor=true&cauthor_uid=19933910), [Seitz JF](http://www.ncbi.nlm.nih.gov/pubmed?term=Seitz%20JF%5BAuthor%5D&cauthor=true&cauthor_uid=19933910), [Mercier C](http://www.ncbi.nlm.nih.gov/pubmed?term=Mercier%20C%5BAuthor%5D&cauthor=true&cauthor_uid=19933910). Cytidine deaminase residual activity in serum is a predictive marker of early severe toxicities in adults after gemcitabine-based chemotherapies. [J Clin Oncol.](http://www.ncbi.nlm.nih.gov/pubmed/19933910) 2010 Jan 1;28(1):160-5.

[Kondo N](http://www.ncbi.nlm.nih.gov/pubmed?term=Kondo%20N%5BAuthor%5D&cauthor=true&cauthor_uid=21538357), [Murakami Y](http://www.ncbi.nlm.nih.gov/pubmed?term=Murakami%20Y%5BAuthor%5D&cauthor=true&cauthor_uid=21538357), [Uemura K](http://www.ncbi.nlm.nih.gov/pubmed?term=Uemura%20K%5BAuthor%5D&cauthor=true&cauthor_uid=21538357), [Sudo T](http://www.ncbi.nlm.nih.gov/pubmed?term=Sudo%20T%5BAuthor%5D&cauthor=true&cauthor_uid=21538357), [Hashimoto Y](http://www.ncbi.nlm.nih.gov/pubmed?term=Hashimoto%20Y%5BAuthor%5D&cauthor=true&cauthor_uid=21538357), [Nakashima A](http://www.ncbi.nlm.nih.gov/pubmed?term=Nakashima%20A%5BAuthor%5D&cauthor=true&cauthor_uid=21538357), [Ohge H](http://www.ncbi.nlm.nih.gov/pubmed?term=Ohge%20H%5BAuthor%5D&cauthor=true&cauthor_uid=21538357), [Sueda T](http://www.ncbi.nlm.nih.gov/pubmed?term=Sueda%20T%5BAuthor%5D&cauthor=true&cauthor_uid=21538357). Prognostic impact of dihydropyrimidine dehydrogenase expression on pancreatic adenocarcinoma patients treated with S-1-based adjuvant chemotherapy after surgical resection. [J Surg Oncol.](http://www.ncbi.nlm.nih.gov/pubmed/21538357) 2011 Aug 1;104(2):146-54. doi: 10.1002/jso.21955. Epub 2011 Apr 27.

Kawakami K, Watanabe G. Identification and functional analysisof SNP in the tandem repeat sequence of TS gene. Cancer research 2003; 63:6004-7

Etienne MC, Lic K, Formento JL, et al. TS and MRHFR gene polymorphisms: relationships with 5FU sensitivity. Br J cancer 2004;90:526-34

APPENDIX 3: QUALITY OF LIFE – QLQ-C30

**EORTC QLQ-C30 (version 3.0) – PRODIGE 61 - FUNGEMAX**

We are interested in some things about you and your health. Please answer all of the questions yourself by **circling the number** that best applies to you. There are no "right" or "wrong" answers. The information that you provide will remain strictly confidential.

*Please fill in:*  **The first two letters of your surname:** αα

**The first letter of your first name:**  α **Visit number :** αα

**Your date of birth:** 

**Today's date:** 

|  | **Not at all** | **A little** | **Quite a bit** | **Very much** |
| --- | --- | --- | --- | --- |
| 1. Do you have any trouble doing strenuous activities, like carrying a heavy shopping bag or suitcase? | 1 | 2 | 3 | 4 |
| 2. Do you have any trouble taking a long walk? | 1 | 2 | 3 | 4 |
| 3. Do you have any trouble taking a short walk outside the house? | 1 | 2 | 3 | 4 |
| 4. Do you need to stay in bed or in a chair during the day? | 1 | 2 | 3 | 4 |
| 5 Do you need help with eating, dressing, washing yourself or using the toilet? | 1 | 2 | 3 | 4 |

**During the past week:**

|  | **Not at all** | **A little** | **Quite a bit** | **Very much** |
| --- | --- | --- | --- | --- |
| 6. Were you limited in doing either your work or other daily activities? | 1 | 2 | 3 | 4 |
| 7. Were you limited in pursuing your hobbies or other leisure time activities? | 1 | 2 | 3 | 4 |
| 8. Were you short of breath? | 1 | 2 | 3 | 4 |
| 9. Have you had pain? | 1 | 2 | 3 | 4 |
| 10. Did you need to rest? | 1 | 2 | 3 | 4 |
| 11. Have you had trouble sleeping? | 1 | 2 | 3 | 4 |
| 12. Have you felt weak? | 1 | 2 | 3 | 4 |
| 13. Have you lacked appetite? | 1 | 2 | 3 | 4 |
| 14. Have you felt nauseated? | 1 | 2 | 3 | 4 |
| 15. Have you vomited? | 1 | 2 | 3 | 4 |
| 16. Have you been constipated? | 1 | 2 | 3 | 4 |
| 17. Have you had diarrhea? | 1 | 2 | 3 | 4 |
| 18. Were you tired? | 1 | 2 | 3 | 4 |
| 19. Did pain interfere with your daily activities? | 1 | 2 | 3 | 4 |
| 20. Have you had difficulty in concentrating on things, like reading a newspaper or watching television? | 1 | 2 | 3 | 4 |
| 21. Did you feel tense? | 1 | 2 | 3 | 4 |
| 22. Did you worry? | 1 | 2 | 3 | 4 |
| 23. Did you feel irritable? | 1 | 2 | 3 | 4 |
| 24. Did you feel depressed? | 1 | 2 | 3 | 4 |
| 25. Have you had difficulty remembering things? | 1 | 2 | 3 | 4 |
| 26. Has your physical condition or medical treatment interfered with the family life? | 1 | 2 | 3 | 4 |
| 27. Has your physical condition or medical treatment interfered with your social activities? | 1 | 2 | 3 | 4 |
| 28. Has your physical condition or medical treatment caused you financial difficulties? | 1 | 2 | 3 | 4 |

**For the following questions, please circle the number between 1 and 7 that best applies to you**

29. How would you rate your overall health during the last week?

1 2 3 4 5 6 7

Very poor Excellent

30. How would you rate your overall quality of life during the last week?

1 2 3 4 5 6 7

Very poor Excellent

© Copyright 1999 EORTC Quality of Life Study Group, version 3.0. All rights reserved. English version

Les données recueillies seront analysées par informatique. Conformément à la réglementation applicable au traitement des données à caractère personnel, le règlement européen 2016/679 du 27 avril 2016 et la loi « Informatique et Liberté » du 6 janvier 1978 modifiée par la loi 2004-801 du 6 Août 2004, vous pourrez exercer notamment un droit d’accès et de modification par l’intermédiaire de votre médecin investigateur

Appendix 4: WHO performance status – CALCULATION OF CLEARANCE

**GENERAL CONDITION – WHO SCALE**

0 = able to carry on all pre-disease activities without restriction.

1 = restricted in physically strenuous activity, but ambulatory and able to carry out light work.

2 = ambulatory and capable of self-care but unable to carry out any work activities. In bed less than 50 % of the time.

3 = capable of just a few personal care activities. Bed-ridden or in a wheelchair more than 50% of the time.

4 = incapable of taking care of him/herself, permanently bed-ridden or in a wheelchair.

**CLEARANCE:**

**MDRD (*Modification of the Diet in Renal Disease*) formula (Levey, 2000):**
186.3 × (creatinine (in mmol/L)/88.4) × 1154 age-0203 (x 0.742 if female x 1.21 if black skin)

**NYHA CLASSIFICATION:**

| Class I | Cardiac disease, but no symptoms and no limitation in ordinary physical activity, e.g. no shortness of breath when walking, climbing stairs etc. |
| --- | --- |
| Class II | Mild symptoms (mild shortness of breath and/or angina) and slight limitation during ordinary activity. |
| Class III | Marked limitation in activity due to symptoms, even during less-than-ordinary activity, e.g. walking short distances (20–100 m). Comfortable only at rest. |
| Class IV | Severe limitations. Experiences symptoms even while *at rest*. Mostly bedbound patients. |

Appendix 5: RECIST criteria, version 1.1

"New response evaluation criteria in solid tumours: Revised RECIST guideline (version 1.1)" E.A. Eisenhauer, P. Therasse, J. Bogaerts, L.H. Schwartz, D. Sargent, R. Ford, J. Dancey, S. Arbuck, S. Gwyther, M. Mooney, L. Rubinstein, L. Shankar, L. Dodd, R. Kaplan, D. Lacombe, J. Verweij;

Eur J Cancer, 4 5 ( 2 0 0 9 ) 2 2 8 –2 4 7.

Lesions on inclusion:

Lesions and lymph nodes are classed individually as being measurable or non-measurable.

Measurable disease

A lesion is measurable if it can be accurately measured in at least one dimension (longest diameter in the plane of measurement is to be reported).

To be measurable, lesions must have a minimum size of

≥ 10 mm on CT (CT scan slice thickness no greater than 5 mm)

≥ 10 mm on clinical examination (measured using a caliper); lesions that cannot be accurately measured with calipers should be classed as non-measurable

20 mm on chest X-ray

For a malignant lymph node to be considered pathological and measurable, its short axis must measure ≥ 15 mm (the short axis is the axis perpendicular to the largest dimension of the lymph node). Only the length of this short axis is reported on inclusion and during follow-up.

Non-measurable disease

All other lesions, including small lesions (longest diameter < 10 mm on CT or lymph nodes with ≥ 10 mm and < 15 mm short axis) as well as truly non-measurable lesions: leptomeningeal disease, ascites, pleural or pericardial effusion, inflammatory breast disease, lymphangitic involvement of skin or lung, abdominal masses identified by physical examination but unconfirmed by imaging techniques, and cystic lesions.

NB: bone lesions, simple cystic lesions and lesions that have received prior local treatment require special consideration (see comments below).

Target lesions

Target lesions are selected from the measurable lesions presented by the patient on entry into the study. **A maximum of five target lesions are selected, with no more than two target lesions per organ.** Target lesions will be selected so as to be representative of all involved organs. The largest lesions (in the longest dimension) that may be repeatedly and reproducibly measured throughout the trial using the initial examination method are to be chosen. Lymph nodes may be considered as target lesions if their short axis as measured on CT is ≥ 15 mm.

The sum of the diameters of these target lesions (the longest diameter for lesions, and short axis for lymph nodes) is what is followed throughout the trial for assessing response or progression.

Non-target lesions

All other lesions are identified as non-target lesions and are also recorded on inclusion. They are not measured but they are followed throughout the trial.

###### Criteria for response to treatment:

**Target lesions:**

**Complete response (CR)** Disappearance of all lesions, and all lymph nodes (whether target or non-target) must have reduction in *short axis* to < 10 mm.

*Note: lymph nodes selected as target lesions must always be measured (in the same anatomical plane as the baseline examination), even if they decrease in size during the study to a short axis of < 10 mm. Therefore, when lymph nodes are used as target lesions, the sum of the lesions' dimensions is not necessarily zero even if there is CR since a normal lymph node is defined as having a short axis < 10 mm. To qualify for CR, every lymph node must achieve a short axis of < 10 mm.*

**Partial response (PR)** At least a 30% decrease in the sum of diameters of target lesions, taking as reference the baseline sum diameters.

**Progressive disease (PD)** At least a 20% increase in the sum of diameters of target lesions, taking as reference the smallest sum, or nadir, in the study (this includes the baseline sum if that is the smallest in the study). In addition to the relative increase of 20%, the sum must also demonstrate an absolute increase of at least 5 mm.

Note: the appearance of one or more new lesions is also considered progression.

However, if there is progression compared with the nadir *and* response compared with the baseline examination, this is considered progression.

**Stable disease (SD)** Neither PR, CR or PD.

**Non-target lesions**

**CR** Disappearance of all non-target lesions and normalization of tumor markers. All lymph nodes must have reached a short axis of < 10 mm.

**Non-CR/SD** Persistence of one or more non-target lesions and/or tumor marker levels above the normal limits.

**PD** **Unequivocal** increase in size of existing non-target lesions or appearance of one or more new lesions.

###### Overall response:

| **Target lesions** | **Non-target lesions** | **New lesion** |  | **Overall response** |
| --- | --- | --- | --- | --- |
| CR | CR | No | **=** | **CR** |
| CR | Non-CR/Non-PD | No | **=** | **PR** |
| CR  PR | Not assessed  Non-PD or not all assessed | No  No | **=**  **=** | **PR**  **PR** |
| SD | Non-PD or not all assessed | No | **=** | **SD** |
| Not all assessed  PD | Non-PD  Any | No  Yes or no | **=**  **=** | **Unassessable**  **PD** |
| Any | PD | Yes or no | **=** | **PD** |
| Any | Any | Yes | **=** | **PD** |

Comments on lesion measurability on entry

Bone lesions:

Bone scan, PET scan or plain films are not considered adequate imaging techniques to measure bone lesions. However, these techniques can be used to confirm the presence or disappearance of bone lesions.

Lytic bone lesions or mixed lytic-blastic lesions, with identifiable soft tissue components, can be considered as measurable lesions if they can be evaluated by cross-sectional imaging techniques such as CT or MRI and if the soft tissue component meets the definition of measurability described above.

Cystic lesions:

Lesions that meet the criteria for radiographically defined simple cysts are not considered as malignant lesions (be it measurable or non-measurable).

Cystic lesions thought to represent cystic metastases can be considered as measurable lesions if they meet the definition of measurability described above. However, if noncystic lesions are present in the same patient, these are preferred for selection as target lesions.

Lesions with prior local treatment:

Tumor lesions situated in a previously irradiated area, or in an area subjected to other loco-regional therapy, are usually not considered measurable unless there has been demonstrated progression in the lesion since the local therapy. Study protocols should detail the conditions under which such lesions may be considered measurable.

APPENDIX 6: ASSESSMENT OF TOXICITY (nci-ctc v4.0 )

**ASSESSMENT OF TOXICITIES NCI-CTC V4.0**

<http://evs.nci.nih.gov/ftp1/CTCAE/About.html>

and then click "Files: Data" on "CTCAE 4.03 2010-06-14.xls"

**NEUROTOXICITY EVALUATION USING mTNS SIMPLIFIED SCALE**

Simplified mTNS scale for the evlation of Taxan-induced neuropathy

| **Parameters/ Grades** | **0** | **1** | **2** | **3** | **4** |
| --- | --- | --- | --- | --- | --- |
| Sensory symptoms | None | Limited to fingers/toes | Extend to wrist/ankle | Extend to elbow/knee | Reduced above elbow/knee |
| Motor symptoms | None | Slight difficulty | Moderate difficulty | Require help or assistance | Paralysis |
| Pin sensibility | Normal | Reduced in fingers/toes | Reduced to wrist/ankle | Reduced to elbow/knee | Reduced above elbow/knee |
| Vibration Sensibility | Normal | Reduced in finger/toes | Reduced up to wrist/ankle | Reduced up to elbow/knee | Reduced above elbow/knee |
| Strength | Normal | Mild weakness, but can overcome resistance | Moderate weakness, can overcome gravity but not resistance | Severe weakness, cannot overcome gravity | Paralysis |
| Tendon reflex | Normal | Ankle reflex reduced | Ankle reflex absent | Ankle reflex absent/others reduced | All reflexes absent |

APPENDIX 7: SUMMARIES OF PRODUCT CHARACTERISTICS FOR STUDY PRODUCTS

**SUMMARY OF PRODUCT CHARACTERISTICS – FLUOROURACILE EBEWE^®^**

<http://agence-prd.ansm.sante.fr/php/ecodex/frames.php?specid=67294648&typedoc=R&ref=R0206290.htm>

**SUMMARY OF PRODUCT CHARACTERISTICS – ELVORINE**^®^

<http://agence-prd.ansm.sante.fr/php/ecodex/frames.php?specid=62645381&typedoc=R&ref=R0178719.htm>

**SUMMARY OF PRODUCT CHARACTERISTICS – ABRAXANE**^®^

<http://www.ema.europa.eu/docs/fr_FR/document_library/EPAR_-_Product_Information/human/000778/WC500020435.pdf>

**SUMMARY OF PRODUCT CHARACTERISTICS – GEMZAR 1000 mg^®^**

<http://agence-prd.ansm.sante.fr/php/ecodex/frames.php?specid=61928914&typedoc=R&ref=R0233056.htm>

**SUMMARY OF PRODUCT CHARACTERISTICS – ONIVYDE 5 mg®**

http://www.ema.europa.eu/docs/fr_FR/document_library/EPAR_-_Product_Information/human/004125/WC500215029.pdf

**NON-EXHAUSTIVE LIST OF CONTRAINDICATIONS IN CASE OF NAL-IRI TREATMENT (ONIVYDE®)**

|  | **Inhibitors** | **Inducers** |
| --- | --- | --- |
| **CYP3A4/ UGT1A1** | - Ritonavir, indinavir, lopinavir, nelfinavir, ritonavir, saquinavir, telaprevir - Cobicistat - Nefazodone - Azole antifungals, (ketoconazole, itraconazole, fluconazole, posaconazole, voriconazole) - Macrolides (erythromycin, clarithromycin, telithromycin, josamycin) - Amiodarone - Diltiazem, verapamil - Grapefruit (fruit or juice) - Atazanavir - Gemfibrozil | - Saint john’s wort - Anticonvulsivants (carbamazepine, phenobarbital, phenytoïne, oxcarbazepine...) - Anti-infective (rifampicin, rifabutin, efavirenz, nevirapin, griseofulvin) |

Information from ONYVIDE©’s smPC and from ANSM (04/05/2018): <http://ansm.sante.fr/Dossiers/Interactions-medicamenteuses/Interactions-medicamenteuses-et-cytochromes/(offset)/1>

APPENDIX 8: Serious Adverse Event Report Form

| **PRODIGE 61 – FFCD 1702 – FUNGEMAX**  **SERIOUS ADVERSE EVENT REPORT FORM (SAE)** | | | | | | | | | | **Page 1/3** | |
| --- | --- | --- | --- | --- | --- | --- | --- | --- | --- | --- | --- |
| **SPONSOR : FFCD PRINCIPAL INVESTIGATOR : Pr Julien TAIEB**  **Study title : Randomized phase II study comparing 5FU/LV+Nal-IRI, gemcitabine+Nab-paclitaxel or a sequential regimen of 2 months 5FU/LV+Nal-IRI followed by two months of gemcitabine+Nab-paclitaxel, in metastatic pancreatic cancer**  **N° EudraCT** : 2017-004309-41 | | | | | | | | | | | |
| **Author of the declaration :** Dr  – Pr  - CRA  - Other , specify : ……………………………….  Name : Center :  Phone : Fax :……………………………………………… | | | | | | | | | | | |
| **SAE n° :**  **Type of report :  initial  follow-up n° :**   **Date of report :**  | | | | | | | | | | | |
| **space reserved for data center (CRGA)**  **Date of reception** :  **Sponsor reference for the event  :** | | | | | | | | | | | |
| **Patient N°**   | | **Patient’s initials :** -α | | | **Sex :**  Female  Male | | | | **Arm A - Nal-IRI+5FU/LV alt Nab- paclitaxel + Gemcitabine**  **Arm B – Nal-IRI+5-FU/LV**  **Arm C – Nab-paclitaxel + Gemcitabine** | | |
| **Date of birth :**  **Inclusion date :**  | | | | | | | | |  |  |  |
| **Weight (kg) : ** **Height (cm) :**  | | | | | | | | | | | |
| **Serious adverse event :**  **_____________________________________________________**  **_____________________________________________________** | | | | | | | | | **Date of start**   Ongoing  **Date of end**  | | |
| **Seriousness criteria** | | | | | | **Grade/severity** | | | | | |
| hospitalization (or prolongation)  medically significant  durable or significant disability or incapacity  life-threatening  death  congenital anomaly or fetal malformation | | | | | | **Coded as NCI-CTC 4.0** α  **If not applicable, specify :**  1 = mild  2 = moderate  3 = severe  4 = life-threatening  5 = death due to SAE | | | | | |
| **Outcome** | | | recovered/resolved without sequelae  recovered/resolved with sequelae  recovering/resolving  not recovered/resolved  death | | | | | | | | |
| ***If hospitalization*** | | | Date of admission :  ongoing  Date of discharge :  | | | | | | | | |
| ***If death*** | | | Date of death :  Death cause | | | | | | | | |
| Specify :  Death related to SAE  Death for which SAE may have contributed  Death related to SAE | | | | | | | | | | | |
| **Description**  **Please describe below the chronological sequence of events including the history of the disease and the relevant concomitant diseases existing in the context of the Serious Adverse Event.** | | | | | | | | | | | |
| **PRODIGE 61 - FUNGEMAX - FFCD 1702**  **SERIOUS ADVERSE EVENT REPORT FORM (SAE)** | | | | | | | | **Page 2/3 SAE n°: **  initial  follow-up  **Patient N° : ** | | | |
| **If arm A, specify treatment sequence when SAE occurs:**  **Nal-IRI+5FU/LV**  **Nab-paclitaxel + gemcitabine** | | | | | | | | | | | |
| **Drug** | **Administration** | | | **Last dose** | | | **Treatment modification due to SAE** | | | | |
| **Nal-IRI**  Not applicable | Date of first administration:    Date of last administration before SAE :    D1  D15  Cycle n°: αα | | | _________ mg | | | Dose not changed  Dose reduced, specify : new dose : _________ mg  Temporary withdrawal, specify date of reintroduction :    Definitive withdrawal, specify date :   | | | | |
| **5FU**  Not applicable | Date of first administration:    Date of last administration before SAE :    D1  D15  Cycle n°: αα | | | _________ mg | | | Dose not changed  Dose reduced, specify : new dose : _________ mg  Temporary withdrawal, specify date of reintroduction :    Definitive withdrawal, specify date :  | | | | |
| **Acide Folinique**  **D-L 9 L 9**  Not applicable | Date of first administration:    Date of last administration before SAE :    D1  D15  Cycle n°: αα | | | _________ mg | | | Dose not changed  Dose reduced, specify : new dose : _________ mg  Temporary withdrawal, specify date of reintroduction :    Definitive withdrawal, specify date :   | | | | |
| **Nab-Paclitaxel**  Not applicable | Date of first administration:    Date of last administration before SAE :    D1  D8  D15  Cycle n°: αα | | | _________ mg | | | Dose not changed  Dose reduced, specify : new dose : _________ mg  Temporary withdrawal, specify date of reintroduction :    Definitive withdrawal, specify date :   | | | | |
| **Gemcitabine**  Not applicable | Date of first administration:    Date of last administration before SAE :    D1  D8  D15  Cycle n°: αα | | | _________ mg | | | Dose not changed  Dose reduced, specify : new dose : _________ mg  Temporary withdrawal, specify date of reintroduction :    Definitive withdrawal, specify date :   | | | | |
| Disparition of event after stop or dose reduced of suspected drugs :  Yes  No  Unknown  Not applicable  Recurrence of event after reintroduction of suspected drugs :  Yes  No  Unknown  Not applicable | | | | | | | | | | | |
| **PRODIGE 61 - FUNGEMAX - FFCD 1702**  **SERIOUS ADVERSE EVENT REPORT FORM (SAE)** | | | | | | | | | **Page 3/3 SAE n°: **  initial  follow-up  **Patient N° : ** | | |
| **Concomitants drugs : (regular treatment of the patient or other drugs received within 15 days)** | | | | | | | | | | | |
| **Drugs** | **Date of start** | | | **Ongoing** | | **Date of end** | | | **Dose** | | **Indication** |
|  |  | | |  | |  | | |  | |  |
|  |  | | |  | |  | | |  | |  |
|  |  | | |  | |  | | |  | |  |
|  |  | | |  | |  | | |  | |  |
|  |  | | |  | |  | | |  | |  |
|  |  | | |  | |  | | |  | |  |
|  |  | | |  | |  | | |  | |  |
|  |  | | |  | |  | | |  | |  |
|  |  | | |  | |  | | |  | |  |
|  |  | | |  | |  | | |  | |  |
|  |  | | |  | |  | | |  | |  |
| **Causality assessment** | | | | | | | | | | | |
| **Nal-IRI :**  **related**  **not related**  **doubtfully related** or :  **not applicable** | | | | | | | | | | | |
| **5FU :**  **related**  **not related**  **doubtfully related** or :  **not applicable** | | | | | | | | | | | |
| **Folinic Acid :**  **related**  **not related**  **doubtfully related** or :  **not applicable** | | | | | | | | | | | |
| **Nab-paclitaxel :**  **related**  **not related**  **doubtfully related** or :  **not applicable** | | | | | | | | | | | |
| **Gemcitabine :**  **related**  **not related**  **doubtfully related** or :  **not applicable** | | | | | | | | | | | |
| **If the causality assessment between SAE and study drugs are « not related », which is, to your opinion, the cause of SAE ?** (tick the appropriate box(es))  Progression of cancer  Preexisting condition, specify : ___________________________________  Concomitant drug, specify which one : ______________________________  Other illness, specify : ____________________________  Other, specify : ___________________________________ | | | | | | | | | | | |

**PLEASE ATTACH ANONYMIZED HOSPITALIZATION REPORT, AND, IF NEEDED BIOLOGICAL TESTS, COMPLEMENTARY EXAMS…**

**Form to fax at Data Center CRGA Dijon Fax : 03 80 38 18 41**

**DATE : NAME : SIGNATURE :**

APPENDIX 9: RULES FOR PUBLICATION FOR PRODIGE TRIALS

**PRODIGE RULES FOR PUBLICATION**

**The rules for publication that will be used for this study will be those in effect at the time of the last inclusion.**

(partnership version of May 3, 2012)

**PRODIGE RULES FOR PUBLICATION**

Having a good-quality journal publish the PRODIGE trials quickly is a vital objective for disseminating advances in treatment. The PRODIGE coordination committee is responsible for publication, deciding on:

- when the preliminary and definitive results of a trial are published.

*All information arising from trials is confidential, at least until the sponsor, coordinating investigator and statistician have finished the appropriate analysis and verification of the trial.*

- the composition of the drafting committee (which generally comprises a maximum of seven members).

The coordination committee may delegate these functions to the trial coordinator.

It validates the choices made and ensures deadlines are met. If the coordination committee does not respond within 1 month of submission by the drafting committee, this may be taken as approval.

1. The drafting committee comprises:

- The committee chair as defined by internal regulation
- The most important contributors

In collaborative, national and international trials, if the other associations have enrolled at least 10% of the sample population, the drafting committee comprises a representative chosen from among the investigators of each of the other associations.

Any coordinator from a country or association who has not enrolled any patients will not be on the drafting committee or be an author of the publication, but will be thanked at the end of the article.

1. The lead author undertakes to submit the article for publication within a time period specified by the coordination committee. This period must be no more than 1 year after the trial has closed. If the lead author cannot do this, the coordination committee may designate a new author who becomes the lead author. To facilitate the writing of articles arising from the trials, a medical writer may be called on and writing workshops may be organized for the lead author in collaboration with the statistician.
2. Before each publication, the study project manager sends the coordination committee the planned list of authors accompanied by a table of inclusions for each investigator center.

The coordination committee validates the number and order of authors before each publication in accordance with the PRODIGE rules for publication. If far from the coordination committee's meetings, validation is made by email. A period of 7 days without reply implies approval.

1. **Title of the publication and oral communications**: the title of the trial must be PRODIGE 61, followed by any name given by the sponsor group.
2. **The publication's authors** are ordered according to work contributed and number of patients enrolled:

- A lead author
- A limited number of investigators, by order of participation. There is generally one investigator per center but the steering committee may decide to name two investigators for some centers. This rule may be weighted so that some small- and medium-sized centers that contribute significantly to enrollment may appear as authors. The coordination committee will approve any such weighting so that no party is wronged.
- If he or she is not the lead author, the trial coordinator, or any person who contributes in a major way to the conception and/or conduct of the trial (such as a co-coordinator), is generally the last author. The coordination committee will decide should there be any disagreement.
- The maximum number of authors permitted by the journals will be used.
- Regardless of the number of patients included, at least one author will represent one of the two partners (FFCD or UNICANCER-GI).
- In spin-off publications and ancillary studies, the authors may be different from those of the original article and reflect the specialty in question – for instance, in trials on radiochemotherapy, an article on radiotherapy may be signed by the co-investigator radiotherapists of the centers that enroll. The first author of the original article is therefore the last author of the spin-off publication.
- The PRODIGE partnership is mentioned in the title or after the authors. If the trial is a collaborative study, the first association mentioned is the one that initiated the trial. The others are mentioned if they included at least 5% of the patients, in order of their contribution.
- For trials sponsored or managed by the FFCD, a member of the INSERM U1231 research unit will be the second last author and will be mentioned as having "equally contributed" if this member is not the lead author so that this work done by the INSERM is taken into consideration.
- For trials sponsored or managed by UNICANCER, a representative of the sponsor will be included in the authors.
- The statistician will be named among the authors, generally after the third place. The statistician may be the first or second author of a spin-off publication.

All contributors who do not appear among the authors are mentioned at the end of the article. Study managers (such as the project manager or data manager) are also mentioned.

One of these may occasionally be mentioned as an author if the PRODIGE coordination committee believes it to be justified.

Partners are thanked, as are the patients and their families.

The authors and sponsor are sent a copy of the manuscript for review before submission to a journal. To have their opinions taken into account, they undertake to reply within 15 working days, or 30 days in the summer.

1. **Oral communications** based on the trial results:

An investigator may, after obtaining the approval of the PRODIGE coordination committee and trial steering committee, present in his or her own name all or part of the trial results in an oral presentation. The authors are generally the same as in the written article, but the order of authorship may vary across articles and communications, and also depending on the conference where the presentation is being made. In certain cases, such as multidisciplinary studies or pathological, biological, endoscopic or imaging studies conducted alongside a therapeutic trial, other authors may be chosen depending on their work. The name of the trial remains PRODIGE 61 (see § 3) and the other associations will be mentioned if necessary.

1. **These rules must appear in the appendices of all PRODIGE trial protocols.**


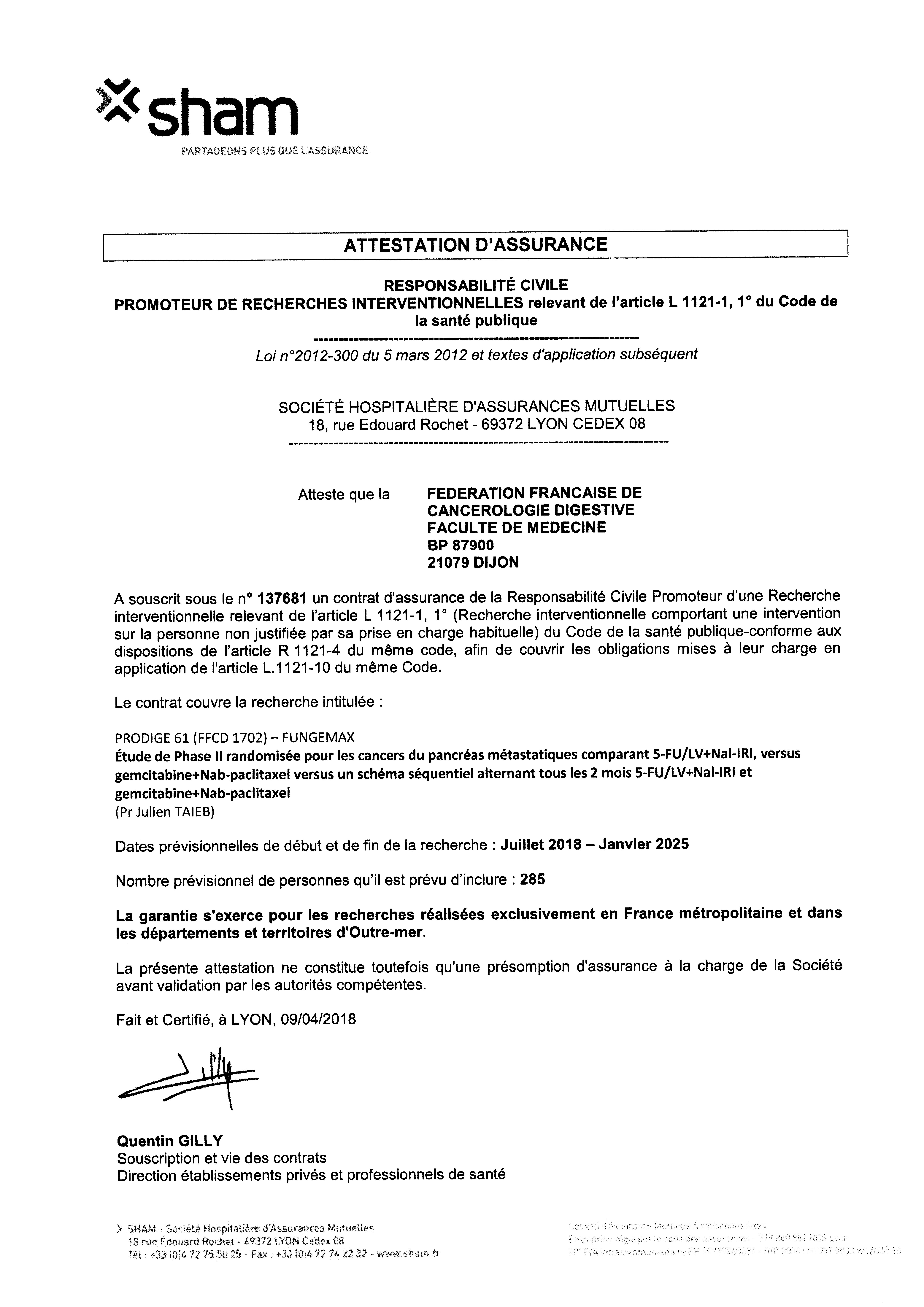
APPENDIX 10: INSURANCE CERTIFICATE


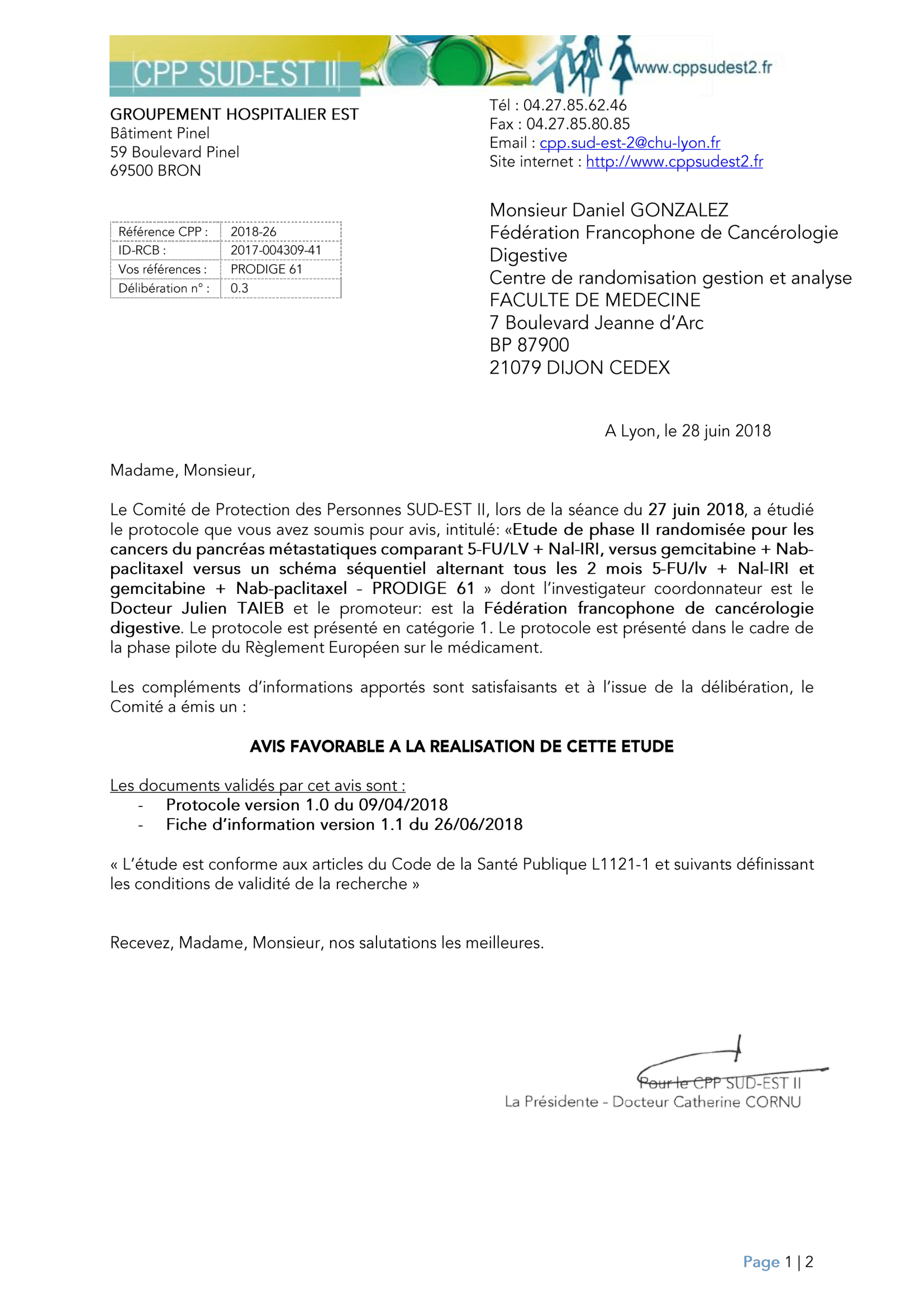
APPENDIX 11: APPROVAL OF THE IRB

**APPENDIX 12: ANSM AUTHORIZATION**


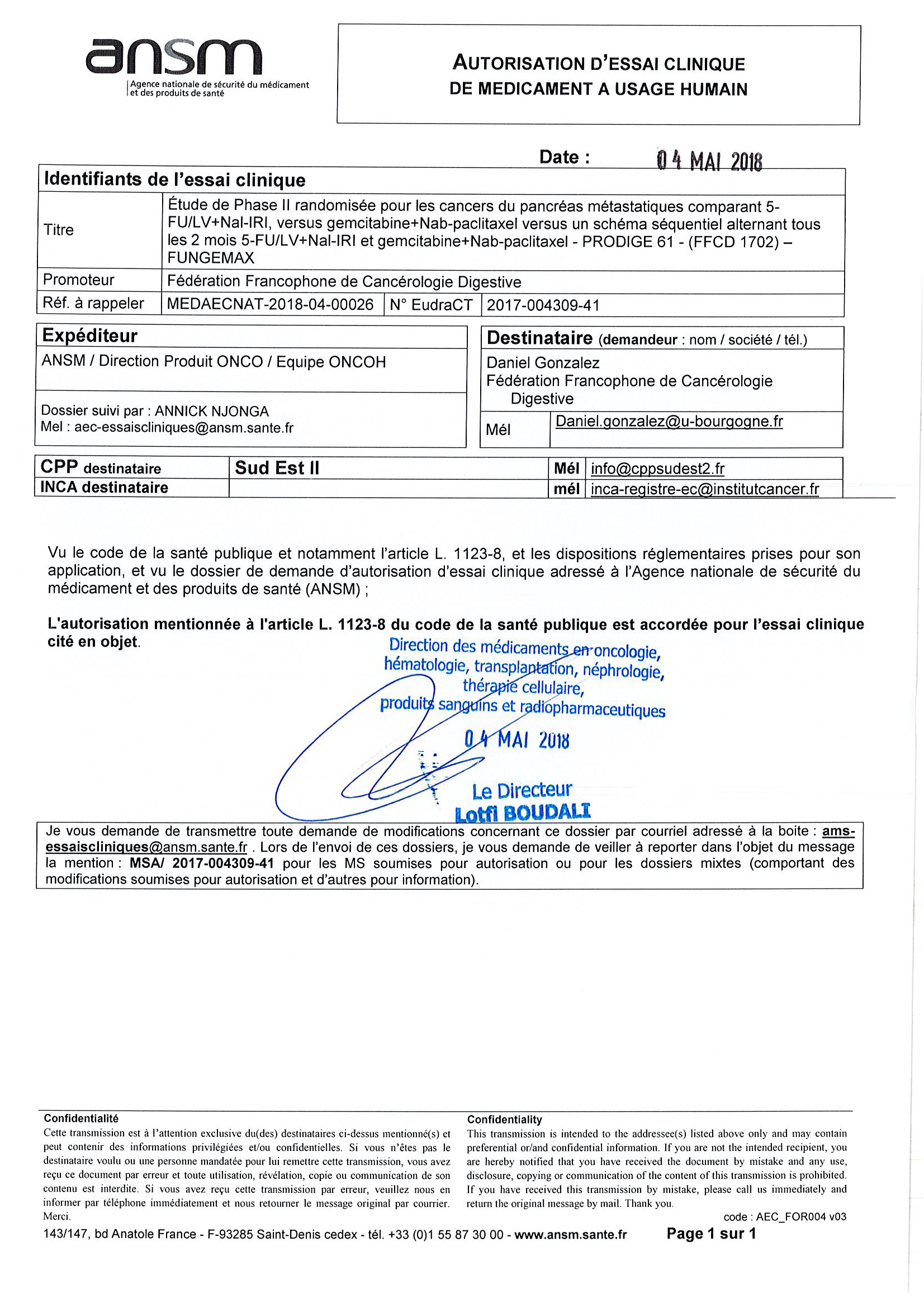


Study Protocol Supplementary File

**PRODIGE 61 – FUNGEMAX Trial**

**Randomized phase II study comparing 5-FU/LV + nal-IRI, gemcitabine + nab-paclitaxel, or a sequential regimen in metastatic pancreatic cancer**

Protocol reference: PRODIGE 61 – FUNGEMAX (FFCD1702)
Sponsor: Fédération Francophone de Cancérologie Digestive (FFCD)
Latest protocol version: **Version 2.0 – July 27, 2022**

This supplementary document summarizes all protocol modifications implemented since the initial study protocol version.

**Summary of Protocol Amendments**

**Protocol Version at Study Launch**

**Version 1.1**

**Amendments from Version 1.1 to Version 1.2**

The following modifications were implemented:

**Secondary endpoints**

- Addition of two secondary endpoints:
  - **Depth of response**
  - **Early tumor shrinkage**

**Treatment administration**

- Modification of gemcitabine infusion protocol:
  - Initial protocol:
    - Gemcitabine **1000 mg/m² in 500 mL normal saline infusion at a fixed dose rate of 10 mg/m²/min**
  - Revised protocol:
    - Gemcitabine **1000 mg/m² in 500 mL normal saline infusion administered over 30 minutes**

**Follow-up assessments**

- **Quality-of-life (EORTC QLQ-C30)** questionnaires are no longer collected after disease progression.
- **INR monitoring** was removed from routine protocol assessments.

**Amendments from Version 1.2 to Version 1.3**

The following changes were introduced:

**Eligibility criteria**

- Addition of an inclusion criterion requiring **assessment of dihydropyrimidine dehydrogenase (DPD) deficiency** before study entry.

**Statistical design**

- Clarification of the **global type-I error rate (alpha)** for the trial, defined as **10% (two-sided)** for the comparison between experimental arms and the reference arm.

**Amendments from Version 1.3 to Version 1.4**

**Treatment dosing**

- Modification of the **liposomal irinotecan (nal-IRI) dose**:
  - Previous dose: **80 mg/m²**
  - Updated dose: **70 mg/m²**

This modification was implemented to improve treatment tolerability and align with emerging safety data.

**Amendments from Version 1.4 to Version 2.0**

**Study conduct**

- **Extension of the recruitment period** to ensure completion of the planned sample size.

**Contraception requirements**

- Clarification of contraception requirements for participants of reproductive potential:
  - Women of childbearing potential and men with partners of childbearing potential must use **effective contraception during the study and for 7 months after the last administration of study treatment.**

**Final Protocol Version**

The **final protocol version used for the conduct of the study was Version 2.0 (July 27, 2022)**.
